# Systematic modelling of a large-scale, historicised, well-detailed building land use, with the fusion of massive and heterogeneous datasets

**DOI:** 10.1371/journal.pone.0359346

**Published:** 2026-10-07

**Authors:** Maxime Colomb, Julien Perret

**Affiliations:** 1 ASCII, Inria, CMAP, CNRS, École Polytechnique, Institut Polytechnique de Paris, Palaiseau, France; 2 LASTIG, Univ Gustave Eiffel, IGN-ENSG, Saint-Mande, France; Yunnan University, CHINA

## Abstract

Buildings are the primary venues for human activity. Generating precise numeric building models is of great interest for many applications. At regional and national scales, many different formalisms are employed to represent the various components of buildings, yet the building land use is frequently oversimplified — even when mixed-use buildings are considered. Inferring a realistic building land use, defined as an inventory of the distinct functional spaces hosted by a building, brings valuable knowledge for building models. Manual measurement can produce highly realistic interior models, but such approaches are labour-intensive and must be conducted on a case-by-case basis. We introduce a fully automated workflow that systematically simulates a well-described building land use by integrating three complementary tasks: (1) use-oriented building object modelling – defining building objects that capture building land use, (2) activity census and conflation – aggregating and reconciling activity records from heterogeneous datasets, and (3) logical attribute generation – deriving detailed attributes for housing units and activities in relation to the characteristics of their host building and other environmental factors. The workflow adapts to varying levels of data availability and attribute richness, and it supports “recent-period historicisation” (i.e., reconstructing past states of the built environment). Because the entire pipeline is automated, it can be deployed at a large scale; we demonstrate its application to an entire country. We evaluate the method through multiple validation procedures, including comparisons with ground truth measurements, showing that the approach reliably reproduces building land use.

## 1 Introduction

### 1.1 Context

Buildings are the most primitive elements of urban scenes because they define the principal physical boundaries that separate outdoor from indoor space. Most human presence and interaction occur inside buildings [1]. They host a large proportion of sites of human activity, such as housing units, offices, shops and most urban leisure activities. Consequently, buildings are central to the relationship between humans and their urban environment.

Buildings are defined and modelled in accordance to the intended application and its scale. Models that require immersive 3D city views need well-described building envelopes, often with façade reconstruction. On the other hand, models that reproduce human activity at a precise and individual level (e.g., ABM, LUTI) require a description of the building land use* (notions followed with an * are defined in the Glossary in annex A). Their traditional formalism for representing buildings has often been a single geometry, either punctual or polygonal. Building land use is frequently oversimplified to a primary–and sometimes secondary–use for the whole building, represented as a textual attribute. This formalism is not suitable for cities containing a complex or historic urban fabric.

The New Urbanism charter [2] recommends developing mixed use of buildings in cities. Models of building land use need a precise description of a building’s various uses. Changes in a building’s use, encouraged by urban trends such as the liquid city concepts [3], indicates that building land use is changing at an unpredictably high rate [4]. Models must then be capable of quick updates and accommodate multiple types of datasets. Building functions have been inferred by many research studies at an aggregated level [5]. As many models and frameworks focus on the representation of the physical interior of buildings [6], collecting additional urban objects, such as localisation of housing units* or activities*, and matching them to buildings, could further refine their land use description at an individual scale.

### 1.2 Stakes

Yet, simulating a precise, exhaustive, well-described, historicised*, spatially realistic building land use remains an ongoing research challenge. Such a model would benefit other urban models in several ways.

Microscopic sociospatial modelling could take advantage of a more spatially complex and richly described building land use. Modellers often seek greater accuracy and realism in the definition, completeness and variety of the information they manage. Enhancing the realism of building land use at an individual scale could be a highly effective way to improve the realism of individual-level interactions. Knowing which activities are hosted in single buildings is valuable for assigning specific tasks to human agents in agent-based models. A descriptive land use should generate attributes concerning those activities, such as type, opening hours, the number of workers employed, or even the area occupied by such activities. Such knowledge should help allocate the appropriate number of agents to a given place and task, allowing us to compute densities of people living, working and spending leisure time, as well as occupancy saturation, for each building in the model.

Increasing knowledge about building land use would also benefit various urban generation models and allow systematic deployment at large scale. Indoor 3D reconstruction models could be guided by such information. Urban heat island generators that focus on anthropogenic heat [7] could model a more detailed occupation of buildings by human activities. The definition of activities in buildings could also be used to generate multiscale energy consumption estimates [8].

3D building reconstruction generators could use a building land use model to infer missing information about particular building elements or to choose appropriate urban fabric grammars for procedural generation. Building Information Modelling (BIM) reconstruction projects could gain clues about the nature and amount of detail to add when converting geographic information system (GIS) data into fully detailed BIM data, or more generally when interfacing GIS and BIM [9].

Because building land use changes frequently, its generation should incorporate a rapid-update system to produce results across multiple time periods. Specific spatiotemporal analysis could benefit from this precise, large-scale knowledge with historicised versions, such as real estate analysis [10], housing supply studies [11], commercial evolution analysis [12] or office vacancy assessments [13].

Finally, Volunteered Geographical Information (VGI) platforms could compare their data to such a model to help update specific information.

### 1.3 Challenges

Modelling a well-defined building land use at a large zone is challenging in multiple ways. Urban models cannot access an exhaustive and large-scale building land use database because such information is sparse and spread across numerous datasets. In large geographic collections, the building object is often described as a geometry with few attributes. Sometimes, one or two destinations for the building are described in its attribute information, but these are usually not exhaustive and are insufficient to model detailed characteristics of the different kinds of places. Even with an exhaustive list of functional space*, it is not possible to obtain a correct estimation of the area covered by each of them, to know whether a registered activity occupies a premises*, or what is the use of such *functional spaces*.

Methods based on sensors are mostly applied case-by-case, as the measurement tool must enter every building in order to scan rooms. Such methods cannot be deployed systematically. VGI-based methods are also applied to a few cases. While their application could be encouraged by communes and yield a complete dataset within a few years, data consistency would remain a strong concern. BIM translations are dependent on the data available, which may exist for new building projects, but they are too complex to construct for all existing buildings.

Census and statistical information about the economics and population could bring valuable data for building land use. They could follow multiple formalisms, scales, geometric types, or census recording objects. They also come from multiple types of sources (authoritative datasets, local governance databases), and the hypothesis framing their creation differ. Their fusion must then be adapted to each context.

The historicisation of data is a relevant topic in data sciences. Datasets are only representative of a given date. The interpolation of the changes between two dates requires a deep understanding of the underlying mechanisms driving system evolution and dedicated simulation models. It is therefore necessary to study each system to infer the model’s state at each wanted date.

The availability of input data is also an important topic. The created model must be generic in order to allow adaptability to different data contexts. In order to be extended to new geographic zones, enriched by various data and to benefit from community improvement, data and technologies must be available under a free and open source license.

To our knowledge, no simulation method that responds to all the previously cited information and generates plausible indoor configurations has been proposed.

### 1.4 Goals

In this paper, we present a fully automated workflow that simulates a highly detailed building land use at the building level (Fig 1). The model can be deployed systematically; it has been applied to the 34 883 communes of metropolitan France and its 47 million buildings. It provides a realistic snapshot at a specific point in time. The target date may differ from the dates of input datasets, thanks to historicisation of underlying processes.

**Fig 1:**
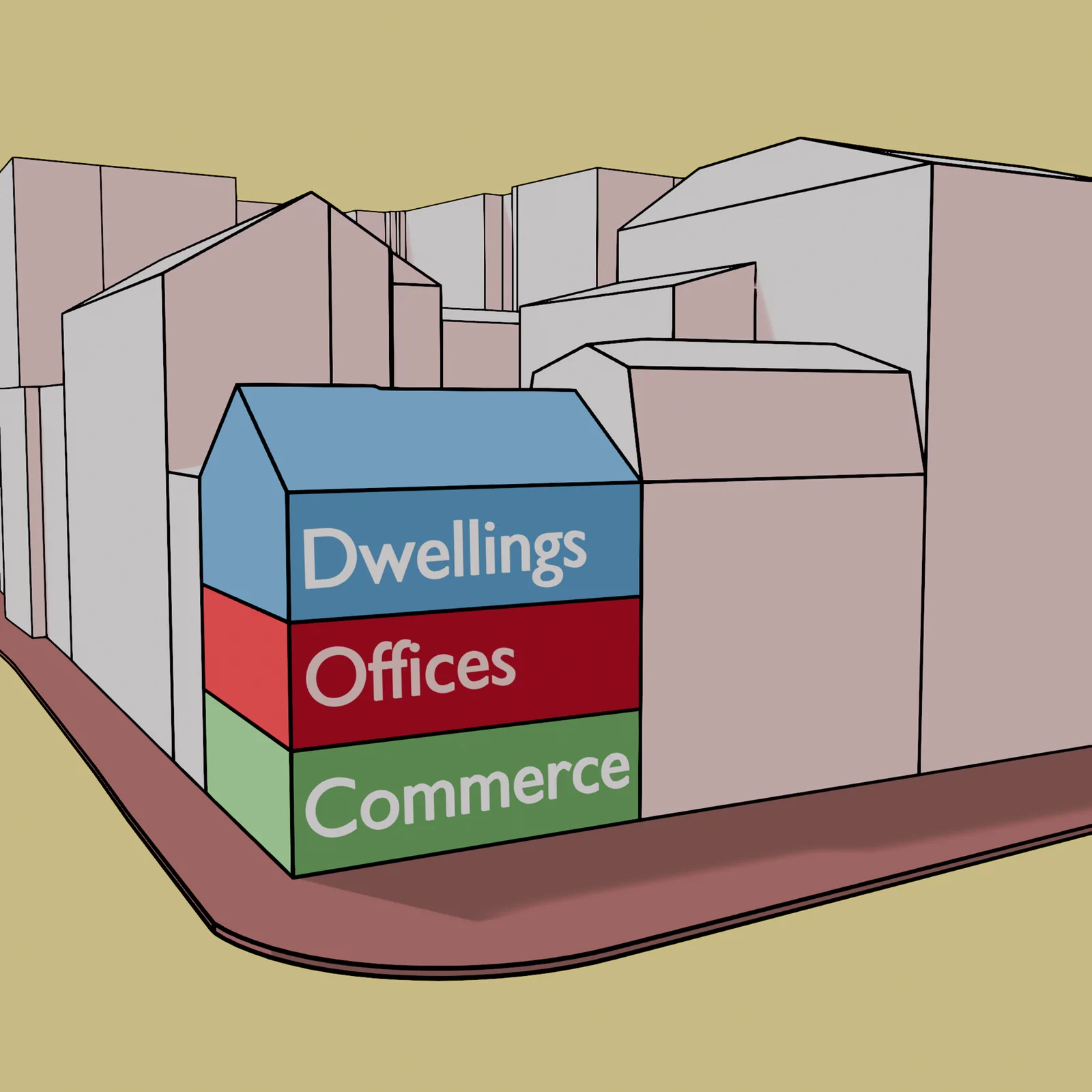
Example of a 3D representation of model’s output.

As building definitions usually focus on their geometry, a dedicated definition is adopted to accommodate mixed-use possibilities. *Functional spaces* populating buildings fall into two categories: *housing units* for living purposes or *activities*, which may ultimately occupy a *premises* and receive the public. A well-described and exhaustive list of *activities* is then collected and conflated.

Multiple data fusion processes are designed to merge different kinds of datasets and simulate synthetic information where it is missing. As input datasets follow different temporal dynamics and historicisation processes, specific data inference techniques are applied. Because different releases of the same data can model information differently, the presented models must be adapted to varying availability and quality of input data, which depends on the national and local context. A minimal amount of data is necessary, but every process can be enriched when richer data are available. Since the first implementation is built upon French data, it could be adapted to equivalent data from other countries or new data sources. All data used are available under open licenses, and the model itself is released under an AGPL license, allowing users to reproduce, use, and improve the current model.

This model is already in active use within the ICI epidemiological simulation project [14,15]. It could also be used to analyse the mutation of activity sectors, to enhance the objectives and locations of trips in transportation models, or by any model requiring a precise and geolocated description of human activity.

This model does not provide a 3D inner organisation of buildings, but an inventory of what functional spaces a building hosts. It could therefore be used as an input for automatic reconstruction algorithms. It has been developed in a French context, where datasets are numerous, diverse, detailed, and descriptive. The authors are currently working on its application to generic European datasets. Further adaptation will however be required for contexts where fewer data are available and their quality is not consistent.

The model is composed of three sequential, distinct, and relatively independent tasks:

1. **Building model:** Model a particular building object, infer missing values, and merge primitive building blocks* into building groups* in order to consider a common interior space.
2. **Activity collection and conflation:** Collect, sort, match and merge all activities that are hosted by the buildings.
3. **Building land use enrichment:** Gather descriptive information about the *functional spaces* within the building, either through collection or simulation, relative to their number, their types, their potential use and the building’s geometry.

Fig 2 depicts these tasks and their requirements in terms of data and input parameters.

**Fig 2:**
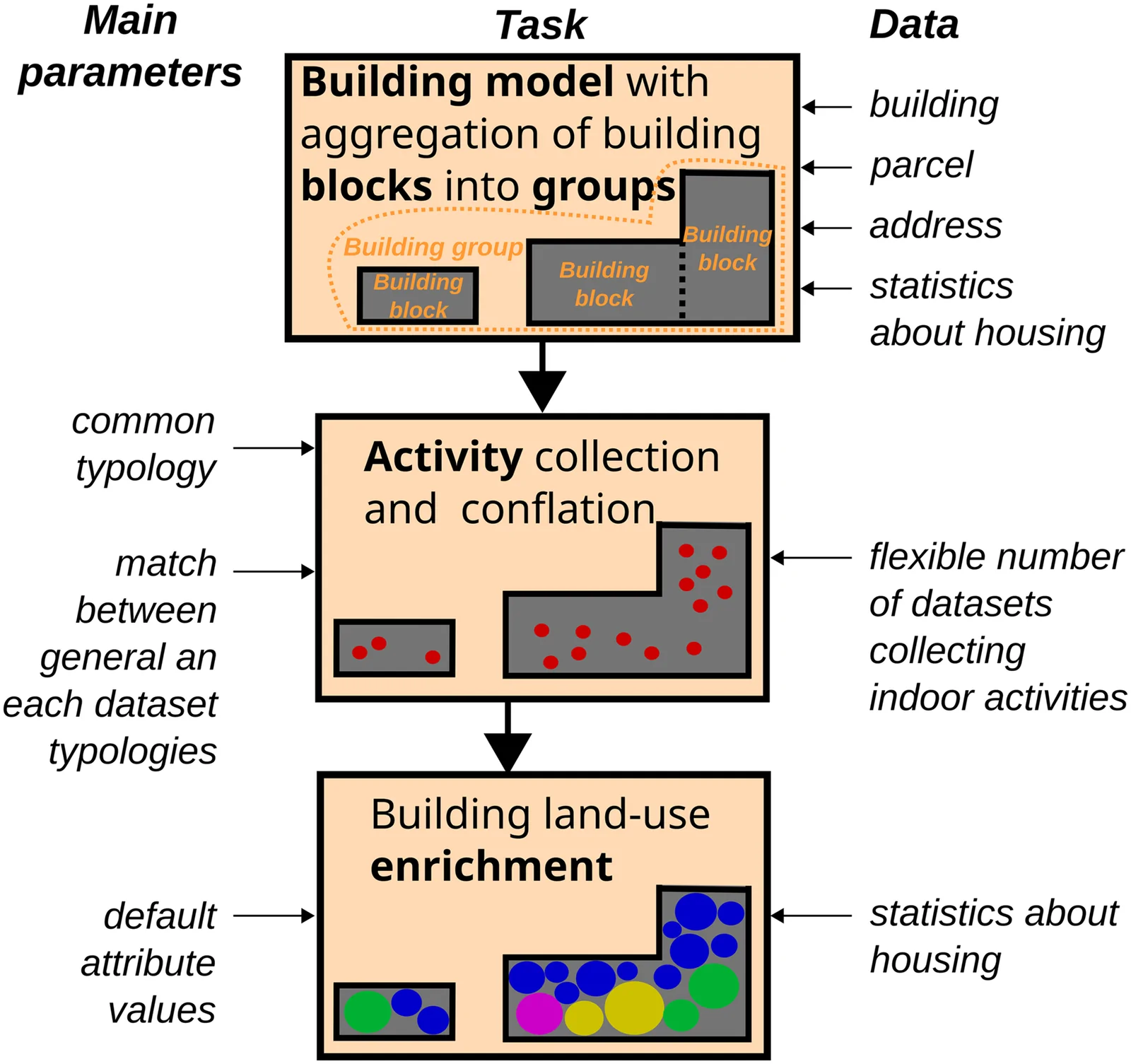
Tasks of the presented model.

This article is organised as follows. Section 2 first reviews related work and how the global problem and those individual tasks have been handled in various research domains. Section 3 then presents a resolution method, including data description and algorithm explanations. Tests and validation of each task are undertaken in Section 4, and the validation of the whole model by comparing model results with ground truth data is presented in Section 4.4 Systematic runs of the model are finally shown in Section 5.

## 2 Related work

The aim of this article is to develop a realistic, systematic, well-defined model of building land use. This would be achieved by allocating activities and housing units to properly defined buildings. Simpler models could be applied to contemporary cities, which are built upon a more functional urbanism, or to suburbs, where each building has been designed for a particular use. In the context of historic European cities, the activities hosted in a single building can be very diverse and spatially spread in multiple *building blocks*. A realistic model for such cities therefore cannot rely on purely statistical methods. To our knowledge, a detailed, high-fidelity census to model the mixed building land use has not yet been proposed. First, an analytical review of works modelling mixed-use buildings is carried out.

### 2.1 Modelling building land use

Modelling building land use often consists of labelling buildings with one or more presumed uses [5]. provides a complete state of the art about the major studies modelling building functions. Table 1 summarises works that identify individual building land use with mixed use.

**Table 1:** Summary of works modelling mixed use of building land use.

Reference	Objective / particularities	Input data	Technique	Spatial scales	Reproductibility	Limitations
[16]	infer building land use with human mobility and street-view images	building footprint from remote sensing analysis – social data (app usage – taxi GPS records) – street views	image classification – probabilistic inference	individual building of a Chinese city	no code available	inconsistency of social datasets – low accuracy in results – no details about building land use
[4]	assign large classes of building use that can be combined	VGI POI dataset – remote sensing images	machine learning – remote sensing	continuous building footprint on four Chinese urban areas	no code – data available on request	land use definition has a low descriptive scope
[17]	clustering of zone functionalities with massive heterogenous datasets	social data (app) and POI	clustering individual usage of the city	individual buildings on a Chinese city – private datasets	no code available	inconsistency of social dataset
[18]	infer building uses by population density and mobility	social data (taxis) – population records	statistical inference and clustering	clustering spatial interaction	no code available	inconsistency of social dataset – low accuracy in location and classes
[5]	assign POI to buildings and infer missing POI data	building footprint – remote sensing and street-view images	machine learning – geospatialised data mining	medium Chinese city	parts of data and code available – no licence – requires proprietary software	mixed function is a category and no further described – not fully automatised

One of the inconveniences of image-analysis approaches (remote sensing, street-view images) is their limited accuracy. Building detection on remote sensing imagery may be suitable for recently built urban centres, but it performs poorly in complex and historic urban fabric. These methods are also very costly when a large scale, consistent and precise deployment is required. Consequently, they have high latency for updates and models depending on them cannot update quickly. Mining massive geospatial datasets is a suitable approach for our problem. The reviewed works that adopt this approach either don’t model mixed use of building land use [19] or propose a combination of very large classes [16,17]. Most existing studies do not treat buildings as single objects with proper semantics; instead, they regard each building as the recipient of a continuous value calculated for the whole city [16,17]. All analysed studies are typically deployed on a single large city, occasionally on several urban areas [20], but never on a region as extensive as an entire country that encompasses both urban and nonurban fabric. Reproducibility is also a concern: social data are often expensive or private, very few codebases have been made publicly available, and none of the works introduce reusability concepts.

As no study addresses all the challenges listed in Section 1.3, the three-step approach proposed in Section 1.4 is adopted. Because steps 1 and 2 concern distinct fields of research, a summary of related work is presented separately (Sections 2.2 and 2.3). The third step consists of in-situ attribute setting, and no specific state-of-the-art was required to address this topic.

### 2.2 Modelling building objects

First of all, a definition for a building object must be set. The OpenStreetMap project (OSM) [21] definition is [22]:

a building is a man-made structure with a roof, standing more or less permanently in one place.

The definition is vague when establishing proper building objects. The French authoritative database for geographical objects, namely *BD_Topo*®, specifies that a new building is created each time a difference higher than 3 meters is observed in a continuous building [23]. This leads to a definition rather focused on the physical envelope of the building than on its *functional spaces* and its inner organisation. A new definition of a primary building object could be proposed and serve in this work:

*a building is a human-made structure comprising a set of enclosed rooms that are connected together, physically or not, and that contain one or more functional spaces intended for a human use*.

On that basis, this model needs to build a building object serving its needs. Much work focuses on the model of distinct aspects of buildings with different objectives. Table 2 summarises such works.

**Table 2:** Summary of works modelling building objects.

General objective	Building object	Building envelope	Storeys	Floor area	Housing units
Model object	[24–26]	[27]	[5,26,28,29]	[5,26,29]	[30]
Analyse/comparison of methods in large dataset	[25,31,32]	[31]	[33,34]	[33]	
Historicisation	[35]				

Many research projects have worked on various parts of a building, but none had a complete modelling approach on building definition, modelling of geometries, generation of attributes, and analysis of the deployed methods.

Attribute generation is often set using diverse input data. In order to estimate floor area per building [5,29], both apply a machine learning algorithm using a pre-labelled dataset. Data sources include 3D geometry reconstruction, photogrammetry analysis on LIDAR façades, and property registries for [29] only. Population can be allocated to buildings [30] and a number of housing units can be inferred from this information. Nevertheless, modelling distinct housing units is not the goal of the reviewed literature.

Most works performing analysis compare OSM buildings with authoritative datasets [31,32]. The 2013 dataset studied in [31] has already been evaluated to have an acceptable completeness, but many attributes (such as height, stories) were missing.

Furthermore, historicisation of buildings has rarely been handled. Methods produced by the SUBDENSE [35] project could be used to infer snapshots for uncovered time periods.

### 2.3 Modelling points of interest

Two different types of *functional spaces* within buildings are planned to be modelled: *housing units* and *activities*. The latter can concern every type of place that is used for commercial, professional, associative or leisure purposes. Many studies focus on Points of Interest (POI), which are defined as representing an interesting location for different audiences [36]. Therefore, the activity definition used in this project encompasses the POI definition and includes every private place that is not used for residential purposes (such as offices or vacant shops). The extensive knowledge regarding POI classification, data gathering and fusion, can be reused for activity modelling.

Multiple sources of activities or POI exist and possess characteristics that could be complementary. They can be summarised by the nature of their producer and share common characteristics:

- **Authoritative** datasets contain uniform and validated data at a national level.
- **Local** datasets are maintained by local administrations and can display manually validated data, which may be more precise.
- **VGI** datasets are crowdsourced datasets with some spatial inconsistencies but a large variety of interesting information.
- **Commercial** datasets are produced by companies, most of the time selling services, and are usually not fully open.

Such databases can be systematically compared or conflated (which is the process of matching and then merging POI). Table 3 summarises different related works:

**Table 3:** Summary of works related to POI.

General Objective	Location	Attribute	Conflation
Generation or creation of new object		[37]	[38–41]
Analyse/comparison in large dataset	[41–45]		[46]
Historicisation	[41,47]	[39,41,47]	

This summary highlights that the most studied aspect of POI analysis is the comparison of POIs, often between different sources. Both the accuracy of specific attributes and their spatial location are analysed.

POI conflation is also a high-interest subject [46]. provide a state-of-the-art review of methods for conflating geolocated POIs. The authors highlight the lack of reference POI datasets for controlled algorithm testing. The nonexistence of a ready-to-use toolbox or annotated datasets of state-of-the-art POI matching and merging tools forces the authors to reimplement all algorithms themselves. Their algorithms are not available under an open source licence, as are many works mentioned in this section.

The generation of additional information has not been found in the literature. Few works use web mining to enrich POI knowledge; [37] build indexes and provide descriptive semantic information, but this cannot be considered systematic and ordered attribute generation. Such knowledge would have been useful for the model’s enrichment described in Section 3.3.

Historicisation of POI is an explored topic. Works mainly focus on analysing temporal patterns across different POI sources [41], the evolution of particular toponyms [47], or the correlation between POI evolution and urban transformations [39]. Other works, focusing more on activities than POI, deal with the historicisation of geo-historical data, which are heterogeneous, fragmented and incomplete [48].

## 3 Method

Many of the works presented in the state-of-the-art are from distinct fields. No systematic implementation or directly reusable software was available to be integrated into this model. Their knowledge could, however, be useful for modelling realistic building land use. The model introduced in this section presents an original method to generate building land use. As this model was originally developed for France, the description is based on French datasets and resolves the related challenges. It could, therefore, be applied to other countries’ datasets with few adaptations if their datasets meet the minimal requirements.

First, new building objects are modelled with the help of other geographical datasets (Section 3.1). Then, activities are censused, matched and merged (Section 3.2). Finally, the building land use information is enriched with many details (Section 3.3).

### 3.1 Model of a building object

The building model definition used in this work is an ensemble of closed human-made structures, possibly not continuous, which share one or more common spaces. It is first necessary to define the minimal available data that could be used to address this task (Section 3.1.1).

The first step of this modelling task is the generation of **building blocks** (Section 3.1.2). Such buildings are the primitive objects of the model, and each individual building should meet minimal attribute requirements. If they are missing from the original datasets, this model has multiple options to generate them.

The novelty from this work, due to its own building definition, is the creation of **building groups** based on the continuity of their *functional spaces* (Section 3.1.3). To our knowledge, no scientific work is modelling buildings at an individual scale with this functional paradigm, for which the modelling of precise land use would be adequate. It is necessary to group *building blocks* in order to compose common interior spaces. Continuity of functional spaces can also be found in buildings that do not touch, and furthermore are touching other buildings. The main usable knowledge would be given by each *building block*’s administrative status, which is not automatically attached to building’s geographical collections. Postal addresses or cadastral boundaries could be used to fill this gap. Relationships between those objects are complex, particularly for historical dense urban fabrics depicted in Fig 3, where each object can be linked from 0 to n other objects. Postal addresses seem more suitable to define a proper activity, but it is not straightforward to decide the allocation of *building blocks* to addresses. Cadastral parcel geometries do not necessarily match buildings well, a *building block* could be positioned on multiple parcels, as well as multiple *building blocks* could be positioned on a single cadastral parcel. In some cases, links between *building blocks* and addresses exist. If not, a simple process links every *building block* to a postal parcel. All attributes of *building blocks* are finally aggregated with an appropriate aggregation operation.

**Fig 3:**
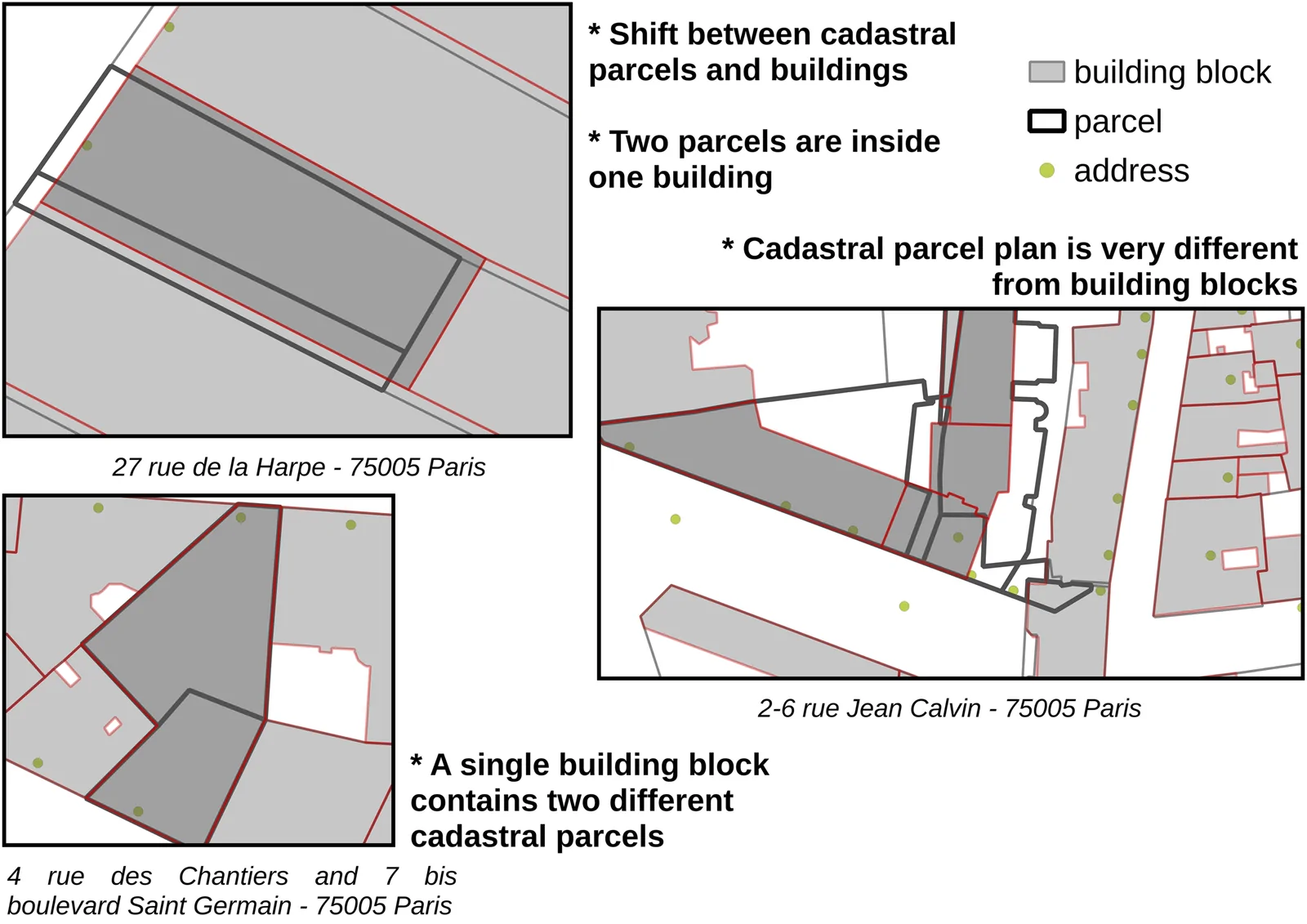
Examples of not trivial topological relations between buildings and parcels in a dense historical urban fabric.

#### 3.1.1 Data availability.

A description of French geographical data is presented in Annex D.1. Multiple building products were identified. The authoritative dataset is considered more precise than the OSM dataset [31] and is available uniformly across the entire country, unlike local databases. This product, named *BD_Topo*®, is therefore selected as the primary building source in this example. Different versions of *BD_Topo*® are available across various time periods (from 2008 to the present) and are released at different intervals (from yearly to quarterly). As building construction is a slow dynamic, interpolating snapshots between *BD_Topo*® releases is unnecessary. However, the product characteristics vary significantly from one version to another, as described in Table 7. The range of attribute availability illustrates the variety of input datasets that may be encountered in other countries. This enables the model to generate missing attributes through various methods. A minimal set of attributes necessary to model *building blocks* is schematised in Fig 4 as follows:

- **2D polygonal geometry of** *building blocks*: the footprint of the *building blocks*.
- **Address(es)**: the administrative addresses attached to the *building block*. It is possible that even a single *building block* is linked to multiple addresses. If not provided, this information can be derived from address datasets or cadastral datasets.
- **Floor area (m^2^)**: the total floor area within the *building block*. This information can be deduced from the number of storeys in the building, which in turn can be inferred from its height. If the latter information is missing, it is possible to compute an estimate based on neighbouring buildings, as described in 3.1.2 (page 10). The floor area could also be deduced using the methods outlined in [26].
- **Number of activities**: The total number of activity premises hosted by the *building block*. In most cases, this value is calculated according to the process described in 3.2 (page 12).
- **Number of housing units (dwellings)**: The total number of housing units per *building block*. If unknown, this attribute is calculated from census information and the floor area of the buildings, as described in 3.1.2 (page 11).

**Fig 4:**
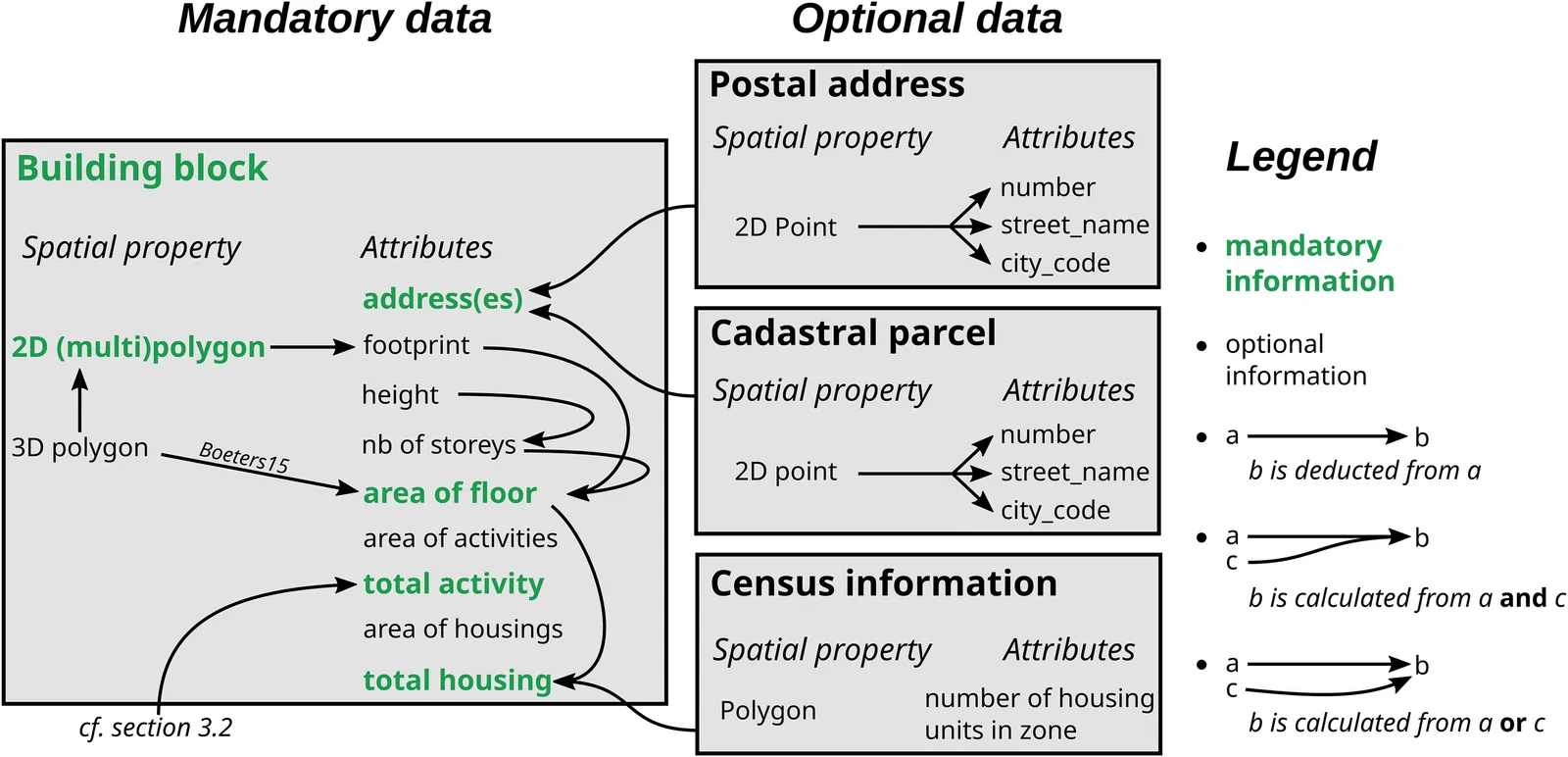
Minimal data requirement for *building blocks* modelling in Building Land Use simulations.

Those mandatory attributes may be directly available from the input data. If so, their generation is unnecessary, or could be applied to fill missing values.

#### 3.1.2 Generation of building block.

In the model of *building blocks*, each component is modelled with different processing, described as follow

**Geometries of building blocks** It can be assumed that the given geometries are correct; therefore, no reconstruction or precision enhancement is handled by this model, and the geometries are retained as they are.

**Height** The height of a building in *BD_Topo*® is calculated between its lowest point above the ground and its gutter. This definition excludes basements, which could be used for commercial purposes. The space under the roofs is also excluded, although it can be usable for attic apartments, which is very common in a Hausmannian typology. It should be possible to take this space into consideration using the 3D methods presented in [26]. The height attribute could be missing. In *BD_Topo*®, this concerns 0.4% of buildings in 2009, 0.9% in 2019, and 5.7% in 2024. Most of the time, this arises from a value initially set to 0 and corrected to reflect the unknown nature of the building height rather than its flatness. It is possible to reconstruct its height by calculating the average height of buildings surrounding the one with the unknown height. Buildings with no residential purpose can be filtered out, as they may benefit from exceptions to local urban planning construction rules and have a height disconnected from their neighbourhood.

**Number of storeys** It can be provided or deduced from the height. Neither systematic LIDAR data nor well qualified and global data are used in this implementation, which could unlock more complex reconstruction algorithms. *BD_Topo*® provides the number of storeys based on tax information, but many values are missing. In *BD_Topo*®, this concerns 54% in 2019 and 49.2% of 2024. In this case, a fixed value of height is set (3 meters), and a number of storeys is deduced by rounding the division of the height.

**Floor area** The number of storeys and the spatial footprint of the building make the calculation of an effective floor area (EFA) possible. Even if a multiple legal definitions exist [29], a sufficiently simple one is set, which allows the systematic calculation of this value, as given in Equation 1.


$$\begin{array}{c} EFA=B_{footprint}*N_{Storey}*C_{reduction} \end{array}$$ (1)

The reduction coefficient ($C_{reduction}$) deducts the area which is unusable (elevator/stair shafts, chimneys, wall surfaces, etc.). It is usually set between 0.7 and 0.85, depending on the definition of the EFA.

**Number of housing units** *BD_Topo*® gives a number of housing units per *building block*, deduced from tax information. Numerous buildings have no such value. In *BD_Topo*®, this concerns 54% of buildings in 2019 and 49.2% in 2024. When only buildings with the *residential* tag in their *use* attribute are selected, it drops to 4% in 2019 and 5.9% in 2024. 0.1% in 2019 and 1.6% in 2024 have no height as well. In this case, a process which requires zoning census data about the number of housing units is applied. This process distributes missing housing units in the building in proportion to their floor area. Pseudocode 1 describes this algorithm in detail.

#### 3.1.3 Aggregation of building blocks into building groups.

The definition of *building block* objects is based on a purely morphological criterion. Grouping individual *building blocks* to form *building groups* ensures functional continuity between the hosted *functional spaces*. To do so, a **reference** must be attached to each *building block*. Addresses are preferred, with an order depending on their form: preferably those with links connecting them to one or more buildings, those representing an entrance, at last, addresses tied to a cadastral parcel. Cadastral parcels themselves could then be used to attach a reference. Each *building block* needs to be attached to at least one *reference*. If multiple *building blocks* share a common *reference*, they are merged into a *building group*.

In order to be adaptable to multiple configurations of data availability, multiple ways of linking at least one address or parcel to *building blocks* are possible. Fig 5 describes this workflow based on available datasets, pseudocode 2 details the algorithm, and Fig 6 schematizes an example.

**Fig 5:**
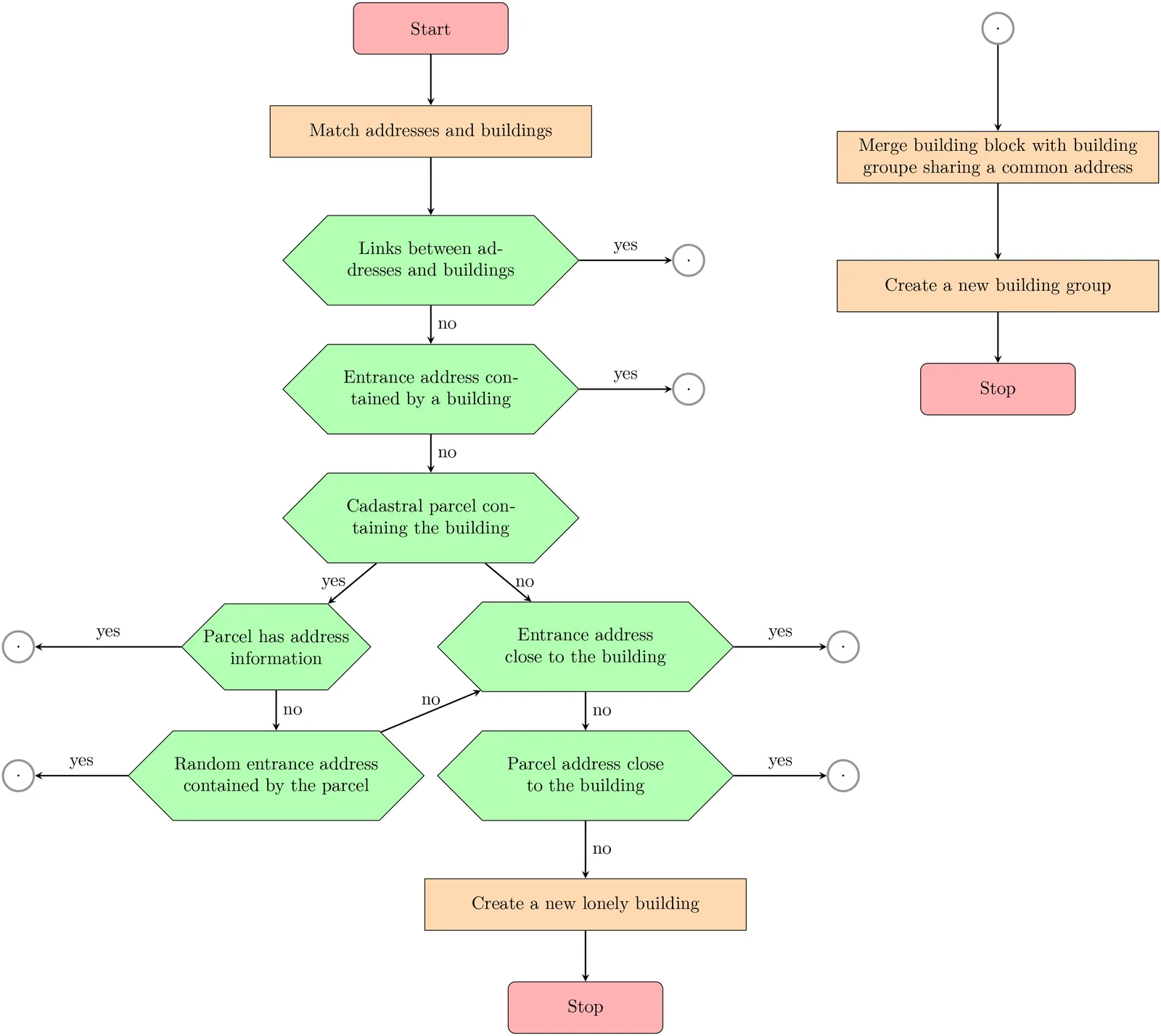
Process of grouping *building block* into *building group* using various references.

**Fig 6:**
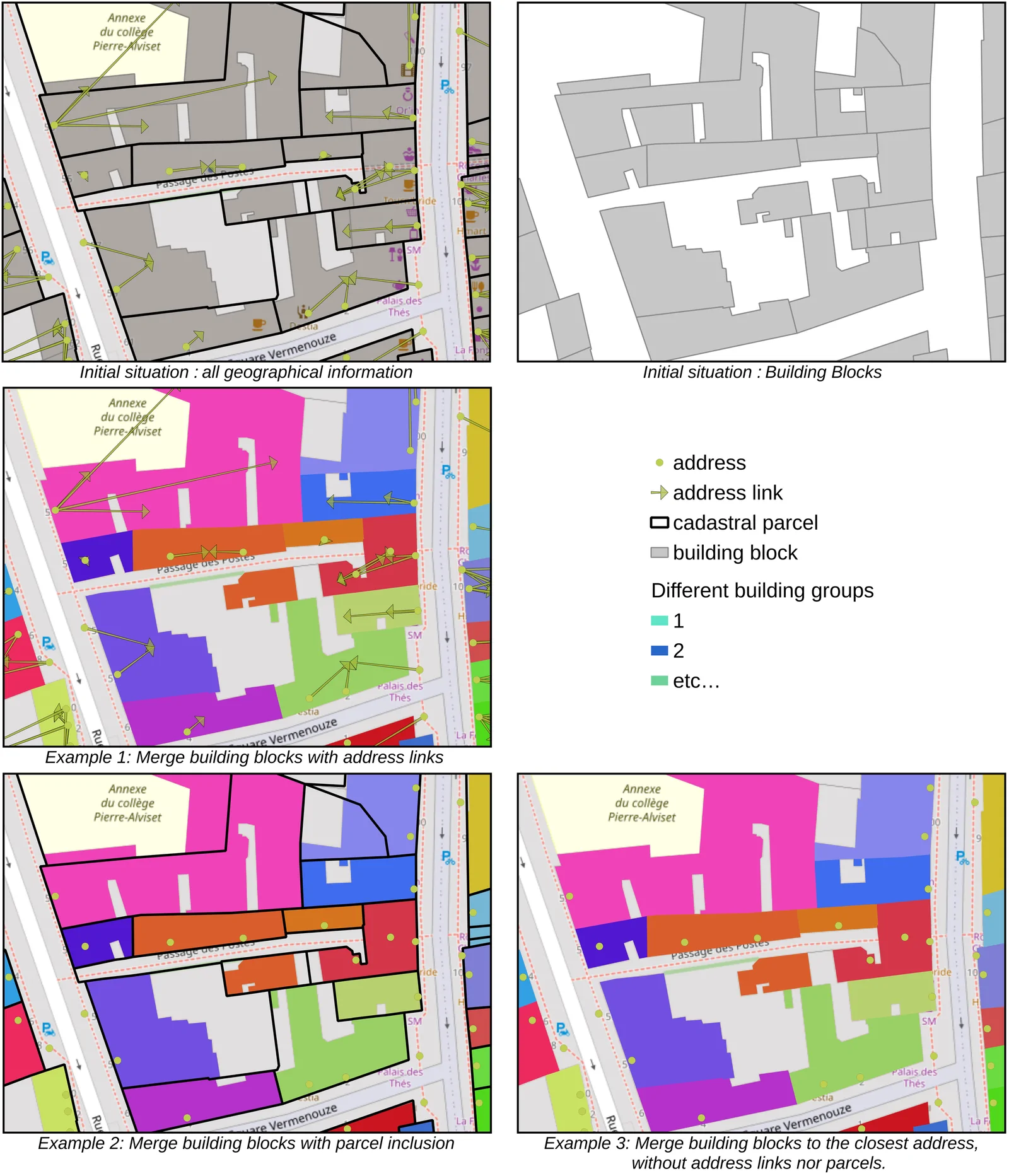
Process of grouping *building block* into *building group* on an urban block at the north of AgroParisTech – 75005 Paris.

When *building blocks* are merged, some quantitative attributes (EFA, number of housing units) are summed up, while others (height, number of storeys) are appended.

There are opportunities to improve the attachment of a building to a parcel by considering the relationship with the street instead of the distance to the address point. Other data could also be used, such as activity-related data.

### 3.2 Model of the activities

Building groups have been modelled to allow continuity of their interiors. The number of housing units within them is known. The model must now collect and conflate activities in order to sketch the composition of a well-described mixed use. The corresponding state-of-the-art Section 2.3 highlights that, although many works have undertaken a similar task, no general open-source projects are available. This section then presents an original open-source activity conflation model.

Activities modelled in this work are necessarily situated inside a building. In many cases, they are relatively independent of the building characteristics. They rely on different project typologies*, are represented by various jurisdictional statuses and do not necessarily imply the same occupational status within a building. They are also prone to frequent changes.

Multiple data sources containing different characteristics about indoor activities are available: authoritative censuses, thematic selections, local databases, customer-review aggregators and collaborative cartographic databases. Processes of activity conflation can be deployed to combine multiple data sources for generating the most exhaustive census of activities inside each building and to enrich this representation with complementary information.

A historicisation process is also important to ensure a realistic representation across different dates and to apply modifications to datasets that are not up-to-date.

An indoor space dedicated to hosting a *functional space* is called **premises** *. Every declared activity does not necessarily occupy a premises (see Section 3.3.1). The exhaustiveness of certain activity datasets can lead to modelling multiple activities and therefore makes it not straightforward to determine the composition of building interiors. Nevertheless, the conflation with other datasets could be used to infer the importance of each activity.

Based on those specifications, an original pipeline has been designed. It systematically builds an exhaustive census of activities located inside buildings. It takes advantage of the diversity of available data sources and is flexible in terms of dataset quantity and quality. To illustrate its potential, the pipeline is applied to complex and freely available French datasets, but it could be used elsewhere with similar or simpler datasets.

#### 3.2.1 Data availability.

The activity merging process can be used with a single dataset or with many. This section is exemplified using a few prominent French activity-related databases. As a focus on free and open data is endorsed in this work, no commercial data are used. French activity data are fully described in Annex D.2 and can be summarised as follows:

- **SIRENE**: an authoritative dataset gathering every declared activity, whether currently open or closed, along with many descriptive attributes and the dates of every administrative changes. This dataset is *historicised* and *exhaustive*.
- **BPE**: an authoritative dataset gathering every piece of public equipment. It combines the *SIRENE* dataset with data from many official French institutions. This dataset is *exhaustive* and results from *manual processing*.
- **OpenStreetMap**: a VGI dataset with an extensive data schema and rapid evolution. This dataset can be *historicised*.
- **APUR**: a local dataset covering shops, leisure activities and social facilities in the Paris area. This dataset results from *manual processing* and will be used to demonstrate the advantages of integrating local and precise datasets.

#### 3.2.2 Typology of activities.

All databases are designed with different typologies*, providing a proper classification of activities using original types. According to [44], the automatic use of the WordNet database to match types is not precise and yields too many correspondences. The methodology of building a single general *typology*, named *common typology*, adapted to each project is preferred. The *common typology* used in this project is described in Annex D.2.1. For each class of the different *project typologies*, it is necessary to manually assign a correspondence with the *common typology*. Matching a fixed typology with OSM is known to be a tedious task [41] because these adaptive and flexible typologies could contain many different classes. Additionally, some classes are incorrectly defined, or an activity may have multiple types. Automatic string-matching patterns could be used to preprocess the manual labelling. The *project typologies* remain important information for providing more precise details about activities and are used in the merging process described in the next section.

#### 3.2.3 Historicisation.

The evolution of OSM data is constant and unstructured. Changes in this database are made by a spatially and temporally inconsistent base of users. When a modification is registered, it is hard to determine whether an activity has just opened, whether some of its characteristics have evolved, or whether it has simply been registered in the database but existed for a long time. Registered objects may not exist and still be present in this database. The *SIRENE* database contains information of the opening and closing of each establishment, along with every change in names, types, number of employees or jurisdictional statuses. This feature makes it possible for this model to generate a set of activities precisely for any desired date. By matching activities with such historicised data, it is possible to assess whether each activity’s characteristics have changed, whether the activity has closed or is not yet open, for any given date covered by the historicisation. It is then possible to update a dataset to a particular date, whether in the past or the future. However, it is not possible to recreate outdated activities that have been closed and removed from legacy databases.

This very detailed historicisation might be particular to the SIRENE dataset. In a context without such data available, it might be possible to characterise each change in OSM activities and attempt to historicise them.

#### 3.2.4 Activity modelling.

This section now presents the process for the systematic conflation from one-to-many activity datasets in order to generate an exhaustive set of indoor activities at a precise date. It reuses classical POI conflation approaches, involving the calculation of a similarity score, followed by different kinds of merging for pairs with a score higher than a given threshold. The first step *relocates* isolated activities to the closest or corresponding building. Then, if one dataset is historicised and others are not up-to-date and need to be updated, it is possible to *update* chosen fields of the latter dataset using activity matching. If a dataset allows an activity to have multiple register entries, it is possible to perform a *self conflation* of activities from a single dataset. Then, every activity from each dataset is assumed to exist, which implies that all of those activities will be included in the final activity collection. Finally, the algorithm performs the *conflation* of each dataset feature with every other feature from other datasets. Fig 7 schematises this process and gives an example of activity modelling from four different input datasets. Every process and task designed for this purpose is now presented.

**Fig 7:**
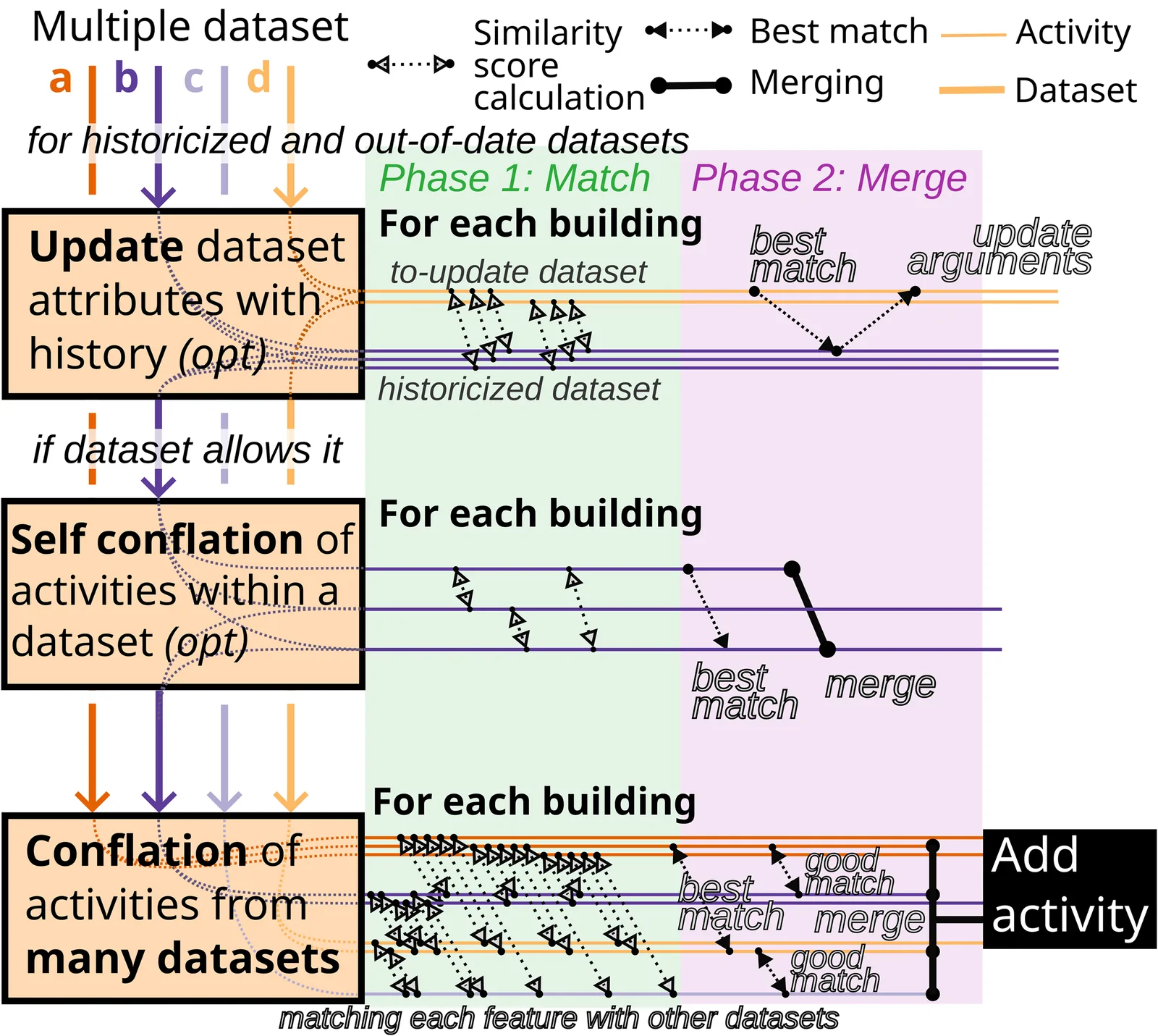
Workflow for the conflation of multiple activity datasets contained in a building. Only datasets **b** and **d** are part of the *historicised update* task, and only dataset **b** is self conflated.

**Relocate activity** In [44], a quality analysis comparing authoritative and VGI POI datasets measured that VGI points are less than 70 meters away from the reference point, with a mean distance between 15 and 25 meters. POI are modelled with a punctual geometry, which could represent the entrance of the building, its centre, or simply pin their position randomly in or near the building. No precision is given regarding whether the POI is included in the building or not. Due to this information, the first step of our processing chain corrects every activity geometries following these steps:

1. Match the addresses of activities with addresses from the surrounding *building groups*. If the activity is not inside the matched building, the activity is relocated randomly into the building’s geometry.

If no address information can be deduced from the activity or no building is matched, next steps are applied:

2. If the activity is inside a building, its position inside this building is kept.
3. If the activity is not inside a building and is close to one, the activity is relocated inside the closest building.
4. Ultimately, the activity is considered an outside activity and excluded from the final dataset.

After this process, every activity is now located within a building (Fig 8). Pseudocode 3 in Annex E describes this process.

**Fig 8:**
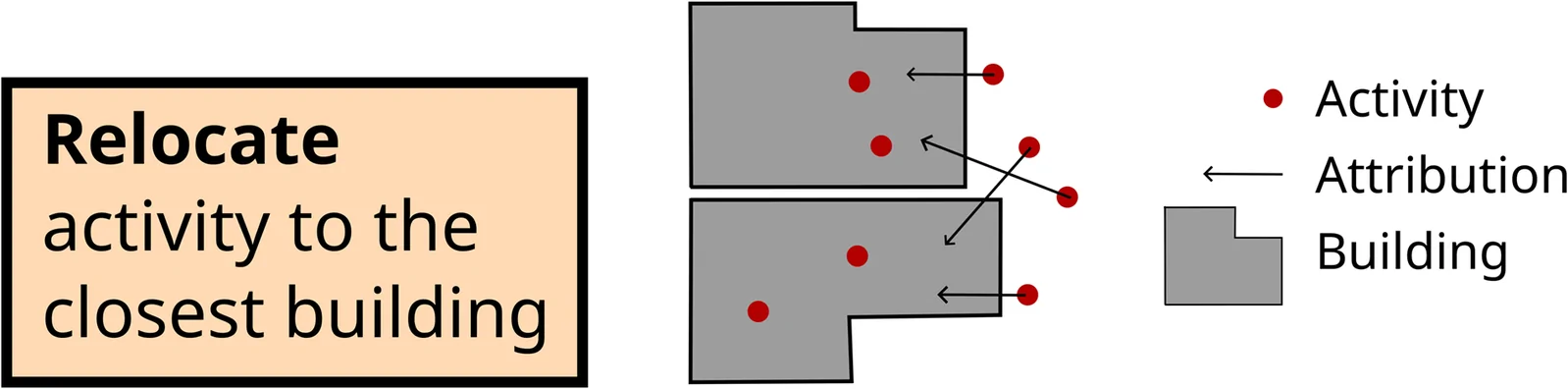
Relocation of activities outside buildings.

**Matching activities** As frequently done in the POI conflation literature [46], a *similarity score* expresses the resemblance between two activities. This is based on two kinds of attributes which imply distinct calculations in this model:

- **Type similarity** is calculated with both activity typologies, namely *common typology* and *project typology*. Every type from the *common typology* must be exactly equal. As there are multiple depths in this typology, this equality condition stops at the second level. If this condition is not met, the match is considered impossible. Typology names from each *project typology* are compared using the pre-trained multilingual sentence-similarity BERT model described in [49]. This model also produces comparison of types written in different languages, which is useful to compare English VGI to French typologies.
- **Name similarity** is calculated only if both activity names are available. String similarity indicators listed in [46] are used to evaluate the similarity of activity names and propose matches. Distances between words are the simplest indicators to calculate. Two indicators of string similarity are chosen and the **maximum** value of such indicators is kept:
  - The *Levenshtein ratio* indicator calculates the cost of changing characters in order to transform string A into B.
  - The *longest common substring* (LCS) ratio counts how many characters compose the longest common sequence of characters in the two compared strings.
  - All those indicators are normalised by the length of the input string using the following formula, in order to eliminate cases where very small names would have a great LCS ratio:

$$\begin{array}{c} \left\{ \begin{array}{cc} \frac{length\_str\_1+length\_str\_2}{2} & \;\text{if }length\_str\_1\leq 3\text{ OR }length\_str\_2\leq 3\\ \min length\_str\_1,length\_str\_2 & \;\text{otherwise} \end{array}\right. \end{array}$$

Sometimes, activities have multiple names (as in the SIRENE dataset or in any self-conflated dataset) or types (OSM data). In such cases, similarities are calculated for every name or type and the highest score is kept. Each string of characters is *preprocessed* in order to standardise it as much as possible. For *type matching*, similarities based on raw and preprocessed types are calculated and the maximum value is kept. The preprocessing steps are:

- Lowercase and replace special characters with corresponding ASCII characters.
- Remove words that do not produce potential differentiation (legal unit description, country name, name of the street where activities are located). *Only on name matching*.
- Remove phrases and words with a size equal to or less than 2 characters.
- Replace synonyms with the most common word (for example, *practice*, *cabinet* and *surgery* become *office*) (*Only on type matching*).
- In certain cases, replace very formal types with more common expressions (for example, *retail shop in meat and meat products in specialised stores* is replaced by *butchery and delicatessen*) (*Only on type matching*).

The *similarity threshold* is set to 0.5 by default. If name information is present in both activities, the mean of the *name* and *type similarity* scores is calculated. Otherwise, the *type similarity score* is evaluated alone. This setting implies that the matching will be validated if names exactly match, even if the original type names have no resemblance, which does not indicate they differ because they can be registered under very different *project types*. On the other hand, if the original types are exactly the same, the match is also evaluated as valid even if their names differ. Pseudocode 4 in Annex E describes those processes.

**Merging activities** The merging process is adapted to the specificities of datasets. The manner of merging each kind of attribute from distinct datasets must be decided beforehand. It is possible to *concatenate* values, *erase* values in favour of the most accurate dataset, or *add* numeric fields. Those operations can vary according to the features that are merged: merging between multiple sources for updating datasets, self conflating activities within a single dataset, or conflating multiple datasets (see next paragraphs). Specific attributes and their concatenation methods for the databases used in the French context are listed in Table 9 in Annex D.2, along with attribute descriptions.

**Update not-up-to-date datasets** This is the first process of the conflation processing chain schematised in Fig 7. Frequently, an interesting dataset is not available at the targeted date of data generation $t_{G}$, either because the data producer has not published a recent release yet, the timespan between two dataset releases is too high, or the dataset did not exist at that date. If an historicised activity dataset is available, it is possible to update particular fields. This processing chain matches out-of-date activities to historicised activities, rewound to the release date $t_{R}$. The *similarity score* between each activity is calculated, as described in the previous paragraph. If a modification has occurred in the historicised activity between $t_{R}$ and $t_{G}$, this modification is reported to the updated dataset. In the French application, the SIRENE dataset has historicised *opening status*, *names*, and *type* fields. These are used to update the OSM dataset (as the downloaded database is the latest and $t_{G}$ might be in the past) or other datasets that have a long release cycle. It is possible that an activity is removed from the updated dataset because it is closed or not yet open at $t_{G}$. Pseudocode 5 in Annex E describes this process.

**Self conflate activities within a single dataset** It is possible that multiple entries of a dataset belong to the same activity. The *near duplication* problem [37] is a well-known challenge in POI conflation and could appear within a single dataset. An adjacent problem arises from the fact that multiple declared activities could share the same premises and workers but be represented by different legal entities. They could have different types, and all other attributes, such as the number of employees, would vary. It is also possible that different establishments of the same activity are declared in adjacent buildings and that those buildings are grouped together by the grouping process.

A self conflation process is applied to every dataset in which such problems are detected. For the French application, only SIRENE data is known for containing multiple declarations. A similarity score is calculated for all pairs of activities located in the same *building group*. As it is expected that activity types vary, the score only considers *name matching* similarity. A matrix with similarity scores is built, and every activity whose similarity value is higher than the *similarity threshold* is merged. An unlimited number of activities can be matched, so multiple values can be matched even if they do not match with every other value, as exemplified in Fig 9. Pseudocode 6 in Annex E describes this process.

**Fig 9:**
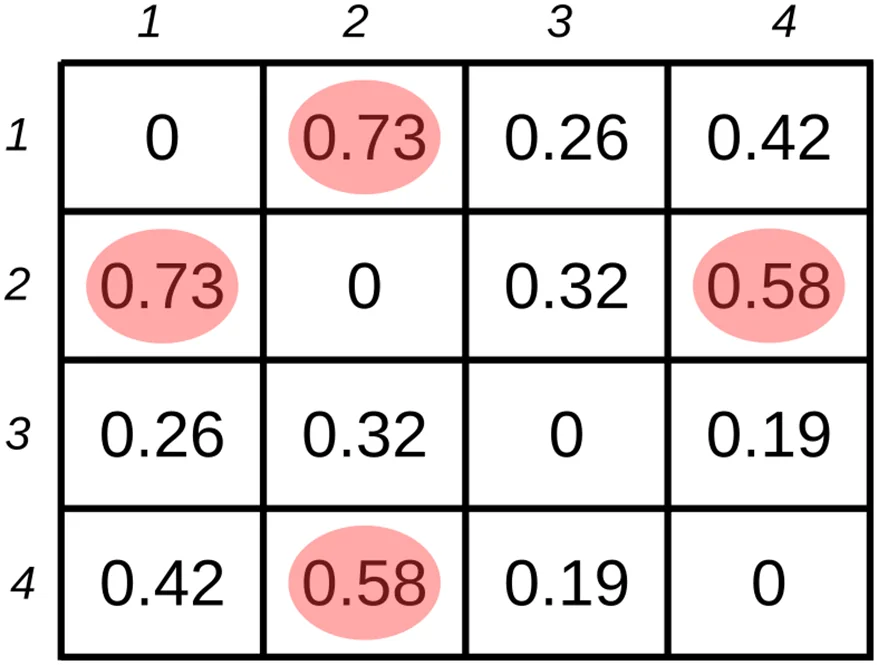
Example of a matrix of self conflation. *Activity 1* matches *Activity 2*, and *Activity 2* matches *Activity 1* and *4*. *Activities 1, 2*, and *4* are then merged.

**Conflate activities from multiple datasets** At this step of the processing chain, each entry of the datasets is considered to effectively represent an existing activity. Therefore, it is impossible to match two activities from the same dataset. A *similarity score* is calculated for each activity with activities from every other dataset. The matching pairs are sorted by the highest *similarity score*. Proceeding with the highest matching pair, similarities with activities from every other dataset are retrieved in order to add their highest similarity values to the pair, provided they exceed the threshold. Those other dataset activities will not be available for the next merging iterations. Once the threshold is reached, every unmatched activity is added to the collection as a singleton. Pseudocode 7 in Annex E describes this process.

### 3.3 Enrichment of building land use

An original building definition has first been set and implemented in order to ensure the continuity of interiors. The number of housing units within each building is also collected or generated. An automated census and conflation model has been designed to create an exhaustive collection of activities contained in each building. It is now necessary to articulate each characteristic with its activities and housing units in order to enrich the building land use with useful and descriptive information. As we lack computing time and expertise to generate a 3D topological composition of rooms and activities for a large zone, a textual representation, using an extensive set of attributes, would be sufficient to describe how the interior of a building is composed. However, this could be a first step towards systematic procedural generation of building interior settings.

Each attribute is set using various processes, sometimes involving other building elements or related to activity type. The first step decides if activities possess a premises (3.3.1). Optionally, other attributes can be defined, for example whether an activity is open to the public (3.3.2) or what its opening hours are (3.3.3). The final step sets a floor area for each activity’s premises and housing units (3.3.4).

#### 3.3.1 Presence of premises.

As the activity census could collect a significant amount of varied data, no information about the presence of a dedicated premises is available. Such information could have been found in tax-related data, but its use is too revealing to be available at a microscopic level and is not open. Self-employment, which has clearly increased in most countries in the past few years [50], is an activity without a specific premises for instance. Other activities, such as associations or real estate cooperatives, can also be purely administratively attached to a location and might not require anything other than a name on a mailbox. A special process is then created to determine whether a modelled activity occupies a premises, taking into consideration the multiple characteristics modelled previously.

For each typology class, a probability of having a premises is set. This could be set at the *project typology* scale. By default, the value defined at the *common typology* would be used. Many activities always have one (restaurants, doctors, etc.), others never occupy a premises (food delivery, small taxi companies, microcompanies, etc.), and some depend on the case (tertiary sector offices, personal care, etc.). This probability is then transformed into a **score** by adding bonuses or penalties. A *Bernoulli trial* is then performed with the final score as the parameter to determine whether the activity occupies a premises or not. If not, no other attributes are set. Bonuses and penalties are defined according to the following information:

- **presence in other datasets**: If the activity hasn’t been matched with any other from the datasets, a penalty is applied to the score. Otherwise, a bonus is added; its value is relative to the number of matched datasets.
- **jurisdictional status**: Few particular statuses (for example, self-employed activities or simple owner-managed real estate properties) lower the premises score.
- **presence of values**: If particular attributes, such as the number of employees or opening hours, are set, this serves as a strong indication that the activity is open to the public.
- **average floor area per premises in the building**: This is the final step of the attribution, performed after the Bernoulli trial on premises presence. When the average size of all activities and housing units in the building is below a fixed threshold (10 square meters by default), it is likely that too many premises were allocated. A penalty is iteratively applied to every score until this condition is satisfied.

Pseudocode 8 in Annex E describes this process and the values used for bonuses and penalties.

#### 3.3.2 Opening to the public.

In order to reconstruct the inside of building or to run multi-agent simulation, it is a matter of interest to know if an activity accepts the visitors, and what is the frequency of people coming in. The fact that an activity could be open to the public depends on the presence of a premises and the type of the activity. A probability based on the *project type*, and if missing, its corresponding *common type*, is set. This probability is completed with a bonus or penalty concerning the *presence in other datasets* as described in the previous paragraph. A new *Bernoulli trial* is performed with this new parameter to determine whether the activity is open to the public.

An *accessibility indicator* is finally set to express the amount of visitors that activities would receive. This indicator is calculated depending on the activity type (for example, a garage would receive fewer customers than a bakery) and attributes which can reflect the importance of the activity (number of employees, capacity, etc.)

#### 3.3.3 Opening hours.

Opening hours should be assigned to all activities open to the public. As they could change rapidly, this information should be provided by VGI or commercial data sources that might update more frequently. In our case, OSM data often records manually collected opening hours in a well-described and automatable format (see Table 9 for attribute coverage). This format is presented in this OSM wiki resource: https://s.42l.fr/osmOH When an OSM activity is merged with other activities, this information is retained if provided. If not, synthetic opening hours are assigned based on the activity type. These values could be varied to avoid uniform opening hours. Currently, variations are simple (slight shifts of opening time, closing time, or opening days, and opening at night or on weekends if the *common type* permits it), but it would be valuable to learn from manually recorded opening hours to provide more realistic synthetic values, depending on the activity type and location.

#### 3.3.4 Area attribution.

A single building can be divided into multiple uses, which results in the impossibility of knowing *a priori* how much space is dedicated to which activity. Once activity premises are identified and detailed, it becomes possible to assign an area to each one of them, along with housing units, proportional to their building’s floor area. This problem has the following characteristics:

- Each building has a known floor area value, which is supposed to exclude unusable surfaces (see Section 3.1.2). Optionally, a value for the total square meters for commercial or housing purposes could be provided by tax services.
- A known number of housing units and activities with a premises within each building.
- The sum of activities’ and housing units’ areas must equal the building’s floor area.
- French national statistics provide the number of principal, secondary, and vacant housing units per statistical aggregate* and the number of principal housing units discretised into seven classes of area size.

As there are many unknowns in this problem, making it impossible to determine if any solution exists, the following simple method is chosen:

1. **Preset an area for every activity.** For every activity, a premises area is outlined. This area can be viewed as the minimal value needed to host the activity. This information is estimated using the activity’s and building’s attributes. The following information is used if present:
  (a) **Specific attribute information from the dataset**: For example, each activity in the *local* APUR dataset possesses manually set area bounds.
  (b) **Number of people in the activity**: The number of workers and the capacity of the activity are used, if set, with 10 square meters per individual.
  (c) **Attendance information**: This indicator is calculated according to the type and size of the activity. A fixed value is preset for each level of attendance.
  (d) **Building floor area**: This process ensures that the sum of such minimal activity areas is below the available floor area in the building.
  This step is presented in detail in pseudocode 9 in Annex E.
2. **Attribution of area to each housing unit.** For all statistical aggregates, a probability vector is calculated to determine the likelihood that a random housing unit belongs to a specific area class. Each housing unit has then an area class attributed randomly via a weighted random categorical sampling. As seven ($\leq 30m^{2},]30;40]m^{2},]40;60]m^{2},]60;80]m^{2},]80;100]m^{2},]100;120]m^{2}$, > 120*m*^2^) classes are recorded in the census, highly diverse configurations can be generated for the set of housing units in a building. The same process is duplicated to assign a status of principal, secondary, or vacant housing unit, but this does not concern area attribution.
3. **Evaluation of the generated areas.** The sum of the preset floor area of activities and the floor area of housing units must be less than or equal to the building’s total floor area. If the first value significantly exceeds the second, Step 2 is repeated. After 500 failed iterations, it is considered that no solution consistent with the statistical aggregate can satisfy this problem. Housing units’ area are then set to the mean of area per housing unit.
4. **Maximise the size of housing units.** This method tends to favour smaller housing units, as they have a higher probability of complying with the area constraint. Consequently, the area of housing units is maximised by replicating the area attribution process and retaining the solution with the highest sum of housing unit areas.
5. **Set a final value for activities.** As the building area not used for housing units must be allocated to activities, the available space within each building is calculated. This area is distributed among activity premises on a pro rata basis according to the initial preset areas.

Fig 10 illustrates this process, and pseudocode 10 in Annex E describes it in detail.

**Fig 10:**
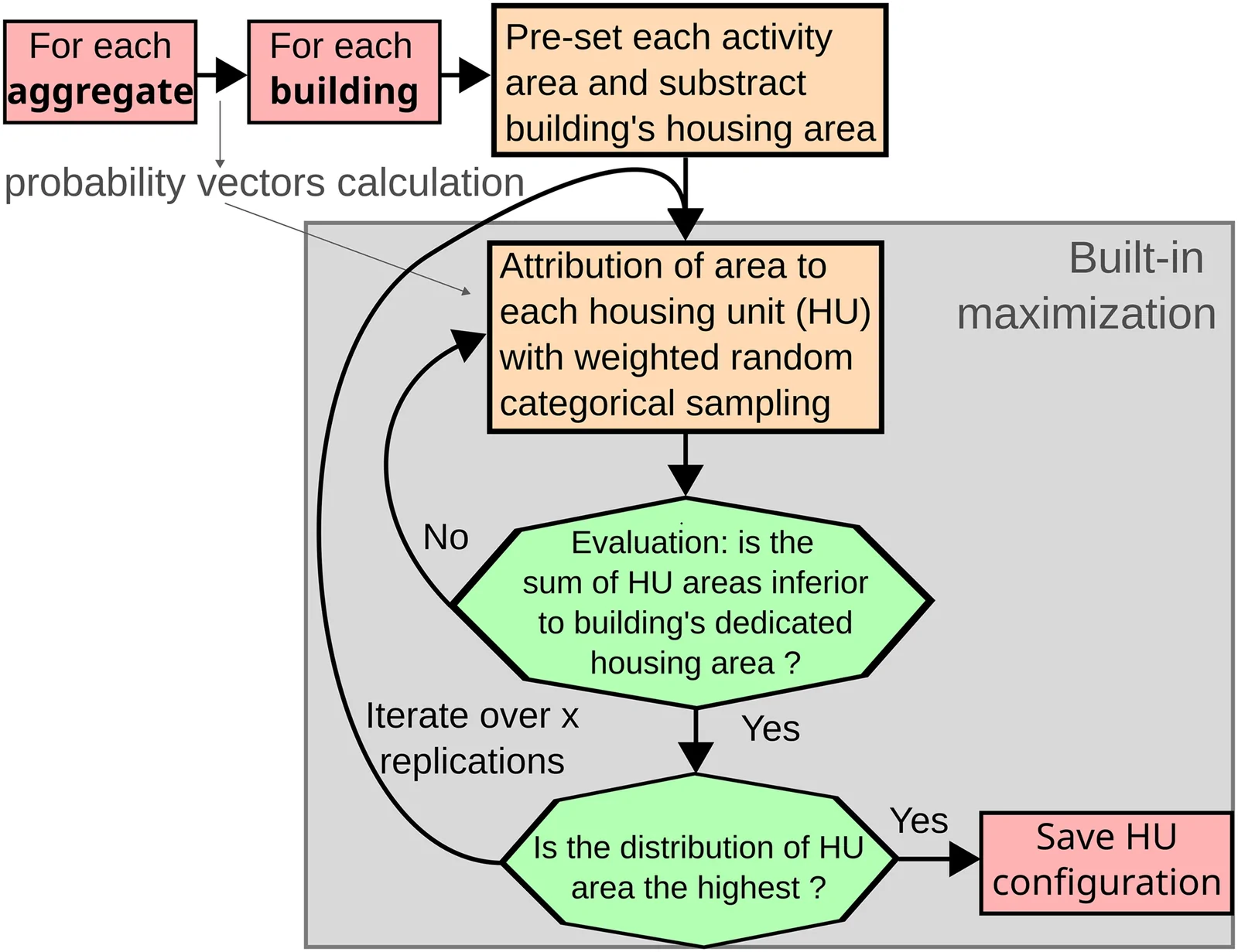
Schema of the area attribution for housing.

## 4 Implementation, results and validation

The implementation of the model described in the previous section is programmed with the Java programming language. It is released under the AGPL v3, a free and open source licence, and the source code can be found at https://gitlab.inria.fr/ici/ici_buildingactivities. Technical documentation is also available at https://s.42l.fr/docICI. As this project is still under active development, version 0.1 was used for the present work.

This section presents independent processing chains designed to evaluate or validate the model tasks introduced in the preceding section. Depending on the chain, simulated zones concern either the whole France, the city of Paris (which represents the most complex urban fabric of France), three arrondissements of Paris, or three sectors within those arrondissements. The 5th, 11th and 19th arrondissements of Paris were selected for their diversity, as illustrated in Fig 11.

**Fig 11:**
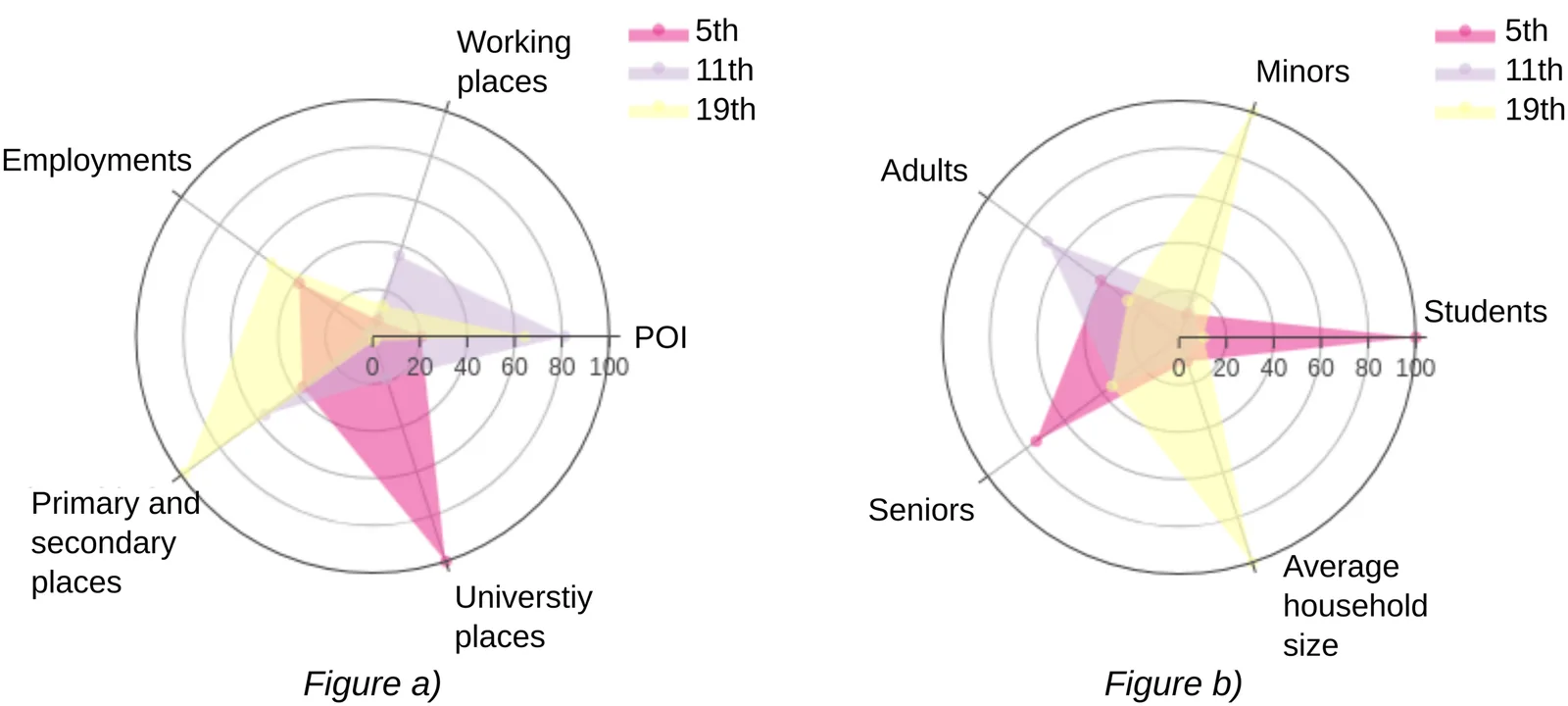
Characteristics of the three example arrondissements: figure *a)* represents urban characteristics, and figure *b*) shows the profile of the population living in such arrondissement, normalised by the total population. These charts are taken from the ICI project, using data from an earlier version than the one presented in this paper. All other charts can be accessed at https://s.42l.fr/spiderCharts.

First, the three tasks of the workflow are evaluated: the building object model (Section 4.1), the activity model (Section 4.2) and the enrichment of building land use, particularly the area attribution (Section 4.3). Validations with ground truth data are then presented (Section 4.4).

### 4.1 Modelling building objects: validation

The dedicated model of a building object has been presented in 3.1. A few processes now analyse the validity of the results or the sensitivity introduced by the diversity of input data. All processes are deterministic, so there is no need to replicate any runs.

#### 4.1.1 Number of housing units reconstruction.

The reconstruction method for the number of housing units, presented in 3.1.2 on page 11, is first analysed. The generation is performed on every individual *building block* in the city. The analysis consists of the comparison between the original values and the newly reconstructed housing-unit counts for each building block (Fig 12). This chain is applied to the *BD_Topo*® of 2019 for Paris, a city in which the majority of buildings are collective structures containing more than one housing unit. The code for this processing chain can be found at https://s.42l.fr/HU.

**Fig 12:**
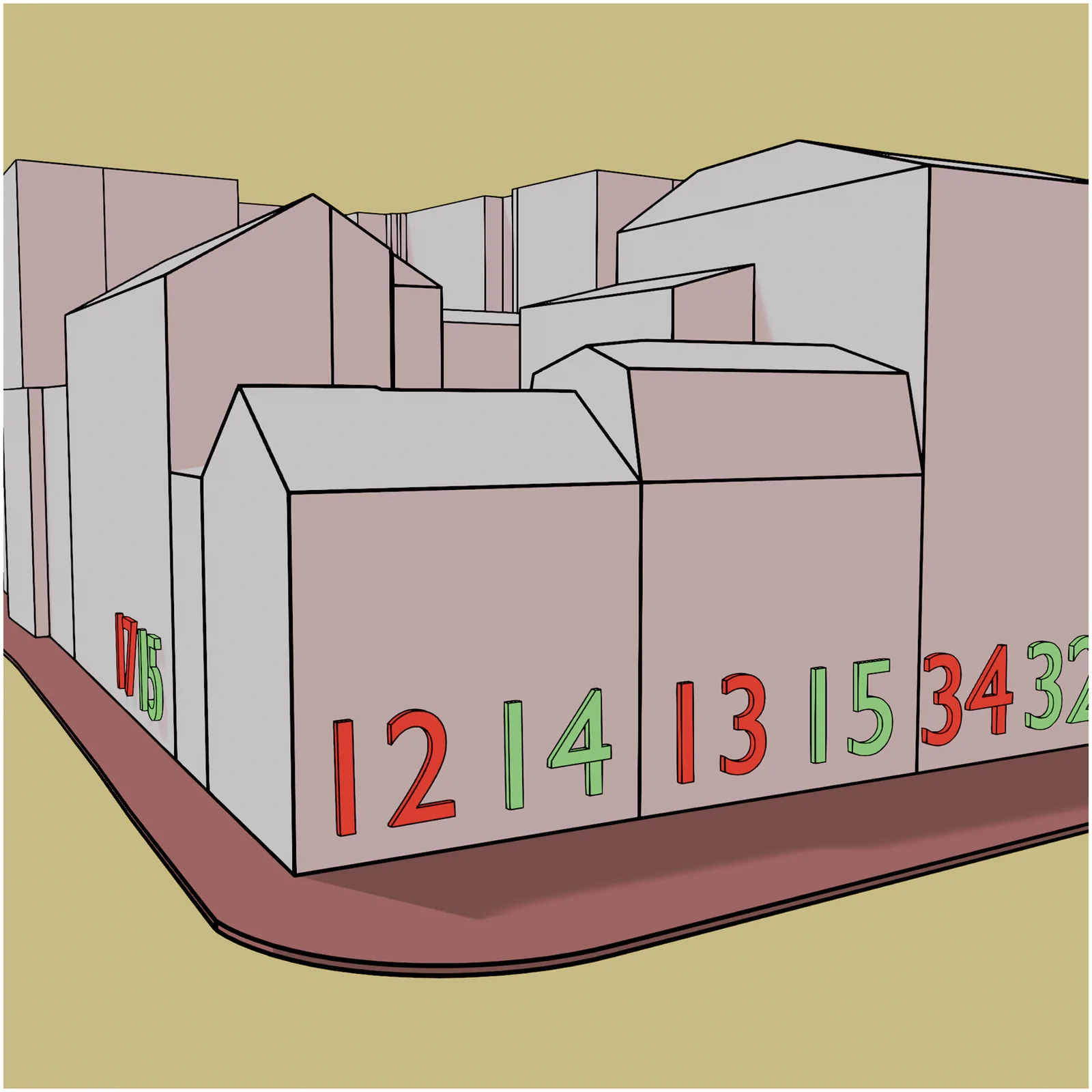
Example of comparison between housing units derived from data (green example) and those obtained from the reconstruction simulation (red example).

Several options can be activated.

As some housing unit values are missing in the original dataset, it is possible to filter out such buildings or to fill the missing values. This processing uses a method similar to the one studied but with a different number of buildings and housing units to be allocated.

It is also possible to select buildings relatively to their uses, by filtering out every building that doesn’t have a *residential* tag. The combination of both options is tested.

Table 4 shows descriptive statistics about the absolute difference of housing units for each of the 118 171 and 82 810 buildings analysed, with and without the filling of missing values. For all cases, the median absolute differences are largely below the median number of housing units per building. The mean difference is higher, owing to a few extremely high values. This occurs because housing units have been generated inside very large buildings that originally contained no or only a few housing units, which were sometimes not correctly tagged or completed.

**Table 4:** Absolute difference of housing units in simulation generated and real data for each building of Paris.

fill missing values	filter on use	1^st^ quantile	median	mean	3^rd^ quantile	max	sum
Housing unit per building							
no	/	2	11	14.19	18	1 147	1 174 947
yes	/	0	6	11.79	15	1 147	1 392 996
Absolute difference of housing units generated and real data per building							
no	yes	1	3	7.138	7	1 161	591 133
no	no	1	4	7.909	8	926	654 981
yes	yes	0	2	5.639	6	645	666 350
yes	no	1	3	7.46	8	902	881 593

Filling the missing values logically results in a greater number of housing units and consequently more errors in total. However, those errors are significantly smaller for each building. Filtering on building use is logically beneficial, particularly when missing values are filled.

Even if the generated housing units do not exactly imitate the data, the error is often very small. This method satisfactorily reproduces and fills the missing information. Filtering on building use should be employed when it is possible.

#### 4.1.2 Building group formation.

As explained in Section 3.1.3 on page 11, different input datasets can be used to group *building blocks*. Different grouping methods are applied, and this processing chain compares such methods. Even if the official links between buildings and addresses should model groups that are linked in some way, it is impossible to state that these links always represent functional continuity. The aim of this chain is therefore strictly to compare different grouping methods, and no absolute validity can be inferred from it (Fig 13).

**Fig 13:**
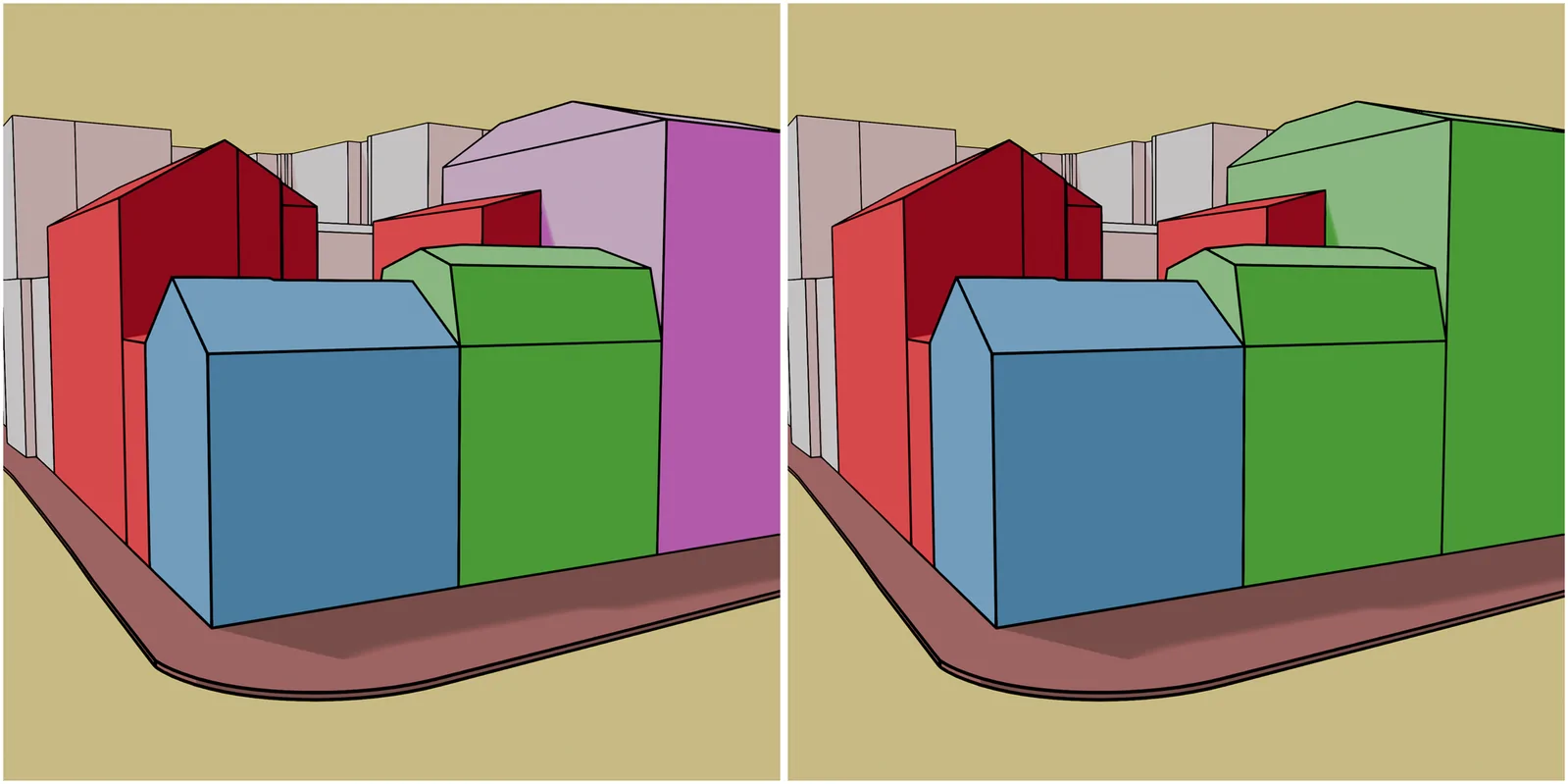
Example of comparison between *Building Groups* constructed with links (right) or parcels and addresses (left).

An input dataset containing only addresses and parcels generates a first collection of *building groups*, while a method that uses official links between addresses and buildings generates a second collection. For each *building block* used in the group creation, this processing compares whether the *building block* belongs to the same group in both collections. Four different cases are counted:

1. If both groups containing the *building block* are identical, the current *building block* is counted as **same group**.
2. If both groups containing the *building block* do not contain any other *building blocks*, they are counted as **singleton**, because no groups have been formed (hence the classification is correct).
3. If both groups containing the *building block* share some *building blocks* but are not exactly the same, current building block is counted as **incomplete**.
4. If both groups containing the *building block* are completely different, it is counted as **different**.

Two measurements are analysed: the number of *building blocks* and their floor area.

This processing chain is applied on the 2019 *BD_Topo*® for the whole of France. The code for this processing chain can be found at https://s.42l.fr/BGcomp, and the raw results can be accessed at https://s.42l.fr/BGcompRes. Results for Paris are presented in Fig 14, while other types of French city typologies are shown in Annex 36.

**Fig 14:**
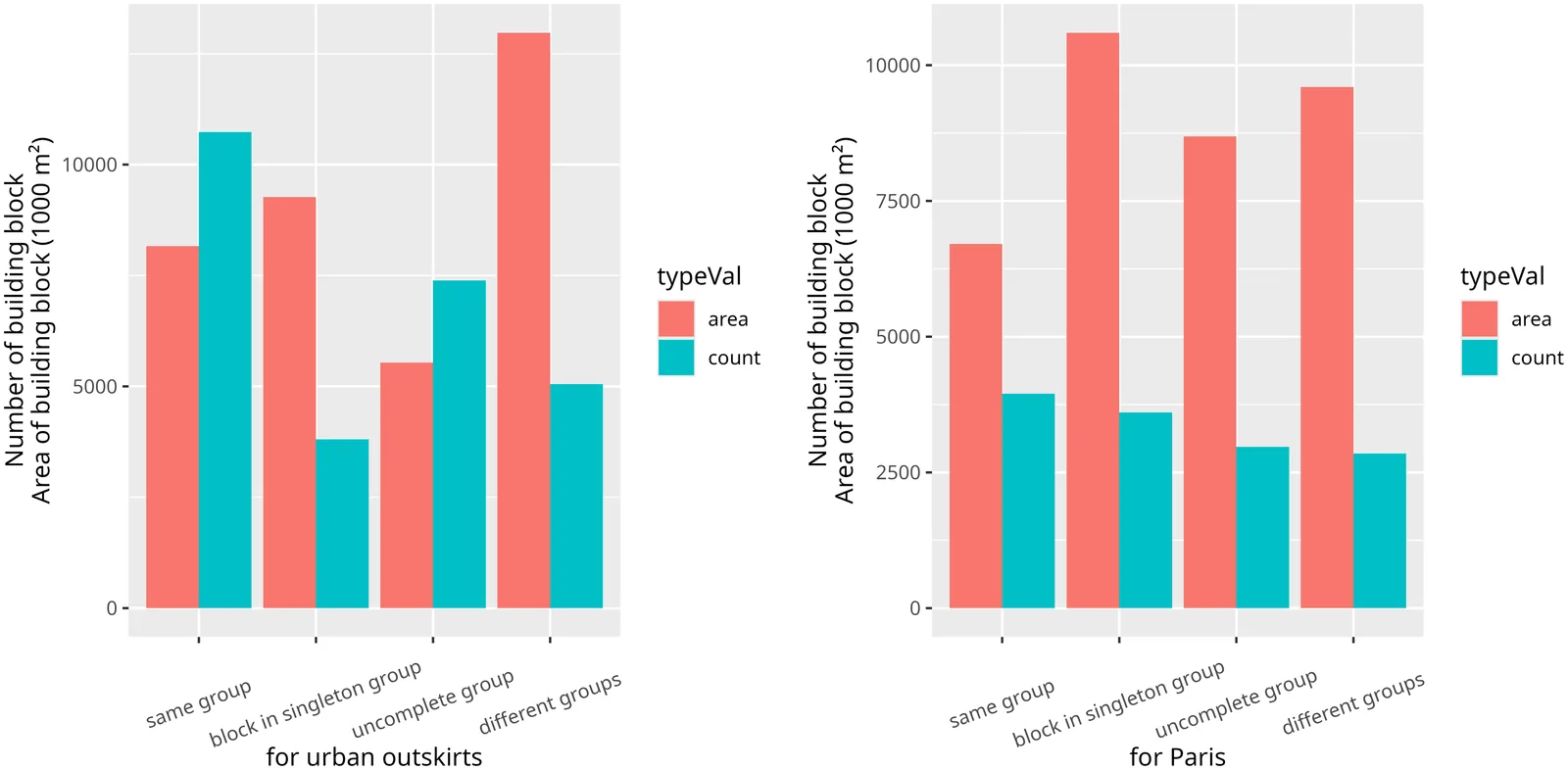
Comparison of the construction of *building group* using links or not, for diverse typologies of French cities.

Results vary considerably depending on which building indicator is used. In general, there are more inconsistencies between methods when floor area is considered rather than building count. It highlights the fact that smaller building blocks are more likely to be linked to the same group, whereas larger building blocks might not be. When the mean number of blocks per group is compared, groups are larger when official links are used. This demonstrates that such links produce groups which would not otherwise be considered.

Results also vary greatly depending on the typology of the community. The method performs well in rural, small and intermediate communes and in urban outskirts, but it is less effective in large urban cities. The main operation carried out when grouping buildings in a non-urban environment is to associate the principal part of a building with its ancillary dependencies, such as garages, which explains its ease of application. Regarding Paris alone, as this city undoubtedly comprises the most intricate building fabric in France, the results are more divergent than those observed in other large urban areas.

It is then possible to state that links bring precision to the formation of groups, but working without links yields results that are realistic enough for smaller buildings. Focusing on the grouping of large *building blocks* might be a worthwhile evolution for this method.

#### 4.1.3 Comparison between building groups and multi-address SIRENE data.

A validation of the composition of groups using a third-party dataset is now presented (Fig 15). The SIRENE dataset, presented on page 12 and in Annex D.2, contains a *complementary information* field. It may be filled with multiple address numbers or an indication implying that the activity is also located at a nearby address. If the complementary information can be processed automatically and turned into one or more addresses, it is possible to check whether the *building group* includes those different addresses. This should be the case, since the continuity of interior space is the main aim of the building grouping. Three situations are envisaged:

- Every complementary address number is included in the *building group*.
- Some of the SIRENE activity’s complementary addresses are found in the *building group*, but not all of them. This case is labelled as **incomplete**.
- The *building group* contains none of the complementary numbers. This case is labelled as **unmatched**.

**Fig 15:**
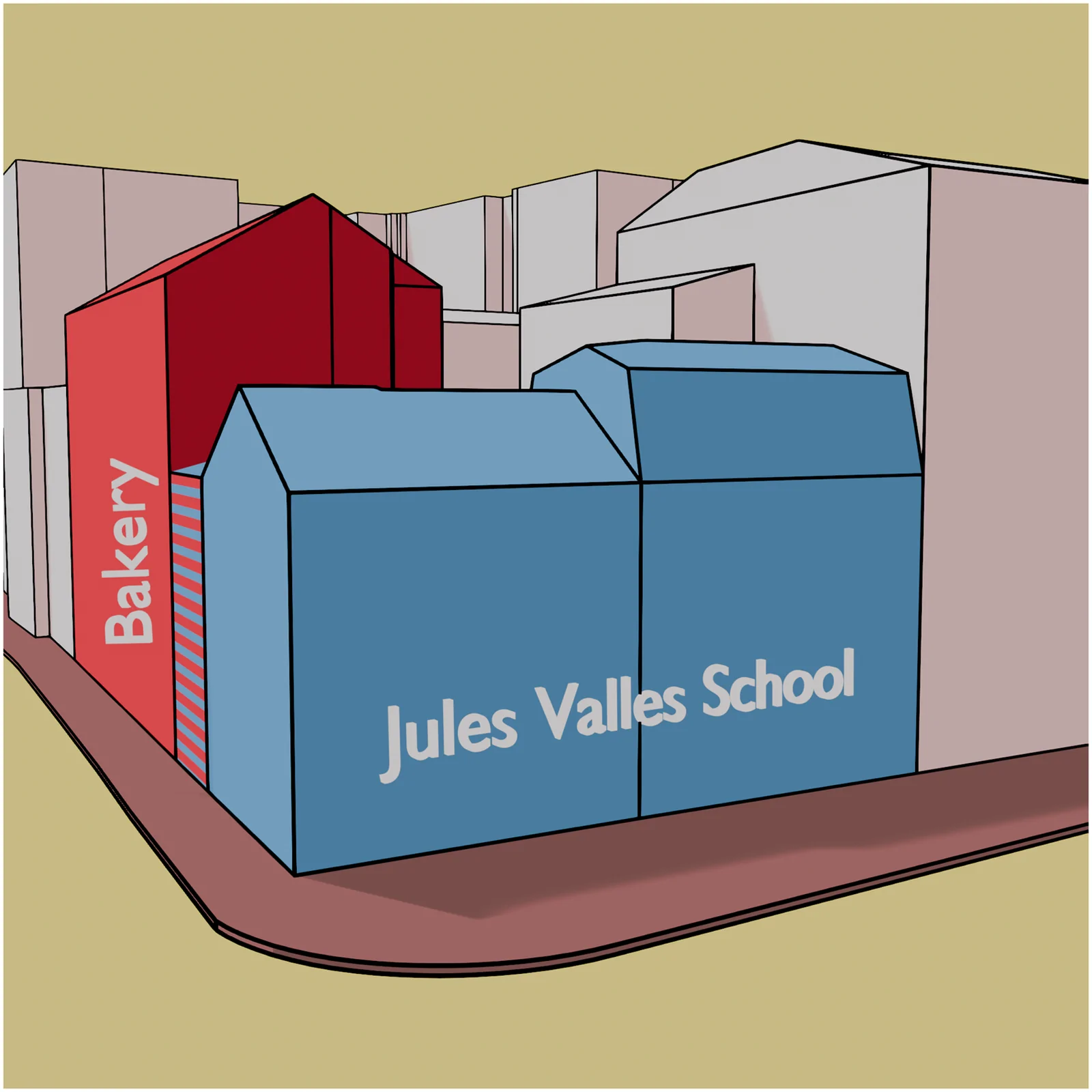
Example of validation of *Building Group* formation with SIRENE activity data. A school is located next to a bakery. The grouping process would assign the intervening building to the bakery’s building, whereas the SIRENE multi-address data indicates this block’s address inside the school.

This analysis is applied to the whole city of Paris for the date 21-12-2019 and uses links for grouping. The codebase can be found at https://s.42l.fr/BIcode.

Results aggregated in Fig 16 show that about four-fifths of the multi-building activities are hosted in the same *building group*. Such results are quite positive for building groups in Paris. In other French cities, this processing chain performs poorly, regardless of the urban type, because a large majority of complementary addresses are unexploitable by the systematic processing. This may be due to the relative nature of the complementary address field, which conveys more informal names in such places. It could also be that the average number of building blocks composing a building group is considerably higher in Paris than outside the capital.

**Fig 16:**
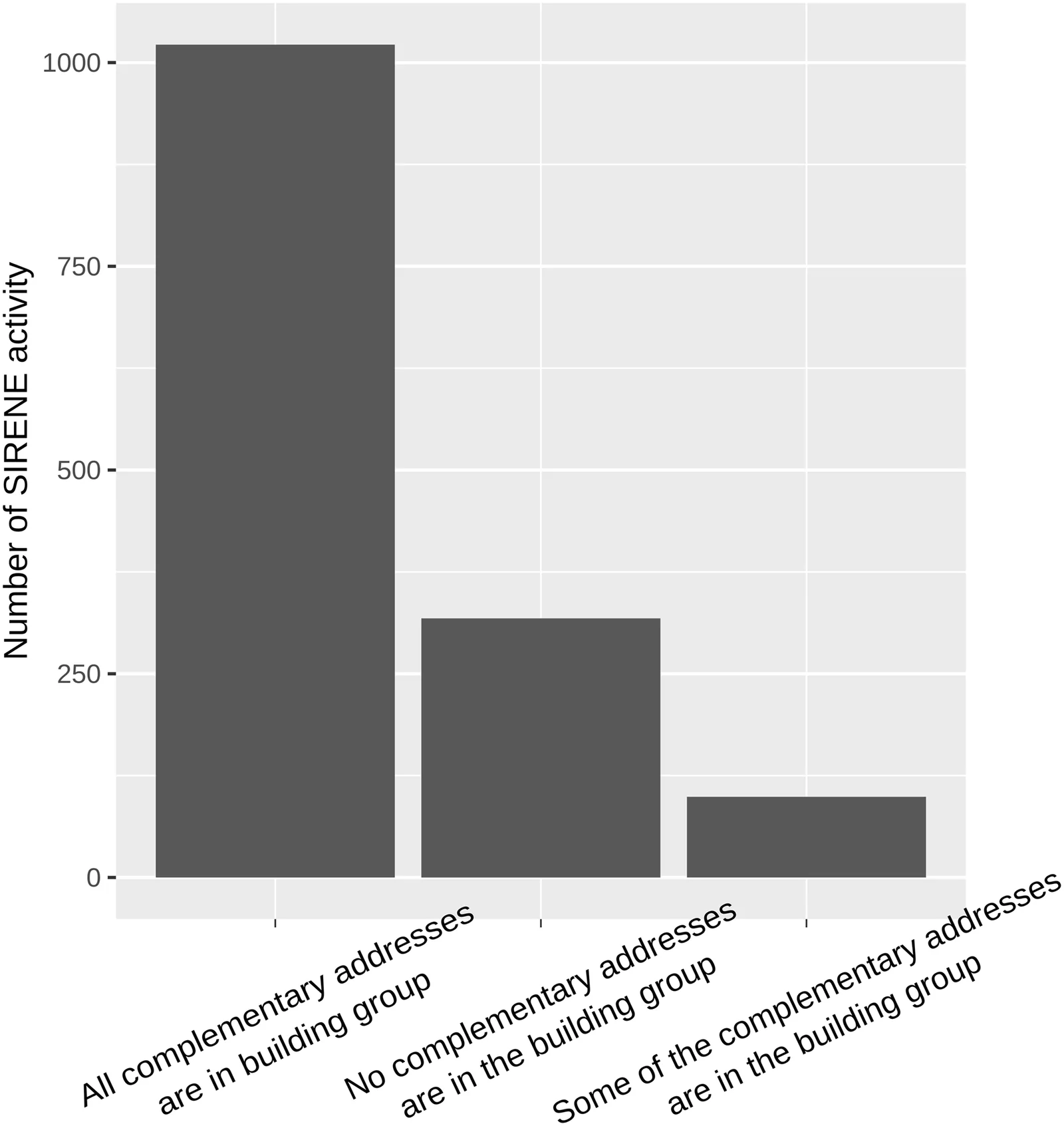
Inclusion of complementary addresses from SIRENE activities within building groups in Paris, France.

This property of the SIRENE data is used for validation only. In order to improve the quality of group formation, particularly outside Paris, it should be possible to incorporate this information into the group-formation method.

### 4.2 Modelling activities: Validation of matchings

The calibration of the matching functions of the conflation process described in 3.2.4 (page 14) has been manually calibrated by adjusting the balance of constants, adding or modifying particular functions, or creating typology correspondences. Matching processes differ according to the process (update, self conflation, conflation of many datasets). Manual validations are performed for each process in order to evaluate the success of the matching task, as depicted in Fig 17.

**Fig 17:**
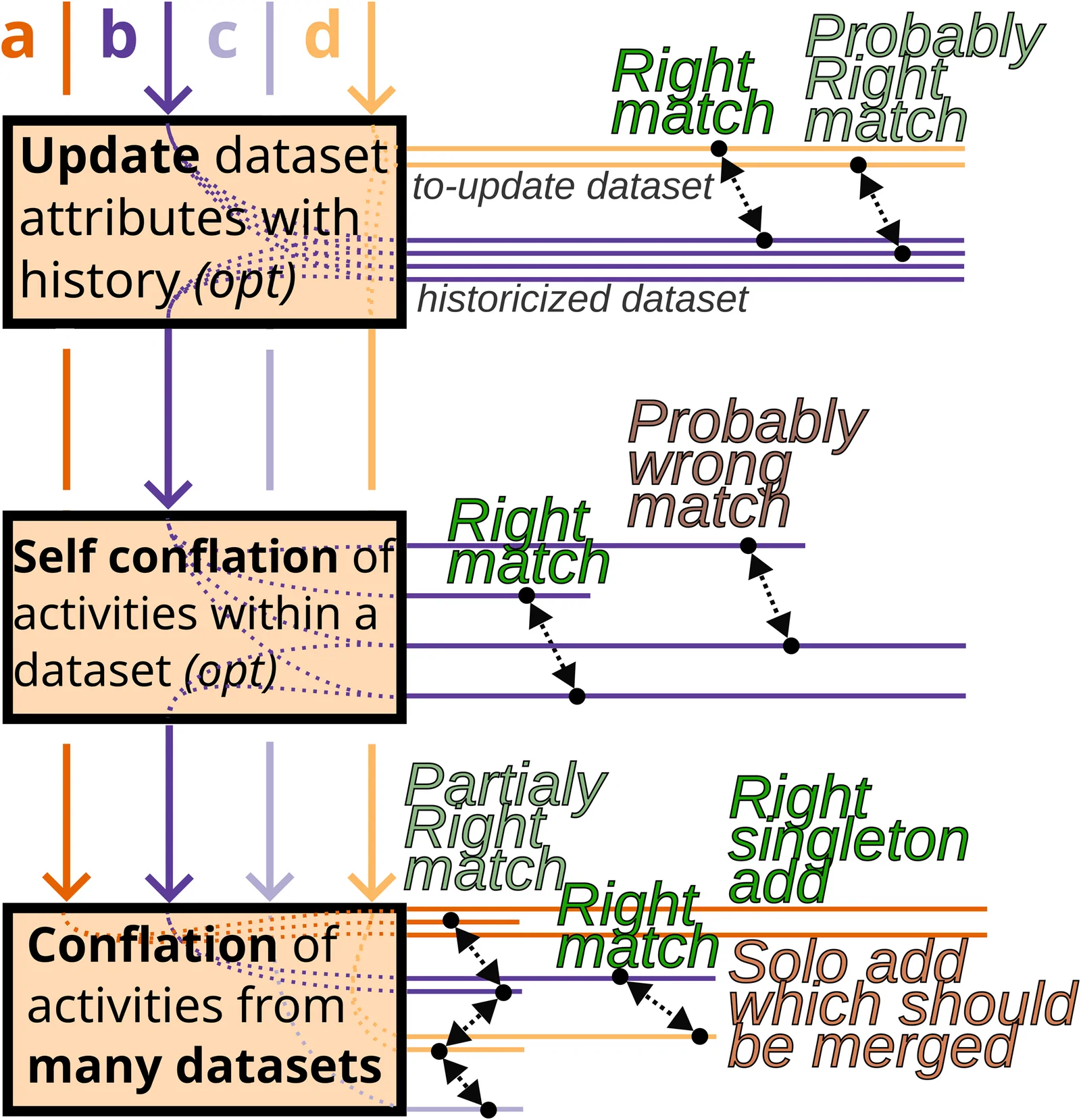
Example of validation applied to the matching of 10 activities from 4 datasets in a single building.

A report is generated, detailing each match with the names and arguments of every matched feature and the result of their merge. Given all the information available on the report (other activities in the same building), each match is manually qualified with an evaluation label. Different qualifications are possible:

- **right**: The match has strong chances to be correct.
- **probably right**: The match seems correct, but there are reasonable doubts (for example, a shop of the same type has unrelated names and there could be multiple occurrences of such activities in the building)
- **partially right**: Most of the activities were relevant to match, but irrelevant activities were also added.
- **impossible to know**: There is no clue for any judgement about the match.
- **probably wrong**: There is a slim chance that the match is right; most likely it is a mistake.
- **wrong**: The match appears to be a mistake.
- **wrong agglomeration**: In the *self conflation* process, an unlimited number of activities can be matched. When a large number of activities are unduly matched, there is a high probability that every other activity in the building matches at least one of the agglomerated activities. If so, a single activity composed of many unduly matched activities is generated. This event is placed in a dedicated collection in order not to overload the *wrong* stock.
- **not applicable**: This label concerns activities where no premises are required. For example, multiple delivery drivers can be registered in the same building, but as they do not require premises to carry out their activities, their conflation is irrelevant.
- **right singleton addition**: In the *conflation of many datasets* process, every single input activity is supposed to have an effective representation on the final result. If an activity is added without being conflated with another activity and there is no reason to think that it was supposed to have a correspondence in another dataset, this characterisation is used. The *right* characterisation is not used in order to avoid overloading the *right* stock.
- **solo additions that should be merged**: In the *conflation of many datasets* process, when an activity is added alone but a correspondence could have been found in another dataset, it falls under this characterisation. It usually occurs in pairs but can also apply to a single activity when the type is vague and could match multiple other activities (for example, an *office in a store*). If the activity could have been matched with an activity that was previously merged with other activities from the same source during the *self conflation* process, it is not counted in this characterisation because the input data are assumed to be correct.

In the matching algorithm, the similarity score must be higher than a fixed threshold. This represents a fundamental parameter for all processes. Two threshold values are jointly evaluated for each process: 0.35 and 0.5.

This task is deterministic, so there is no need for replication. As described in 3.2.1, four datasets are conflated: SIRENE (authoritative), BPE (authoritative), OSM (VGI) and APUR (local). This validation is applied to nine statistical aggregates from three different Paris arrondissements, presented in Fig 18. Only minor differences in geographical context are observed. Consequently, the aggregated results are analysed here. A tabular file aggregating all manual validations is stored at https://s.42l.fr/valMatching.

**Fig 18:**
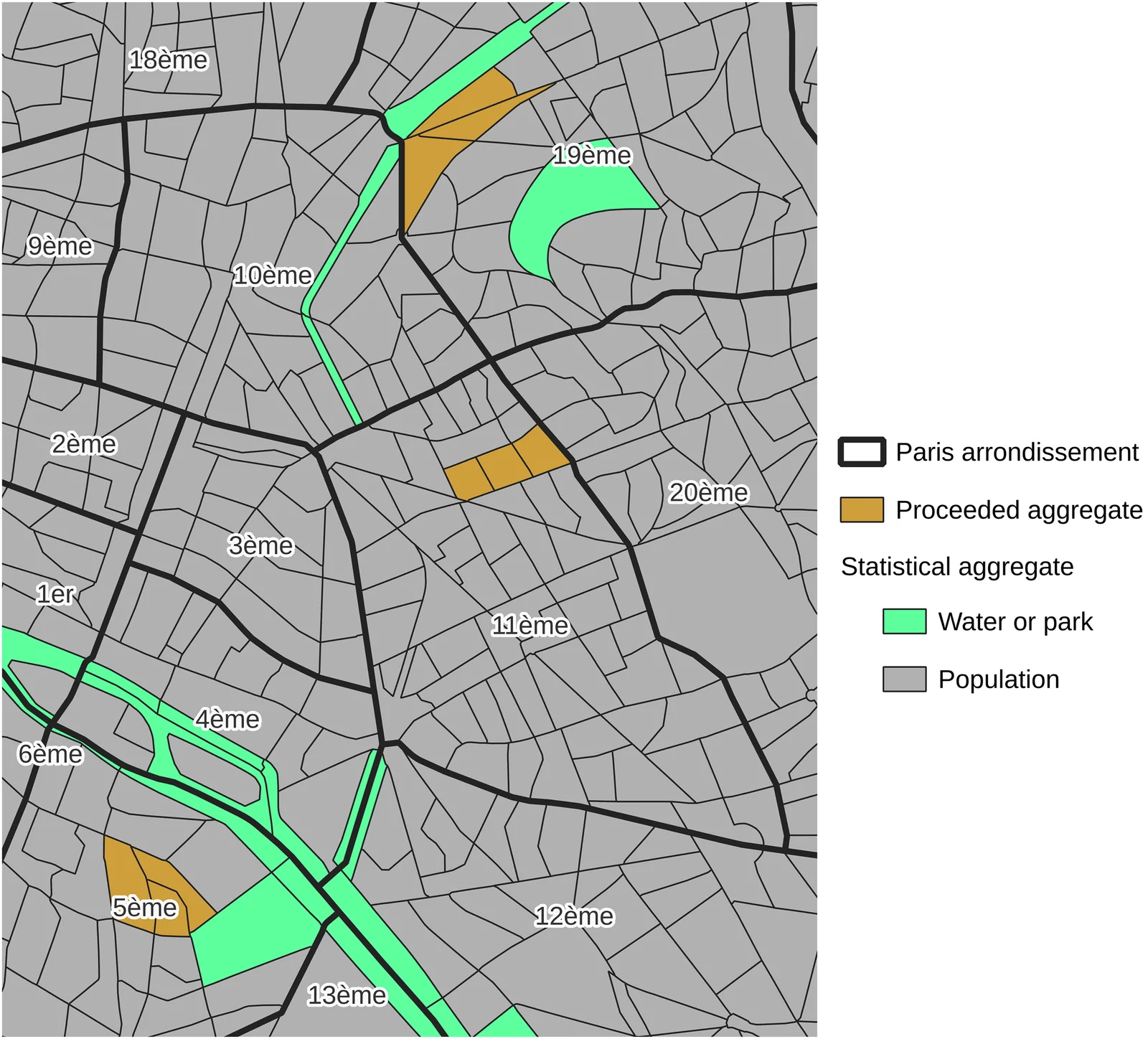
Paris’s statistical aggregates where manual validation of activity matching is applied.

Few limits of this validation process can be highlighted. It aims to evaluate incorrect matches, but it overlooks cases where two results should have matched but did not, apart from the *conflation of many datasets* process. Furthermore, no additional knowledge has been incorporated into the evaluation (e.g., personal familiarity with locations or street-level images from online services), because using such sources raises consistency issues and may not be up-to-date. The strict assessment based on the report may occasionally be inaccurate, but it remains internally consistent.

Each validation evaluates only a single process and not what has been previously done. If the input of a match process is incorrect, but the matching is correctly applied according to its internal design, the result will be characterised as positive, even if the produced result appears wrong. This occurs fairly often after an erroneous *self conflation* process. The subsequent *conflation of many datasets* process then matches the correct activity among multiple other incorrect activities. Scenarios with mixed similarity thresholds have not been tested. Now, the validation results for each process are detailed.

**Update:** In this process, only the *OSM* dataset is updated using the authoritative and historicised *SIRENE* dataset. Both datasets are expected to contain name information. If the names do not match and there is a probability that the activity types have multiple occurrences in the same building, the evaluation label is set to *probably right*. Otherwise, the matches are predominantly classified as *right*. The 0.35 threshold yields more matches than the 0.5 threshold, but the percentage of *wrong* matches increase proportionally to the number of *right* matches (Fig 19).

**Fig 19:**
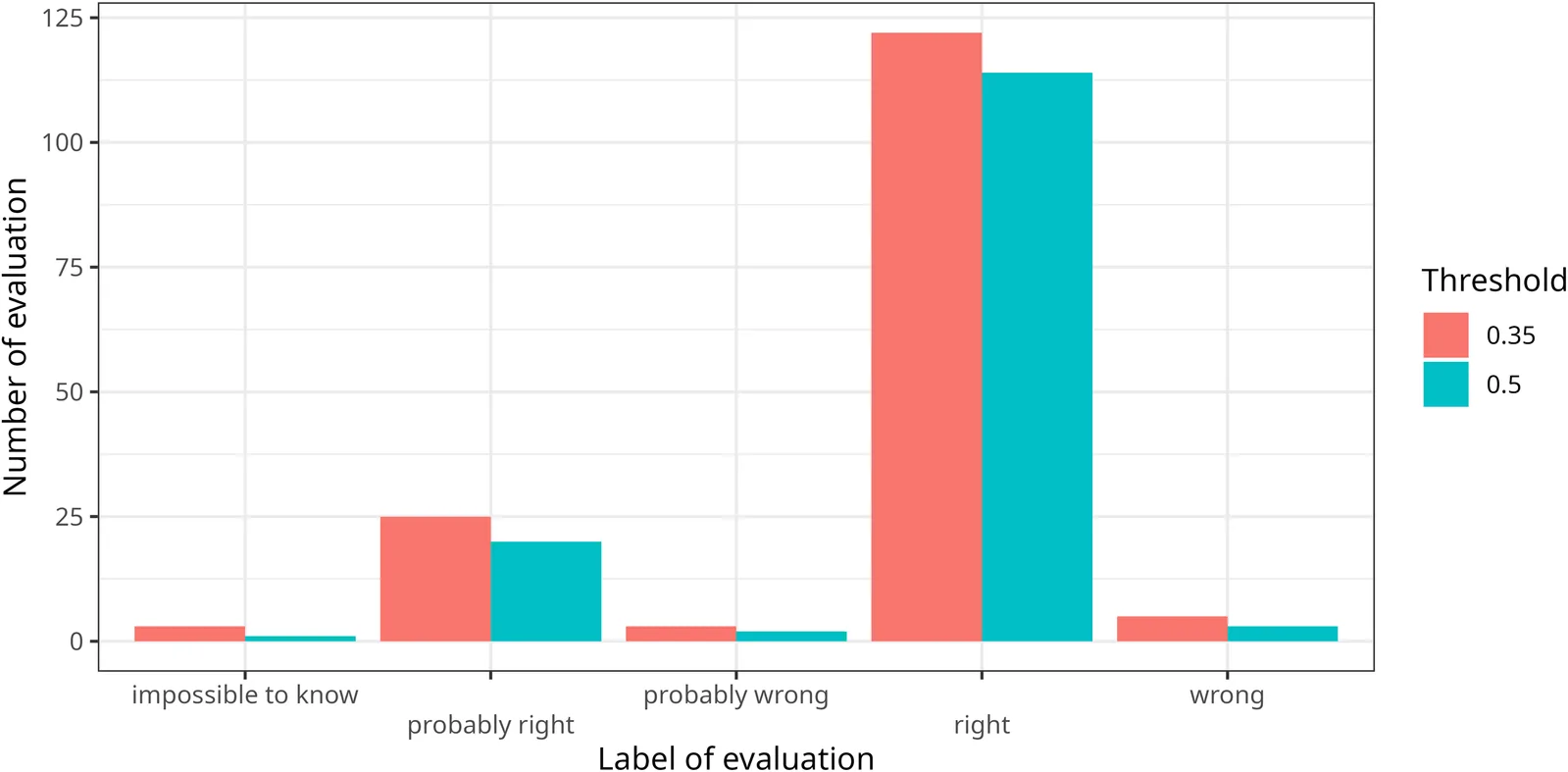
Manual validation of the matchings in the update process on a few statistical aggregates of Paris.

A 0.35 threshold is therefore set as the default for this process.

**Self conflation:** The self conflation process has more incorrect matches. This can be explained by the limited amount of information that can be cross-compared within the same dataset. Fuzzy labels (*probably right*, *impossible to know*) are quite high and highlight this uncertainty (Fig 20).

**Fig 20:**
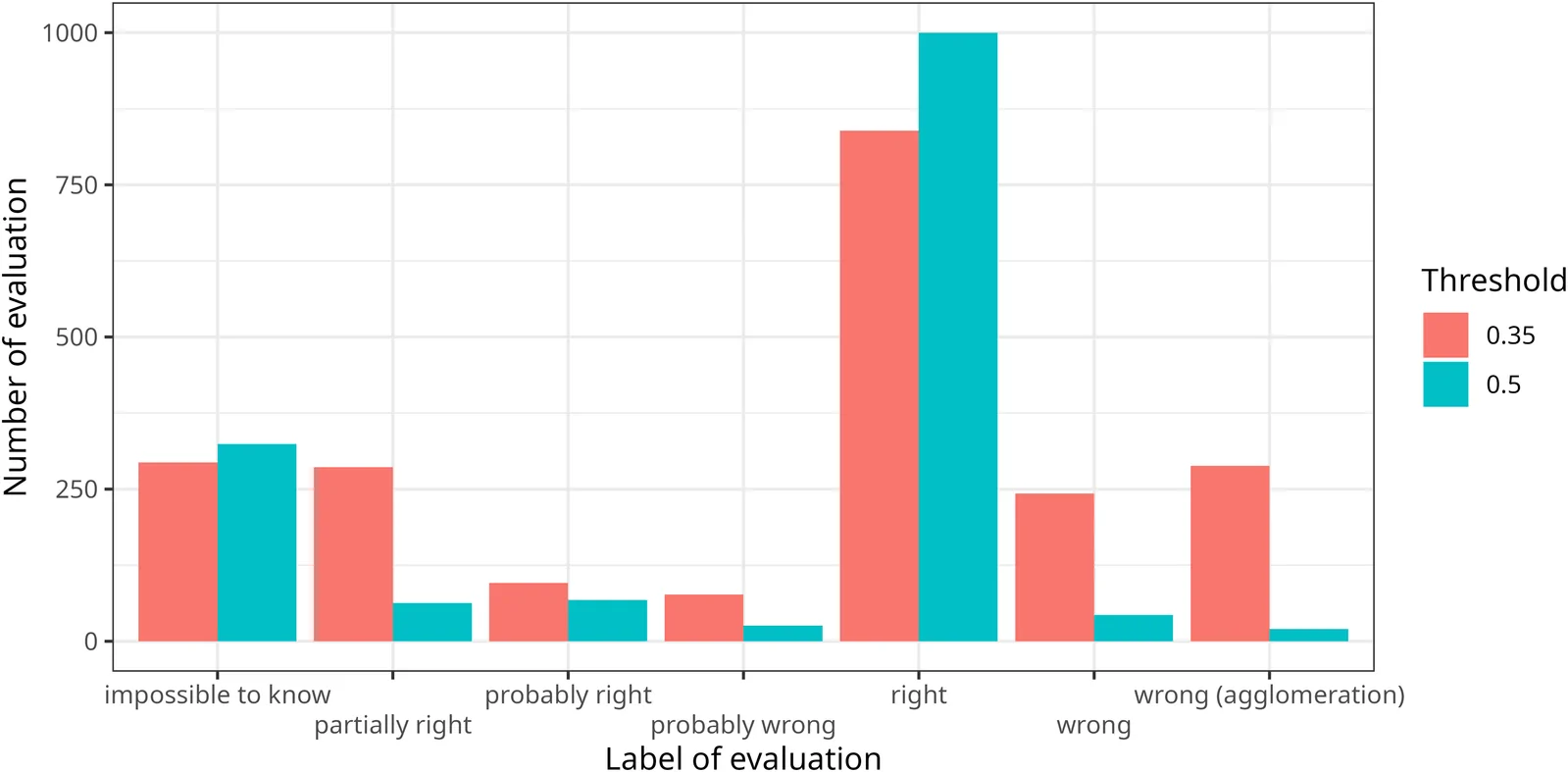
Manual validation of the matching in the self conflation process on a few statistical aggregates of Paris.

Differences between the two similarity thresholds were significant. *Partially right* matches are prominent with the 0.35 threshold, indicating that too many activities are added to the sets of matched activities. The number of *right* matches is higher with the 0.5 threshold, and this difference could stem directly from the lower count of *partially right* matches. *Wrong* matches are prevalent with the 0.35 threshold, suggesting that matches with a similarity score between 0.35 and 0.5 are often incorrect. Additionally, the phenomenon of *wrong agglomeration* is rare when a higher threshold is used.

Due to these results, the 0.5 threshold is set as the default for this process. Because this value differs from the previous process, this adjustment could not have been made without this detailed validation step.

**Conflation of many datasets:** Results for the *conflation of many datasets* process are mostly positive. It is also notable that most of the activities at this point do not match, which means that they are not recovered by other datasets. Each dataset either brings refinement or new activities. No *wrong* matches are reported with the 0.5 similarity threshold, whereas a very small number are reported for the low threshold. Fuzzy labels are also low, regardless of the similarity threshold (Fig 21).

**Fig 21:**
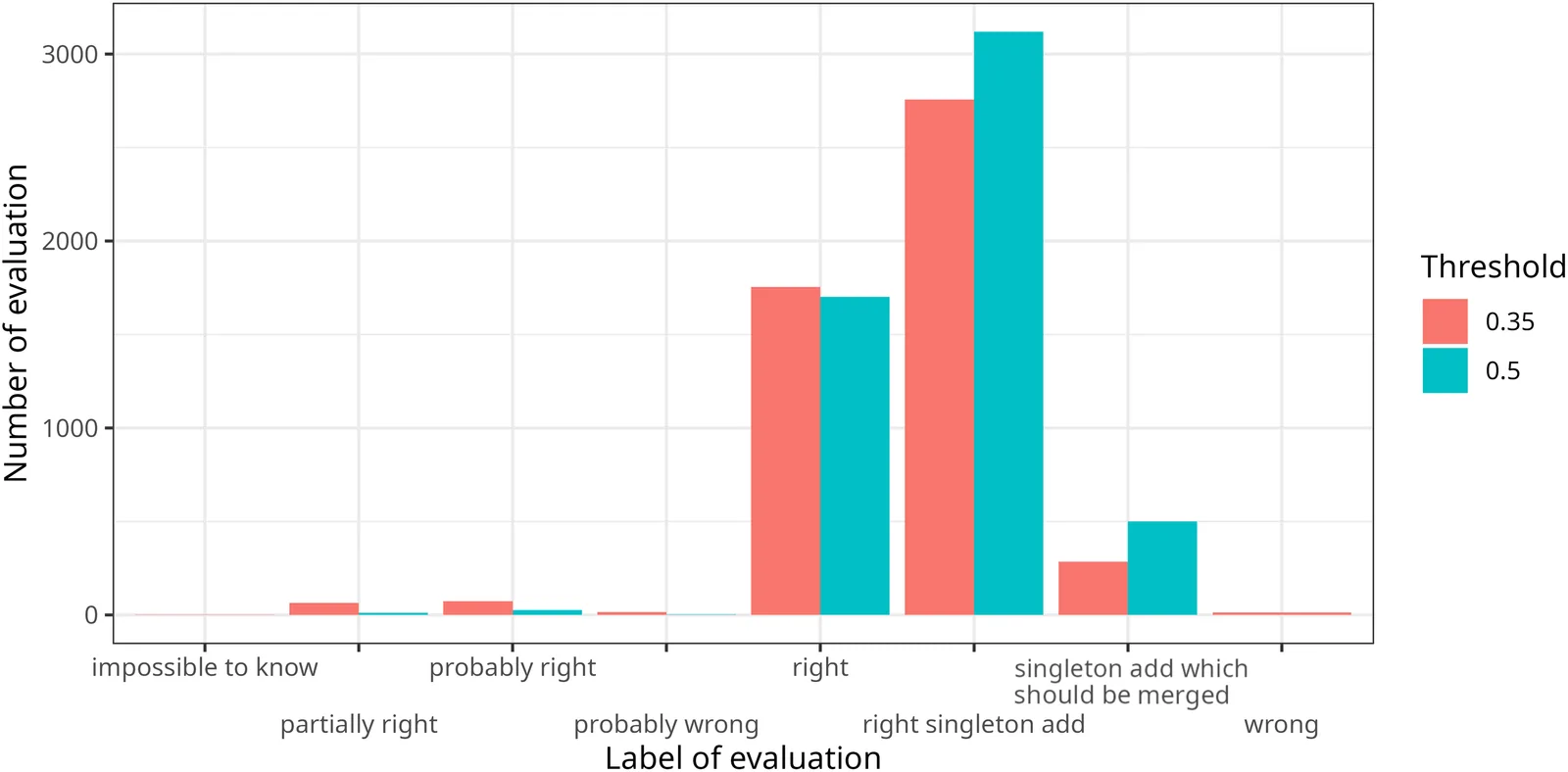
Manual validation of the matching in the conflation of many datasets process on a few statistical aggregates of Paris.

More activities are counted with the 0.5 threshold because fewer had been merged by the *self conflation* process. This leads to more *right singleton add*, but almost double the amount of *solo additions that should be merged* activities. If the threshold of the previous process were higher, it is possible to hypothesise that this process with a low threshold would have yielded even better results. The default threshold of this process is therefore set to 0.35.

A few conclusions can be drawn from this processing chain. A necessary improvement concerns the case where datasets contain multiple occurrences of the same type in the same building (e.g., liberal medical professions are often grouped but administratively separated). When the *self conflation* process is applied to the dataset, those multiple activities would be conflated and would not be available for this *conflation of many datasets* process. It would be useful to introduce an intermediate level that groups multiple similar activities and matches them with one or more activities. It would also be possible to employ a less strict method whereby multiple activities from the same source could be merged using a clustering approach on highly similar activities.

**Sources of characterisation:** It is possible to trace which dataset was the source of the matching that produced incorrect labels, as Fig 22 shows for the **multi-source conflation** process. The local source *APUR* is responsible for 39.6% and 36.6% of the *solo additions that could have been merged* label, but it only accounts for 7.6% of the matched features. This may indicate that its typology is overly detailed and should be grouped or considered at a higher level. Consequently, this analysis suggests that the use of very detailed local datasets may introduce noise rather than increasing precision.

**Fig 22:**
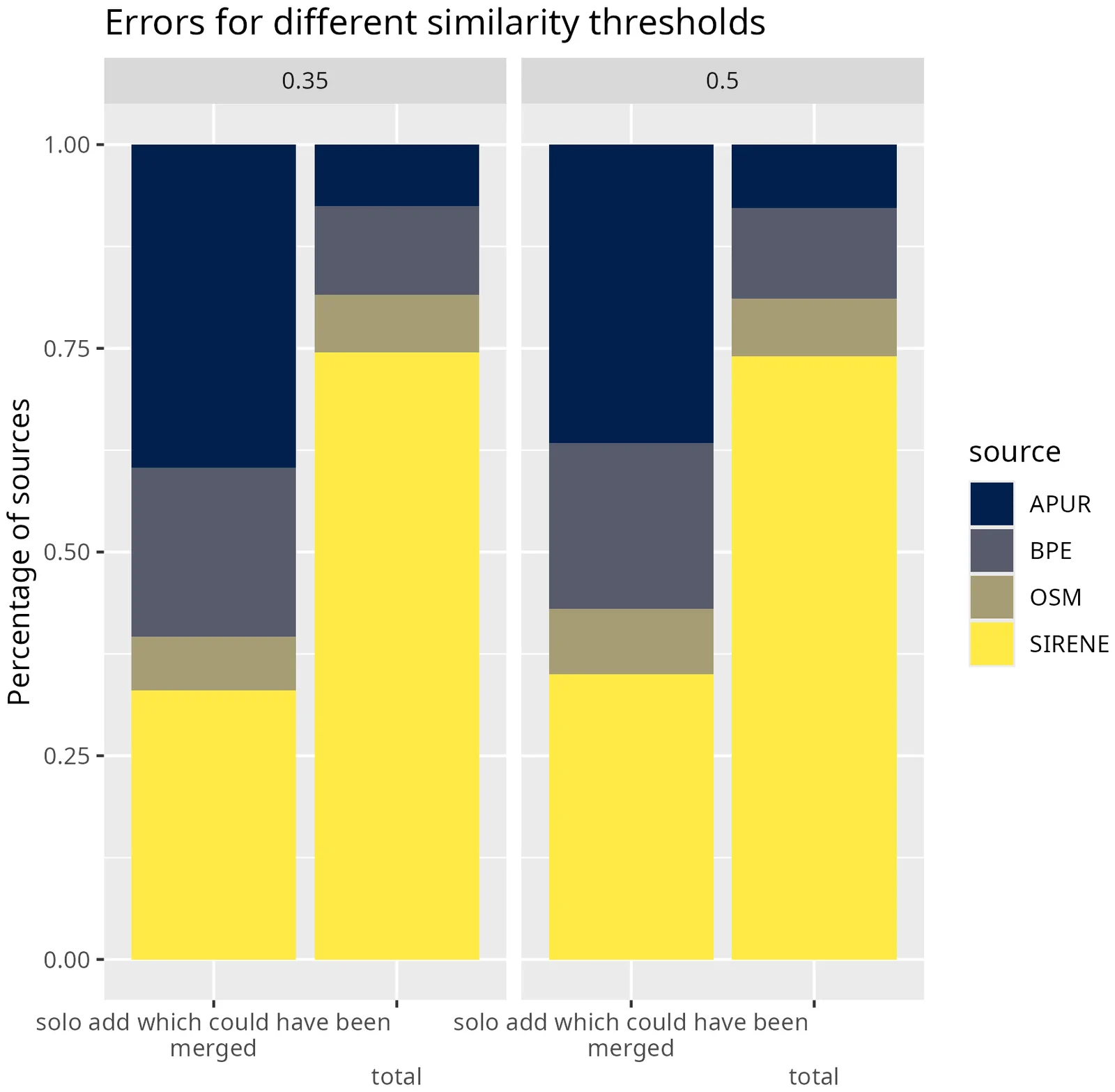
Datasets that cause incorrect results in the manual validation process regarding different similarity thresholds.

### 4.3 Enrichment of the building land use: Validation of area attribution

This section outlines a probabilistic methodology where all calculations are replicated using a seed of 43 for the initialisation of the Random Number Generator to ensure reproducibility. It has been verified that multiple executions with this seed yield identical results.

#### 4.3.1 Generated area vs statistical area information.

The attribution of area to housing units and activities is detailed in Section 3.3.4. Housing unit areas are assigned based on area classes from statistical aggregates, building geometries, and the activities they hosts. Consequently, it is useful to compare the number of generated housing units against each statistical area class.

Due to the allocation of missing housing units into building blocks with unknown values, no statistical aggregate lacking housing units is simulated. Therefore, if a statistical aggregate contains more housing units in the collected data than in the census, the algorithm cannot subtract housing units from the building dataset. The map in Fig 23 illustrates this minor discrepancy in the 11th arrondissement of Paris, while the maps in Fig 24 detail the values for the smallest and largest housing unit area classes. All other classes are available in Annex F. The lower area classes predominantly exhibit an excess of simulated housing units, whereas the higher classes show a deficit. An improvement to this algorithm appears necessary to better satisfy the statistical aggregates.

**Fig 23:**
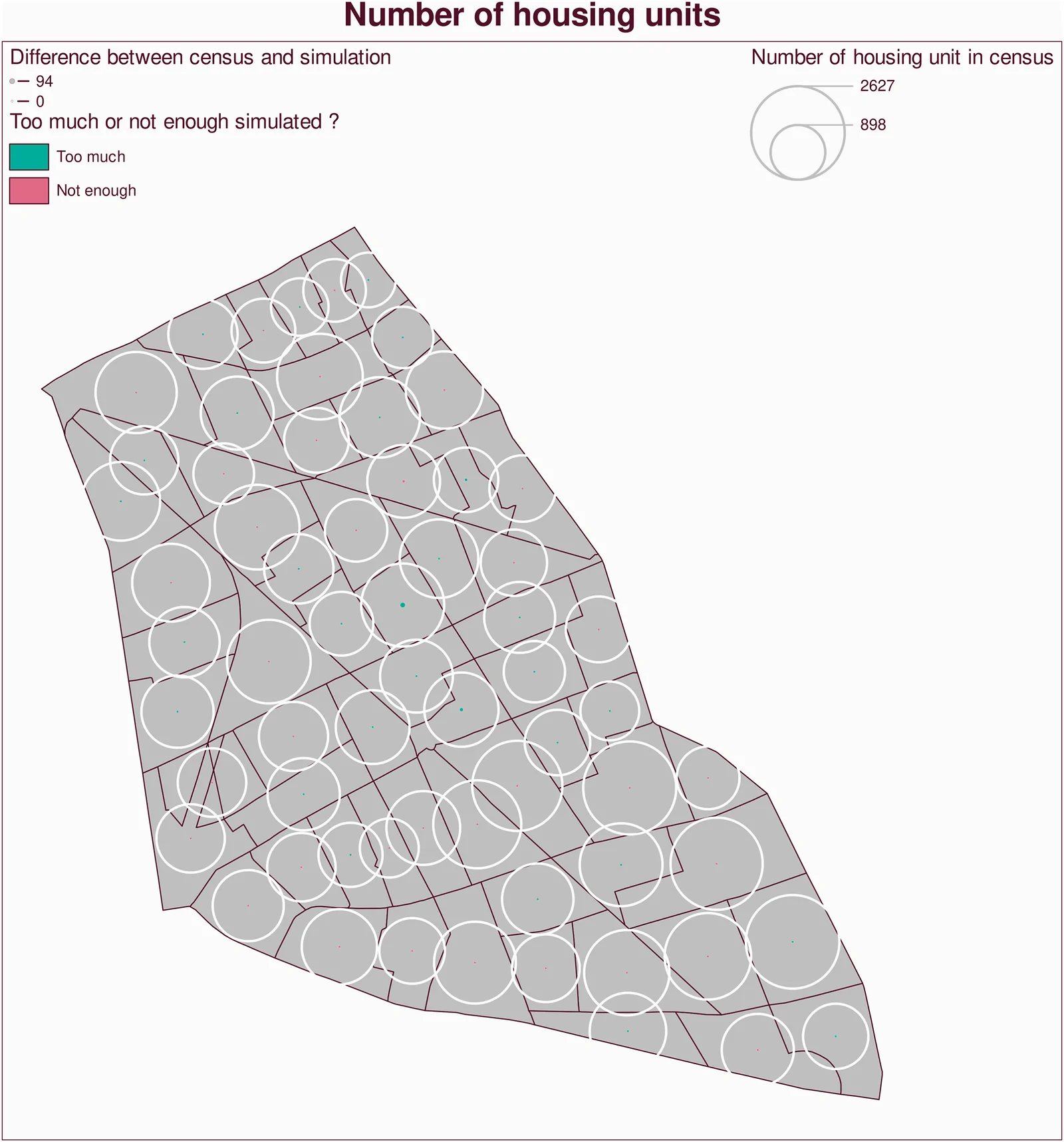
Discrepancy between generated and statistical housing units in the IRIS zones of the 11th arrondissement of Paris.

**Fig 24:**
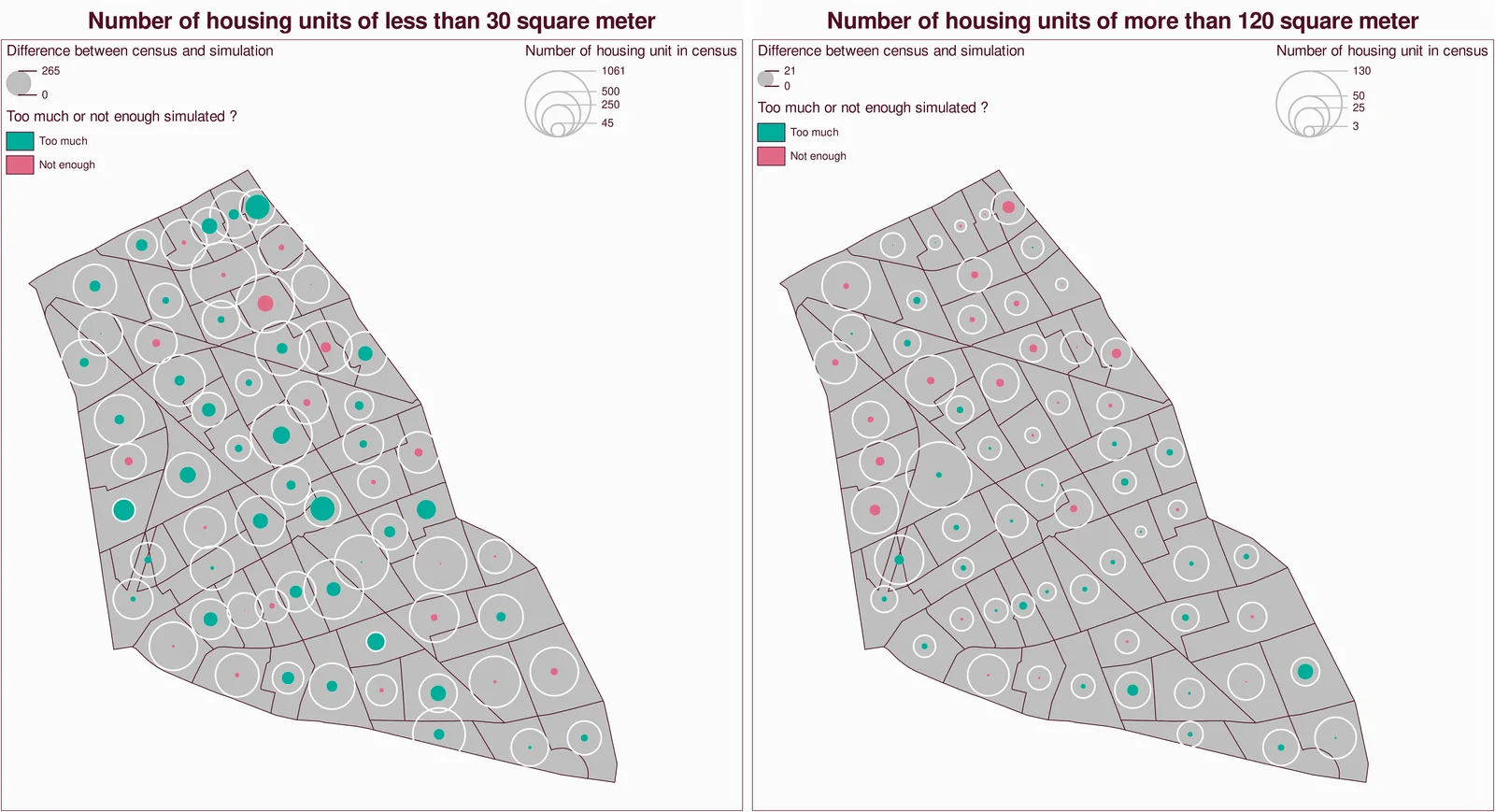
Discrepancy between generated and statistical housing units in the IRIS zones of the 11th arrondissement of Paris.

#### 4.3.2 Maximisation of housing unit area.

In order to overcome the limitations highlighted in the previous section, the process described in 3.3.4 includes a maximisation of the housing units area, bounded by the physical area available in each building. The necessity of this step and the number of iterations can be questioned. This processing chain presents an analysis of how this maximisation succeeds in meeting the objectives of housing creation specified by the statistical aggregates. An optimum number of replications is estimated to improve overall workflow efficiency.

This process is applied to the three studied arrondissements of Paris. Building groups and activities are imported and the process of housing unit area attribution is analysed. The number of iterations of the built-in maximisation process increases from 1 to 70. This process calculates, for each of the 7 area classes, the absolute difference between aggregated statistical values and the generated set of housing units. This whole processing chain is replicated 50 times. The codebase for this experiment can be found at https://s.42l.fr/housingUseCase.

Fig 25 illustrates the satisfaction of the objective relative to the number of iterations. In each arrondissement, it appears essential to employ the built-in maximisation process, as even a few replications yield housing unit sets that are significantly more compliant with statistical aggregates. However, for arrondissements 5 and 11, excessive replication should be avoided; beyond a certain point, the results become overly maximised, causing the absolute difference to increase. Consequently, a default value of 10 replications has been established.

**Fig 25:**
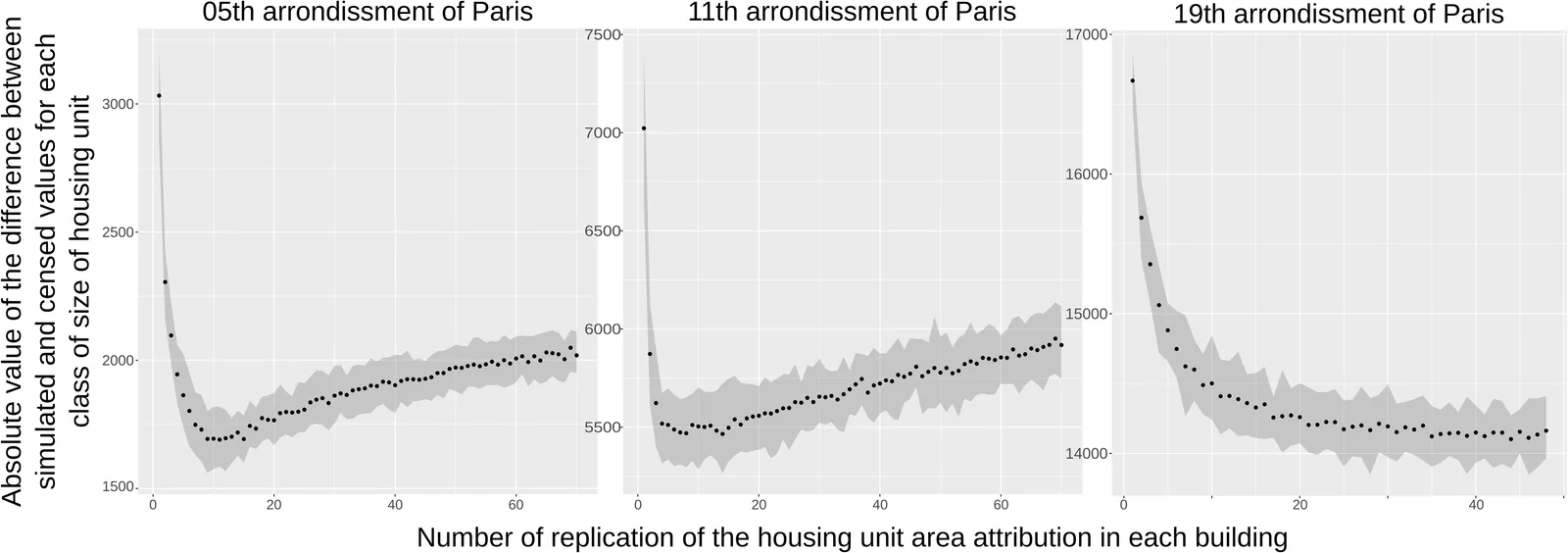
Absolute difference between generated and statistical housing units by area class across replication numbers of the built-in maximisation process.

### 4.4 Validation with ground truth

#### 4.4.1 Confrontation between reported to be closed activities from historicised and OSM datasets.

The *update* process defined on page 15 matches activities between an out-of-date dataset and a historicised dataset in order to update particular attributes. It is possible to isolate cases where the matched historicised activity is reported as closed whilst the activity to be updated is supposed to be open. The highlighted conflicts between those two kinds of datasets are resolved by a ground truth control reflecting the real situation. An analysis is then carried out to examine this dataset conflict.

The historicised dataset comes from the SIRENE database, which collects administrative statuses about legal units and their establishments, as well as every change that occur over time. OSM data are crowdsourced and might not always be up to date. Both datasets have recent and corresponding release dates (SIRENE release of 01/02/2025, OSM data dump of 18/02/2025). This processing chain selects a list of matched activities that are open in the crowdsourced OSM dataset but are reported as closed in the historicised authoritative SIRENE dataset. Each activity’s location is visited to resolve the conflict between the manual mapping and the abstract administrative status. This processing chain has been applied to the city of Courbevoie (92), located in the Paris suburbs on the border of La Défense business area. The dataset used is the most recent generation described in 5 and the manual gathering took place on the 5th and 7th of March 2025. The code used to generate the reports can be found at https://s.42l.fr/OSMopen.

157 matches of activities were visited and manually labelled. Three general observation labels are possible, each with specific cases. The first label is attributed when the visited activity is reported as **closed**. It indicates that the OSM database is lagging behind the SIRENE database. A few specific cases can further qualify that closure information, the main ones are:

- **close and vacant**: the former activity is still visible but has ceased operating. The *was:* tag exists in OSM and should be applied in this case.
- **closed and replaced with same type**: a new activity of the same type has replaced the former matched activity. Updating the OSM database is less impactful because only secondary characteristics need to be updated.
- **closed and replaced with other type**: a new activity of a different type has settled in the former activity’s premises.

The second label is a **mismatch**. The match of activities could be completely wrong, where the types don’t match at all or where both activities coexist. A second specific case is when an OSM activity has been matched with an older version of the SIRENE dataset. During the matching process, the OSM activity is compared with multiple versions of the historicised activity because no time information was gathered concerning the creation of the OSM activity. The SIRENE activity with the highest similarity score is then chosen. When *similarity score* are equal, a random activity is selected. This can lead to multiple erroneous matches, especially when no names are provided.

The last general observation label is applied when the match is correct and corresponds to the activity at the visited location, which is nevertheless **open**. This situation can arise because an activity has changed ownership and has been reported as closed. In reality, the OSM activity is open while the corresponding SIRENE activity was not found. In the model, the OSM activity will be considered closed, but there is a high likelihood that another SIRENE activity is registered under a different typology and kept in the final result.

Details of the ground truth data gathering is available in this file https://s.42l.fr/OsmClose and is summarised in Fig 26.

**Fig 26:**
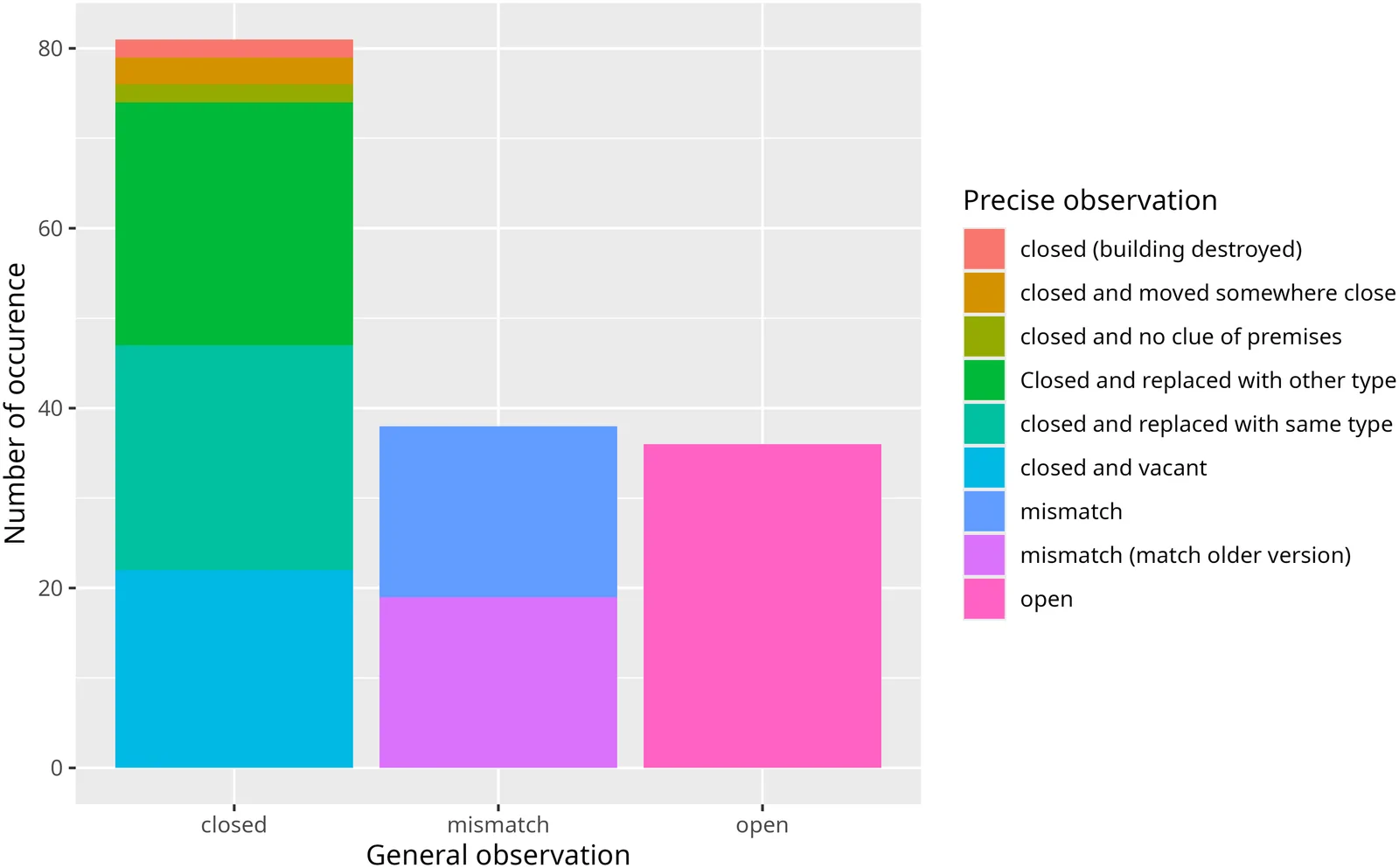
Result of ground truth data gathering on the confrontation of two datasets regarding the closing of shops.

Rightly closed activities represent 52% of matches, 25% were mismatched and 23% were still open. The relatively high number of *mismatch* or *open* matches could be explained by inconsistencies in administrative statuses. As an example of an *open* problem, the shop *les jardins de Courbevoie*, registered as a retailer of vegetables and fruits, and located at *11–13 avenue Léonard de Vinci*, is reported as closed in the administrative records but not in OSM. Visual examination shows that this shop is still open and OSM is correct. A closer look at the SIRENE data reveals that, although this shop was reported closed in September 2023, a new shop opened with a similar name (*la corbeille de Courbevoie*) but with a different type (wholesale of vegetables and fruits) and no continuity in the legal unit. The visual inspection also suggests that the shop continues to operate as a retailer rather than wholesaler. Additionally, a few large companies were unduly matched and flagged. For instance, the headquarters of a very large car-glass company is reported as closed, but this only concerns the investment branch of the firm. It is therefore considered a *mismatch* rather than an *open* label because the matching algorithm identified a closer relation on this sector while it should have matched another branch of the company.

Half of the *mismatched* activities resulted from the selection of an incorrect version of the historicised data. Such cases could be easily avoided in future improvements of the matching algorithm.

The three principal kinds of closure were approximately equally distributed. These proportions should reflect the economic vitality of the reported area.

This processing highlights the difference between two types of activity inventory: administrative statuses and public gathering/perception as transcribed by the OSM contributions. It also isolates specific points in order to improve the matching algorithm, for example, the introduction of opening statuses or date of the entry into the selection criterion. The validity of such results must be put into perspective because of the strong dependency on the location and number of OSM contributors living in or visiting the area. It would also be interesting to apply this processing to diverse types of communes.

It also demonstrates that the algorithms developed in this work are usable for various purposes. Here, feedback from collaborative and open datasets is generated. Even if its limited results do not allow systematic use, they could provide great help to OSM contributors who keep the database up to date. A list for all of France could easily be generated, upon each monthly update of the SIRENE dataset, in order to indicate potentially updatable elements for OSM contributors. A few initiatives by the French OSM community (for example https://www.complete-tes-commerces.fr/) point in this direction.

Dynamic spatial analysis could be performed on a larger set of collected data. Even though Courbevoie has two major malls on its municipal territory, very few matches were found within them. Shopping streets experience frequent shop turnover, and their premises may be more prone to be replaced by other shops than elsewhere. Different clusters could also be formed because some activity types are more likely to remain open or be replaced by the same type (e.g., banks, bakeries), whereas declining activities are more likely to become vacant or be replaced by a different type (e.g., ready-to-wear fashion store).

#### 4.4.2 Validation of the whole model.

A more general ground truth validation is finally realised. Its main objective is to compare the model’s results with manually gathered information about buildings and their activities. A data-gathering session was organised by the two authors of this article on 17 February 2025 concerning the sectors of the 19th arrondissement of Paris already analysed in 4.2 (Fig 27). Due to the large amount of manual processing, only two of the three statistical aggregates were collected. Such observed data are compared with model generations on 1 February 2025. This processing chain yields a global conclusion on the results of the various modules presented in this work and initiates a few improvements. A transcript of the observations can be found at https://s.42l.fr/validModel, and the modelled data can be found at https://s.42l.fr/validModelData.

**Fig 27:**
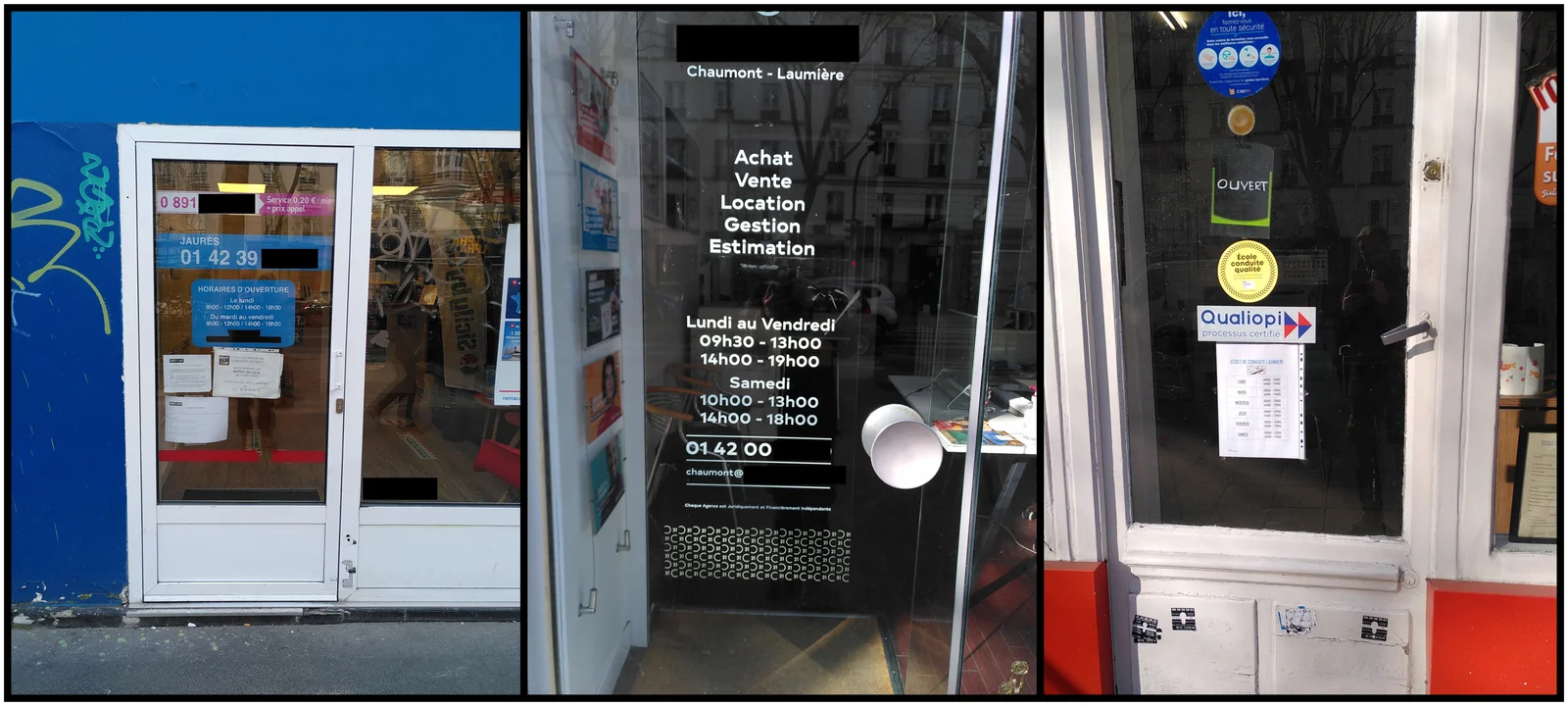
Example of data-gathered shops in the 19th arrondissement of Paris, displaying their opening hours. Pictures taken by the authors and protected under a CC BY license.

As it was not possible to enter buildings or inner courtyards, the *observed* information could attest the presence of an element from the limited street point of view. The absence of an activity could only be conclusive when it is specified to be open to the public. Details are also collected, such as the number of mailboxes or doorbells in a building or the displayed opening hours. No contact was made with owners of activities or inhabitants of buildings. Particular attention is drawn when synthetic information has been generated in the model.

Results about the building model or the activity model and are presented separately.

**Building grouping** Observed data about *building groups* are compared to two different model generations: one with links between addresses and buildings and one with only addresses and parcels. A valid observation consists of multiple visible addresses spotted on the apparently same building, a backyard that appears to be the only way to access different building blocks, or a single activity observed at different addresses. It was not possible to enter closed buildings backyards and hallways in order to search for functional continuity. Therefore, an observed group that is not represented in the model is more significant than the converse. The presence or absence of address numbers displayed at building entrances is also observed and compared with the model’s addresses.

Fig 28 shows the comparison between groups of buildings from generated and observed data. Most of the *building groups* were reported to be *correct*. A difference between addresses (labels *one or more addresses modelled* or *observed*) implies that the observed and modelled building groups were the same. It is frequent that address signs are missing and therefore *not observed*. When an address is *not modelled*, it means that the address was assigned elsewhere without influencing the building-grouping process. This case is rare. The biggest error occurs when the grouping of buildings in the model wasn’t observed in reality (label *multiple observed blocks in one modelled group*). As stated earlier, our observation cannot confirm that there was no connection between such building blocks. Observed groups that aren’t grouped in the model are undoubtedly errors, but they are also less frequent.

**Fig 28:**
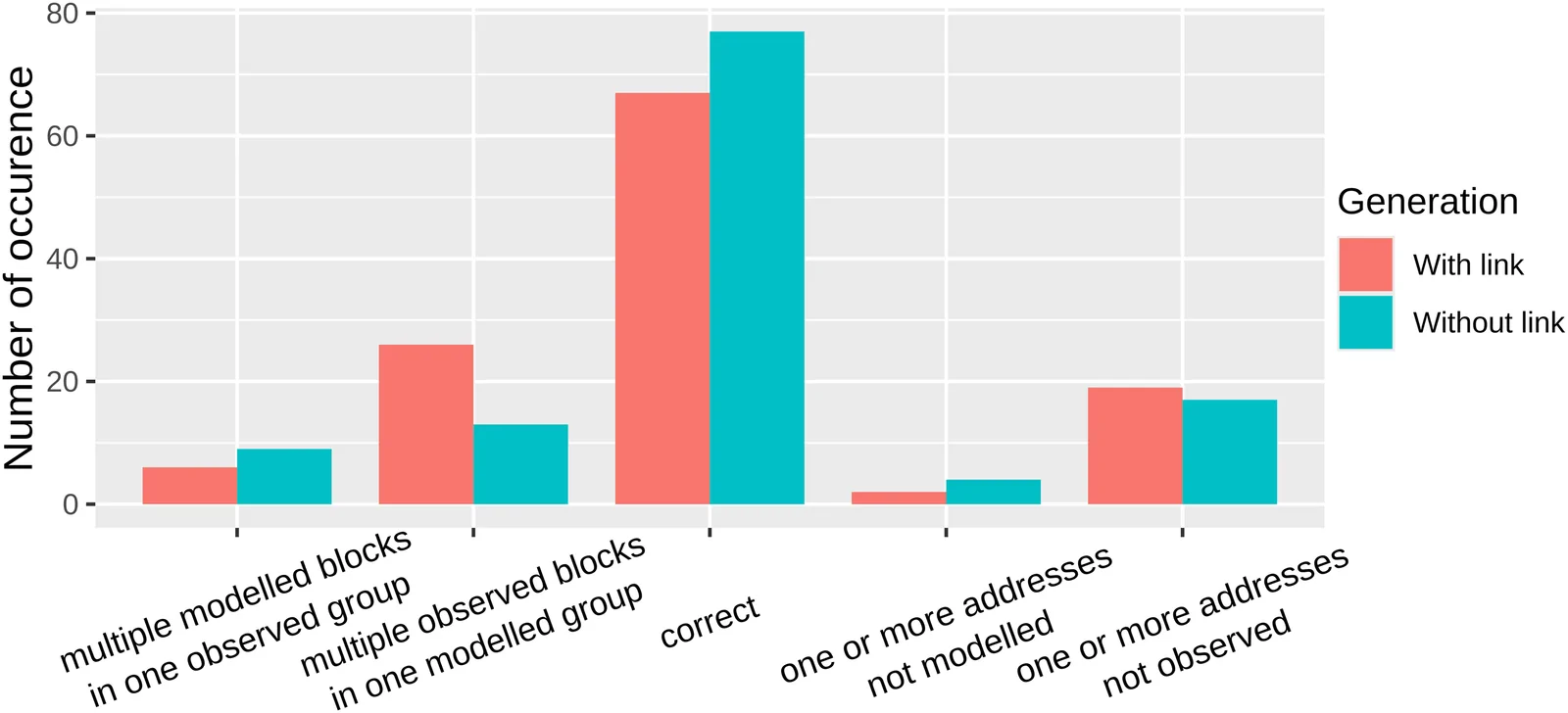
Observation about building group formation.

Surprisingly, the generation without links produces results that are more similar to the observed data. This may be due to the formation of a few very large building groups when links are used. This finding is encouraging for the use of this model in contexts where such complex links are unavailable.

**Mailboxes** Mailboxes were counted when they were visible, either through glass-doors or inside open halls. In the usual case, each housing unit should possess at least one mailbox. An effort was made not to count boxes dedicated to advertising. They are summed if the *building group* has multiple visible entrances, and compared with the *building block* when only part of the group’s entrances were visible and accessible.

Fig 29 shows the difference between observed mailboxes and modelled housing units. These values are often similar and sometimes exactly equal. In very rare cases, mailboxes have been observed when no housing units were modelled. This may be due to companies possessing mailboxes, which can also explain cases when more mailboxes were observed. When more housing units were modelled, it is possible that not every housing unit possessed a mailbox; however, it is more likely that the grouping was incorrect or that the input data themselves are erroneous.

**Fig 29:**
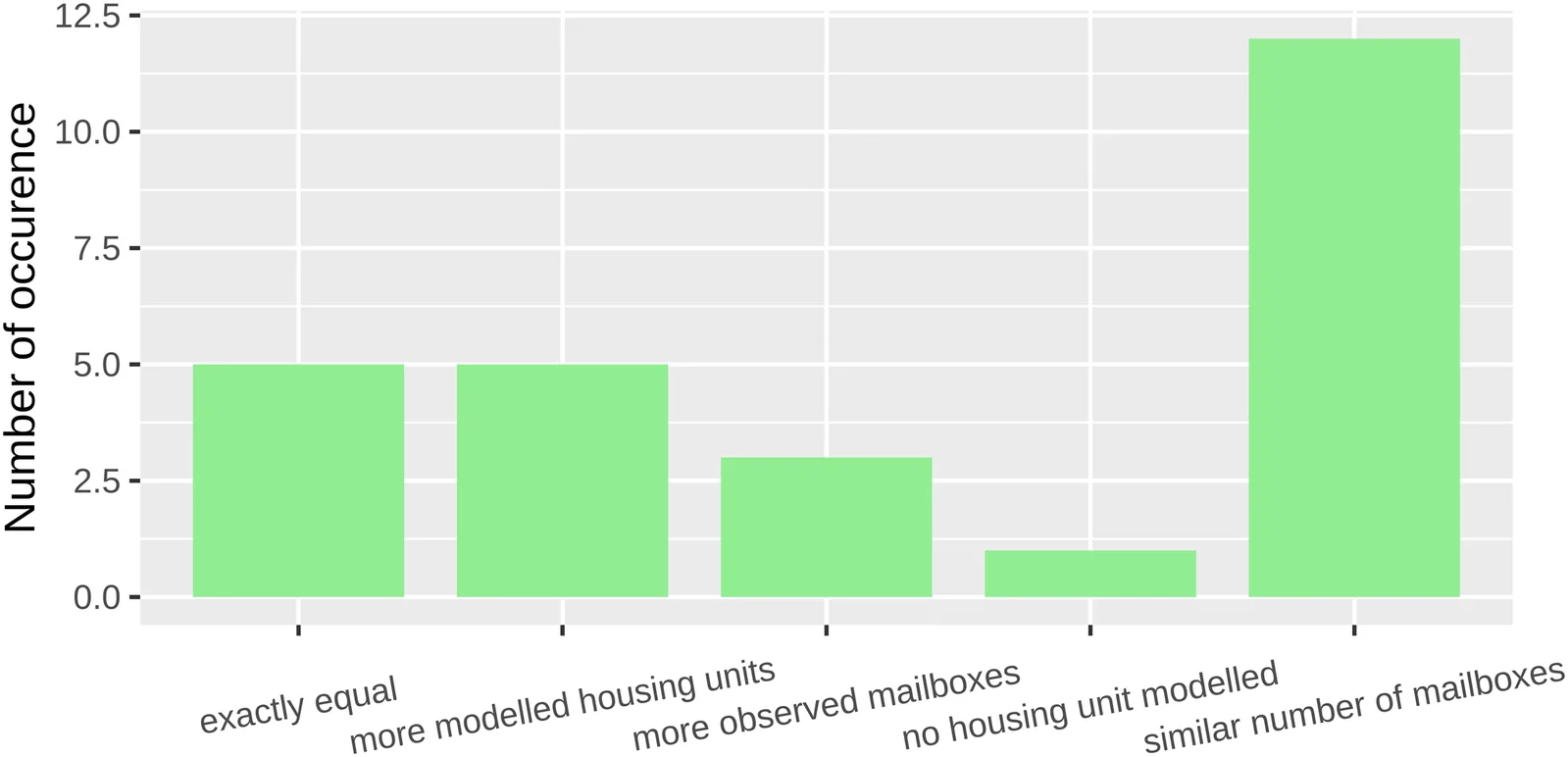
Confrontation of observed mailboxes and modelled housing units.

**Activities** Observed activities are compared to three model generations, each produced with building groups created using links:

- *4 dataset*: every dataset presented in 3.2.1, namely SIRENE and BPE (authoritative), APUR (local) and OSM (VGI);
- *3 dataset*: SIRENE, BPE (authoritative) and OSM (VGI)
- *2 dataset*: SIRENE and OSM datasets

A general observation was made on the building’s ground floor or at any indication of activity (mailbox, plaque or advertising).

Fig 30 shows that almost every observed activity had a validated correspondence in the model’s results. A few activities were modelled without a premises yet were observed. Finally, a few observed activities were not found in the model. No search for neighbouring activities was carried out in those rare cases. Regarding the type of the *not-found* activities, *vacant* premises are frequent. Such a specific type of activity should require dedicated processing. Moreover, when additional datasets are used for generation, more activities are matched.

**Fig 30:**
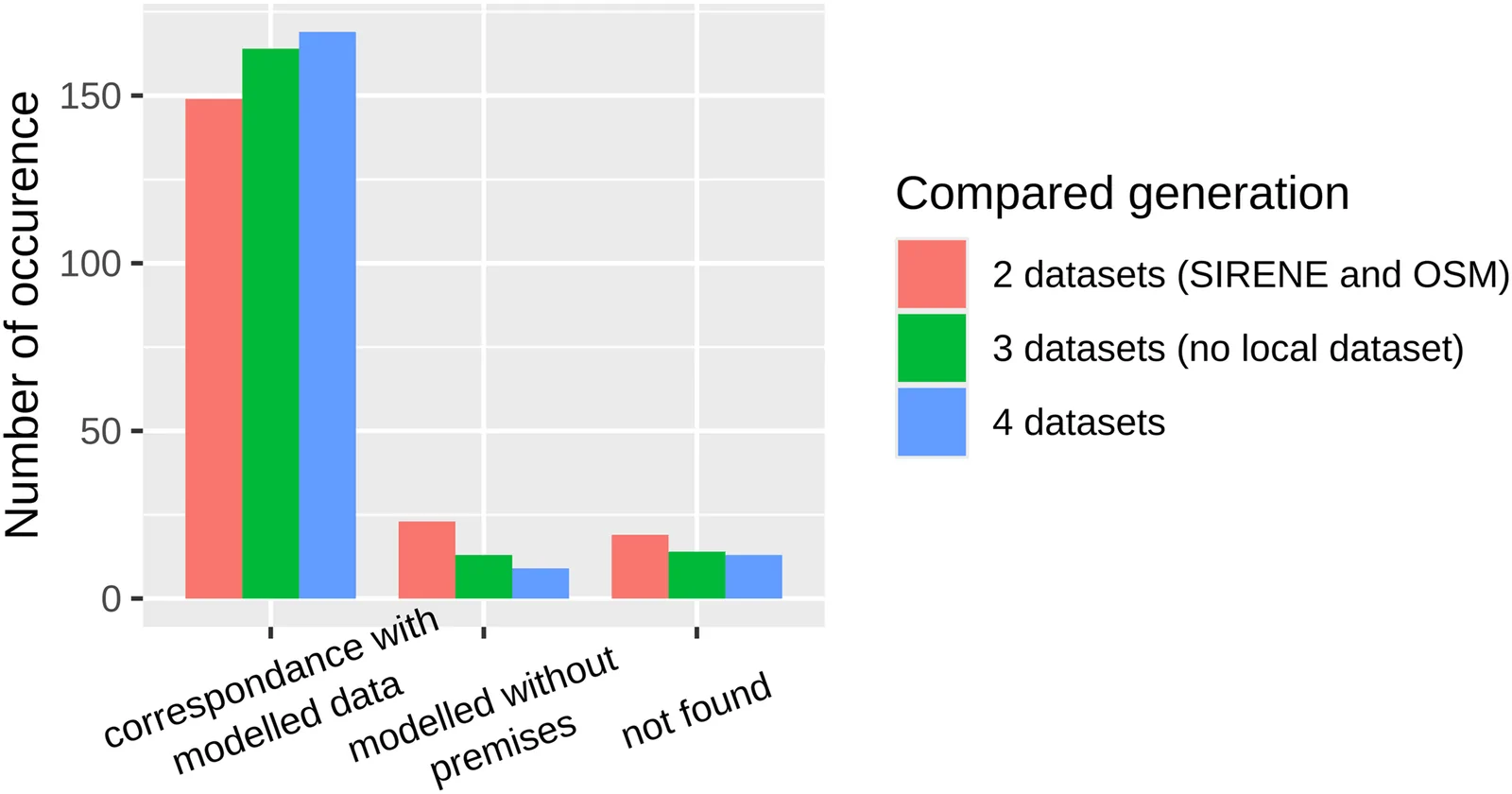
Correspondence of the observed activities with the modelled activities.

Fig 31 presents this matching from the opposite perspective, showing which modelled activities with a premises have a correspondence in the observed dataset. A large number of activities are therefore modelled without a matching observed activity. It is important to note that if an activity isn’t open to the public, it may exist but have no sign visible from the street. This can explain the very high count in this category, which is even greater than the number of matched activities. It is also noticeable that using fewer datasets produces fewer unmatched activities that are open to the public, but more unmatched activities that are closed to the public. Only a few errors are found where a modelled activity should have been merged with another activity, although such cases are a minority.

**Fig 31:**
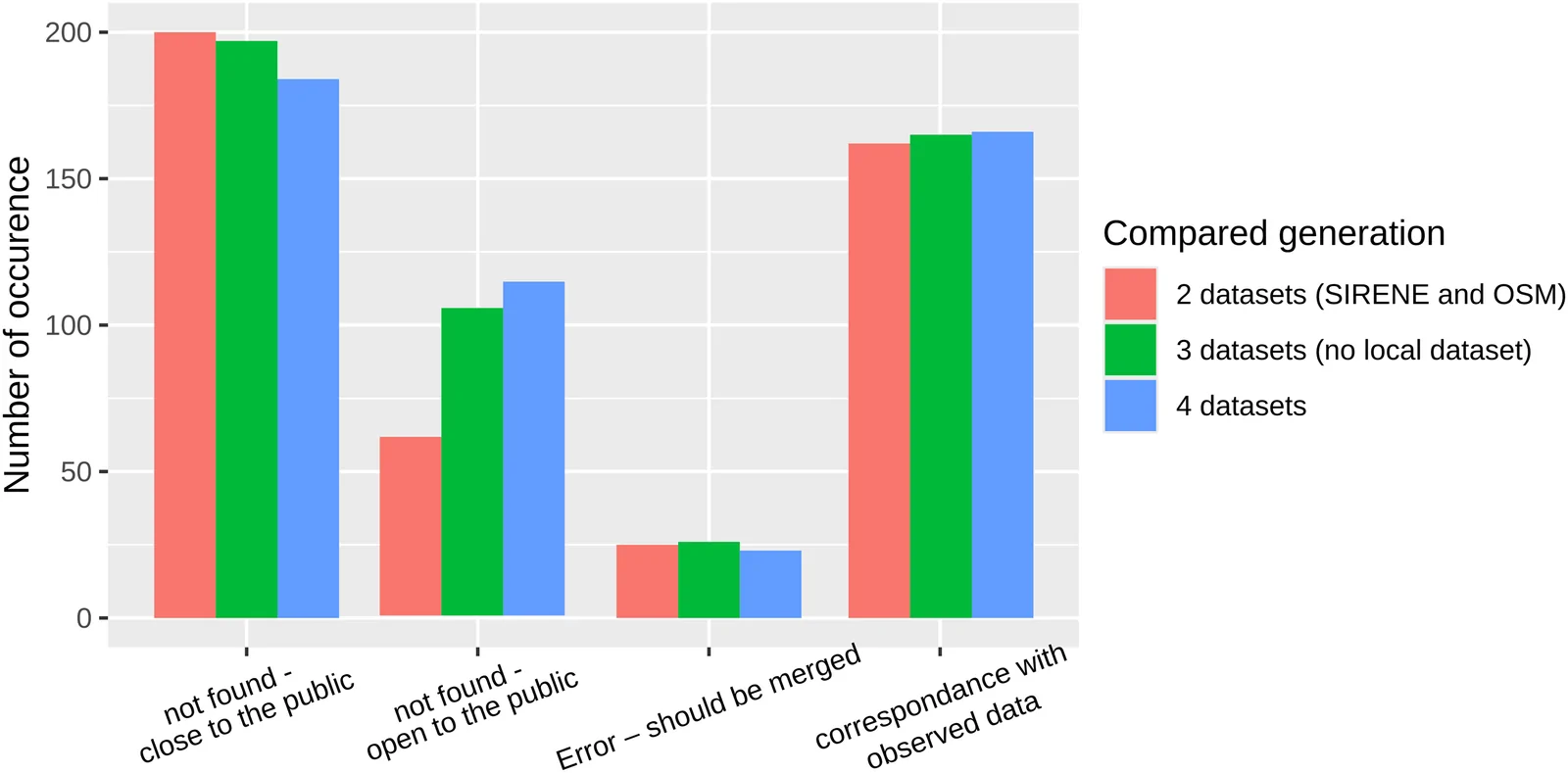
Correspondence of the modelled activities with the observed activities.

The premises presence score, resulting from the multiple calculations described in 3.3.1, is analysed for the unmatched activities. If this score is greater than 1, a premises is set in each generation. If the score is lower than 1, a Bernoulli trial is performed using the score as the probability parameter. The score differs markedly between the three generations because it depends on the number of merged datasets. The large number of activities compared should yield results that are less biased by randomness.

Fig 32 shows which premises of unmatched activities were determined via probabilistic sampling and which were assigned deterministically in each generation. The setting of non openness to the public of unmatched activities is often decided stochastically. For activities that are open to the public, the assignment is less probabilistic than the activities close to the public. Generations that use only two datasets also exhibit greater uncertainty. When an activity that is open to the public has a real-world counterpart, its premises presence score is frequently higher than 1. Consequently, the premises presence probabilities should be reduced and the penalties applied to the similarity scores should be increased, especially for activities that are open to the public.

**Fig 32:**
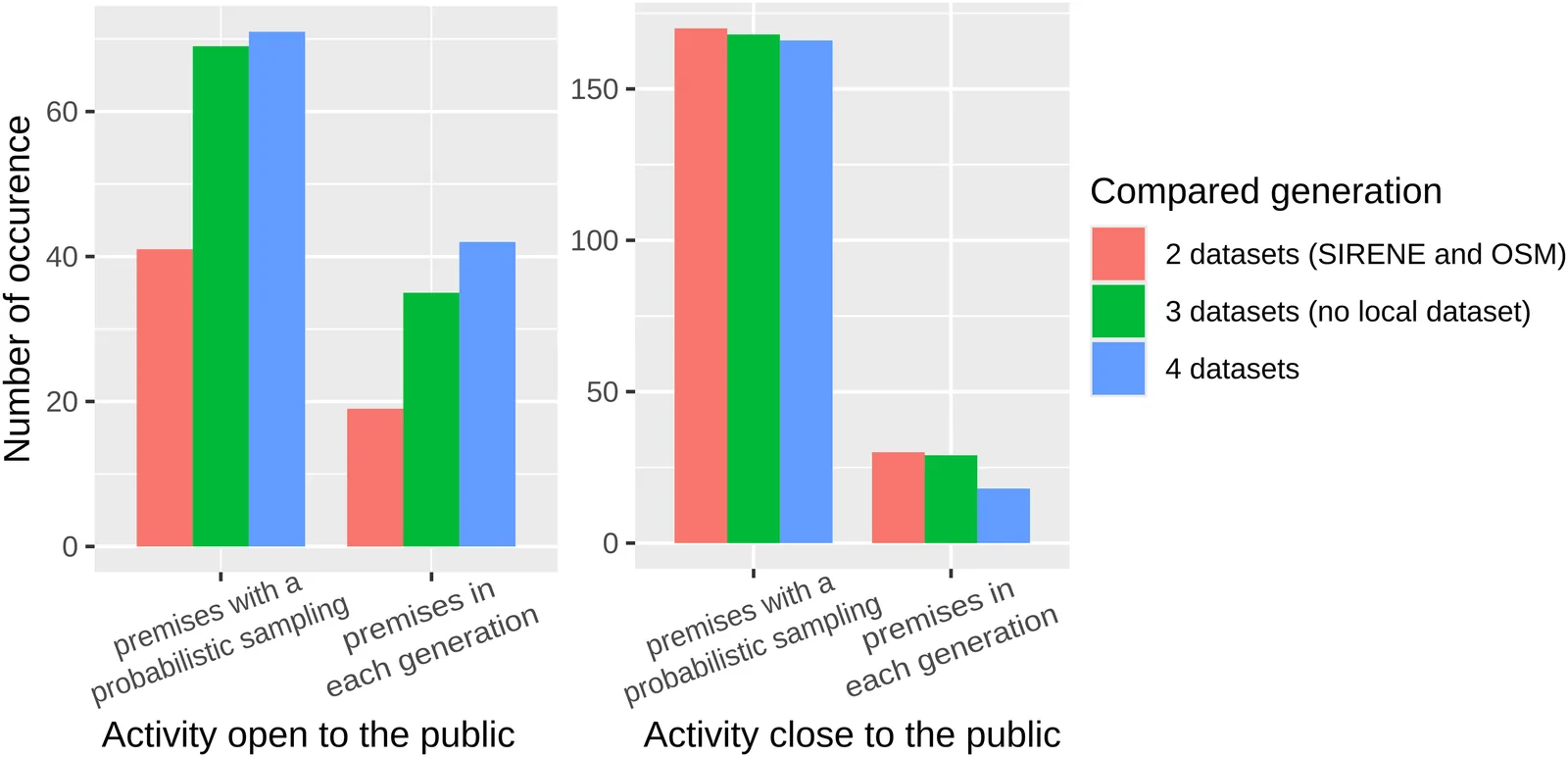
Unmatched activities by their premises presence score.

**Attributes** Particular generated activity attributes are analysed. A manual label is set in order to assess the correspondence between observed and modelled data. As the two datasets that provide names are used in every generation, the results are almost identical for the three generations, and only one is shown in Fig 33. Types are a fundamental element used in the process of matching observed and modelled activities; consequently, it is rare for them to differ. Names of matched activities are often correct, but a noticeably larger proportion of them differ.

**Fig 33:**
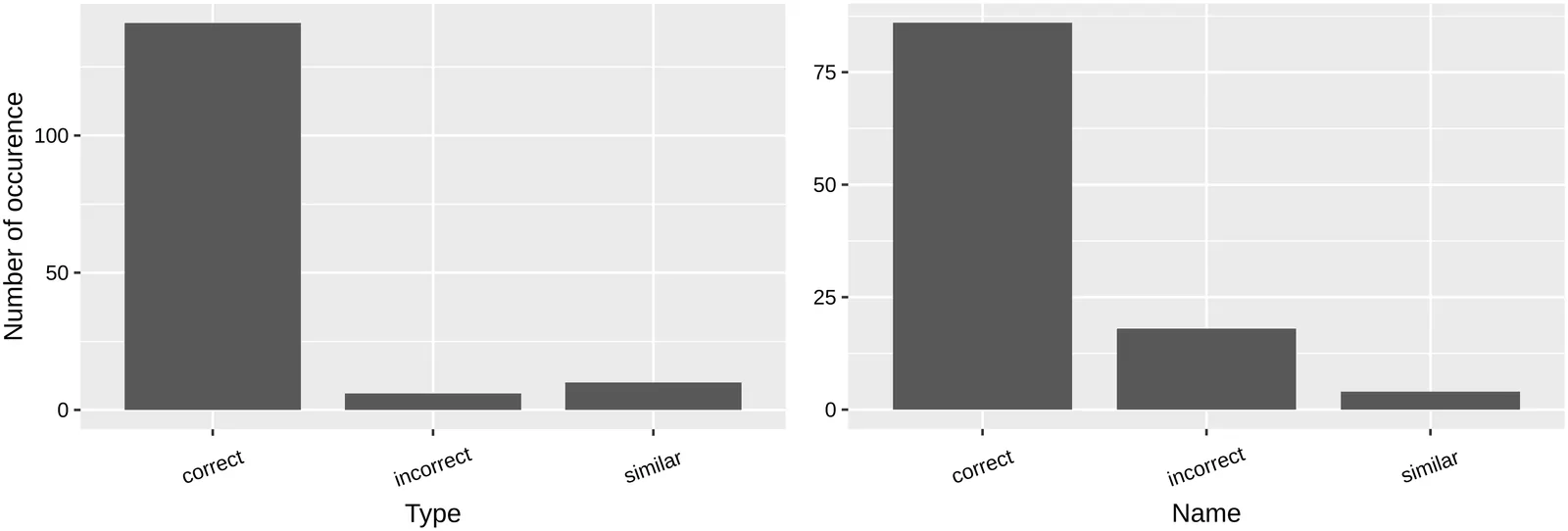
Comparison between names and types of modelled activities compared to observed activities.

**Opening hours** Many activities display opening hours and days on their façade. The comparison between such observed information and the modelled opening hours, as described in 3.3.3, can be divided into two analyses. If a value is provided by the OSM dataset, it will be present in every model generation and the comparison will only evaluate the accuracy of this VGI database. If the value is synthetically generated, it is very unlikely to correspond exactly to the collected value. A distance assessment is therefore applied to characterise the discrepancy in opening hours. If the hours are not identical but are shifted by up to 1.5 hours, the label *similar* is assigned.

Fig 34 shows these assessments. Few OSM values are dissimilar to the observed data, mainly because of insufficient precision regarding opening times. Synthetic opening hours have no exact times, but most are classified as similar; the days are therefore often incorrect. The error mainly stems from weekend opening times.

**Fig 34:**
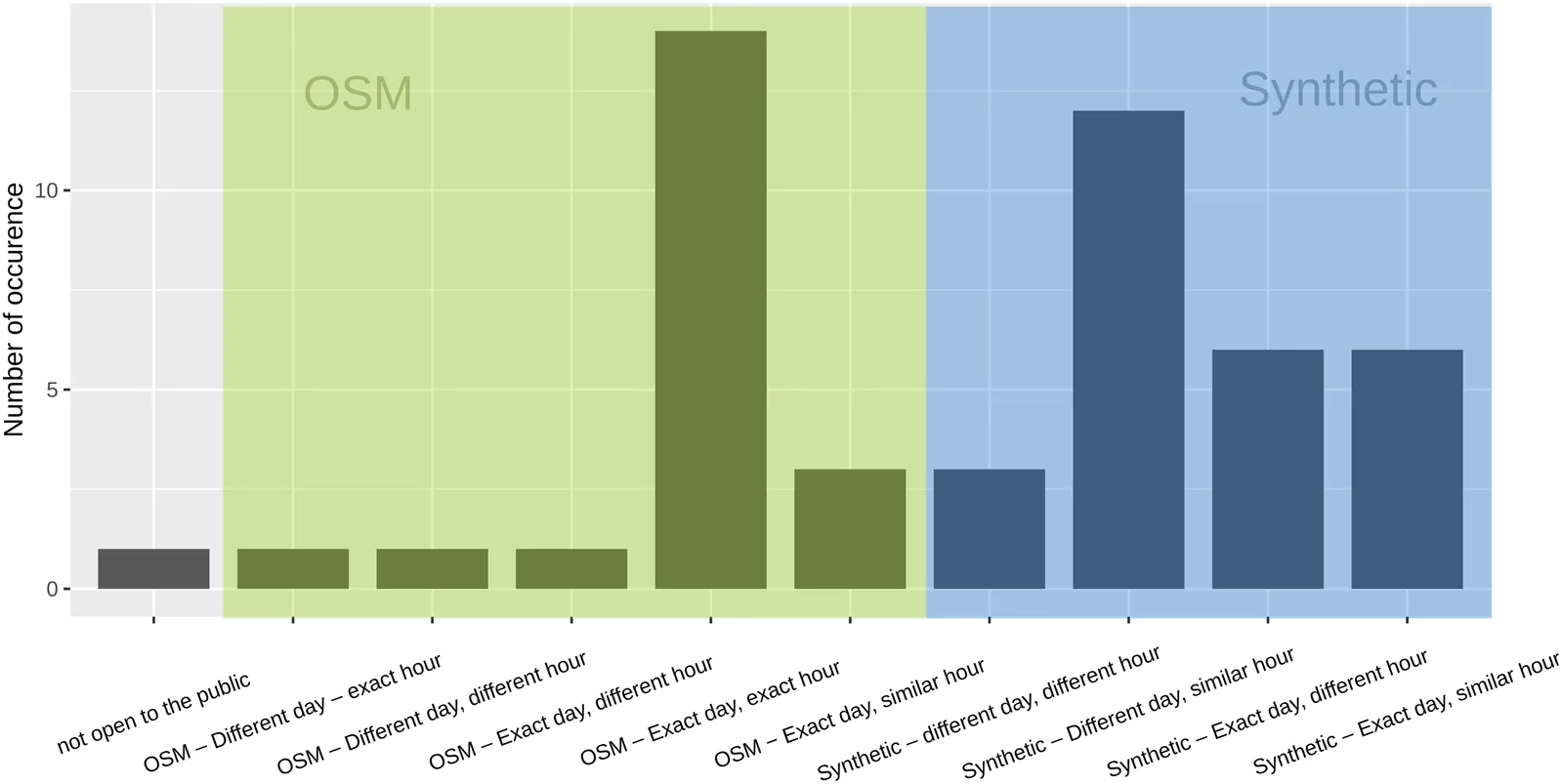
Opening hours of modelled activities compared to observed activities.

The non-openness to the public of activities that are supposed to be open is also reported in this figure, but this information is mostly correctly set. A model improvement is then required to increase the accuracy of opening-hour behaviour on weekend days. Statistical models could also be trained to infer more precise opening hours with the use of location and type of activities.

This experiment shows the strengths and weaknesses of the model. Almost every observed activities had a correct type and location correspondence, but many modelled activities wrongly occupy premises. A quick comparison with street-view images of the studied area shows that the latter information is often not up to date. This model offers the advantage of quick and automatable updating. The use of many datasets appears beneficial for the model’s results. More modelled activities had a correspondence in the observations, and they are proportionally less impacted by randomness. Nevertheless, adapting diverse typologies to the model is required, as stated in Section 4.2.

Because too much activities with premises were modelled, tuning the premises presence parameters presented in 3.3 is necessary. This ground truth dataset now exists and could be used to evaluate future model improvements. The historicisation of the generated data should allow comparisons at corresponding time periods. If the gathered sample were larger, it would be possible to calibrate the numerous arbitrarily set probability scores. If the sample were much larger, a deep-learning model could be trained.

## 5 Systematic application of the model

This model has been systematically applied to metropolitan French communes. The results have been pre-calculated and stored in a PostGIS database. Two distinct dates have been processed:

- 2019-12-21, which corresponds to one of the first versions of the *BD_Topo*® 3.0. Every statistical data was published for this date, making it the most complete and consistent dataset.
- 2025-02-01, which is the latest release at the time these lines were written. Not all data sources have released datasets for this period, particularly sociodemographic census values, as well as the authoritative BPE and local datasets (both from 2023). These datasets are therefore updated using the dedicated process, which may still produce inconsistencies.

This model has also been deployed at a large scale, covering all 34 883 communes of metropolitan France as of December 21, 2019. It generated 22 189 112 building groups, 34 512 585 housing units, and 9 694 211 activities. The generated activities conflated 12 165 330 initial activity records, of which 4 238 050 occupy a premises and host 27 983 474 jobs. Building floor area was distributed across 4 368 368 000*m*^2^ dedicated to housing and 2 431 019 500*m*^2^ for activities. Such values align with national statistics. The maps in Fig 35 represent the density of different types of activities in Paris. Some well-known spatial patterns can be verified: Shops and leisure activities are more concentrated in the centre of Paris on the right bank of the Seine and in the *quartier latin*. Administrative institutions and offices are more concentrated at the western part of the city. Health and education facilities are distributed rather uniformly.

**Fig 35:**
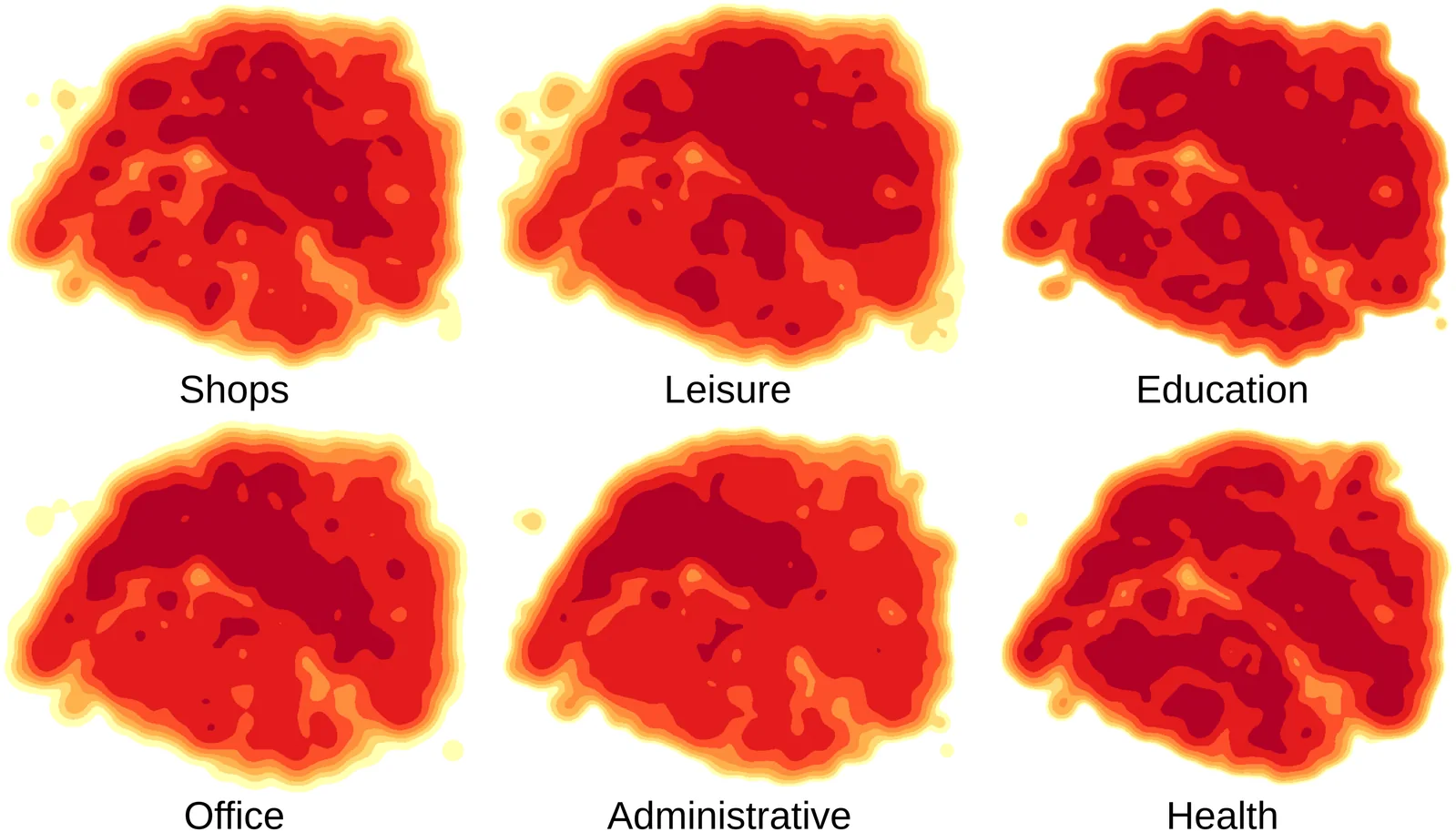
Density of activities in Paris disaggregated by *general types.*

## 6 Discussion

The model presented in this article is reproducible, well-detailed and its implementation is available under a free, open source licence. Therefore, the list of improvements, repurposing, and reuse discussed in this section could be undertaken by any interested actor. This model is part of the ICI project [14,15] and will benefit from continuous improvements by the authors of this article.

Multiple module improvements have been outlined during the model presentation and its validation steps. Extending the collection of ground truth data would allow calibration of the numerous parameters that have been set manually (probabilities of premises, distance thresholds, etc.). The next step for this model is to work on an ontology, by extending existing activity type and building ontologies. The generated data would then be deployed on a triple store to enable use on the Semantic Web and to share its results on a large, modular scale. The inclusion of this model in the National Digital Twin of France is also an ongoing process.

This model has been applied to all French communes and has, for now, only been used with French data. Applying this model to another national context would be a solid proof of its portability and robustness to input data. A minimal example using Eurostat data would enable this model to be applied to each European country.

It is possible to enhance the model on specific subsystems to comply with given simulation purposes. For example, dedicated plugins for generating kindergartens, schools and universities have been developed for the ICI project, using specific authoritative datasets.

Due to its modular design, different parts of this model can be reused in other works. The automatic activity-conflation process itself could be used as a standalone project to conflate diverse sources of activities or POIs. The whole pipeline could be employed in contexts other than modelling building land use, as highlighted in this article: base data for spatiotemporal analysis of retail, office vacancy or real estate, creation of to-do lists for collaborative cartographic projects such as OSM, etc. This model would also be suitable for the generation of realistic synthetic populations.

The descriptive data produced by this model could also populate 3D building models. Modelling the inner organisation of buildings has become a subject of interest [6]. Usually, those techniques focus on the physical configuration of interiors rather than on the use of such rooms [51]. Knowing the function of a premises could still be of great interest for constructing its 3D model. Parametric algorithms are used by [52] to create a grammar of indoor architecture and generate room configurations for each floor of a building. Procedural simulation could be used to model a connected set of rooms forming a single activity or housing unit. The systematic deployment of these models could benefit from the knowledge produced by BLU.

Various city models (CIM, CityGML with the IndoorGML extension) or building models (BIM) are also challenging research and industrial fields [53]. The data generated by this model could fit into such formalisms, as multiple research projects try to link GIS with BIM models [54–56]. Generated data could also be used to assist façade reconstruction algorithms [57] by providing realistic semantic information about the distinct activity types in a building.

## 7 Conclusion

The model presented in this article generates a precise and well-detailed building land use at an individual scale. This building land use is divided into housing units and activities, each accompanied by rich descriptions, either extracted from datasets or simulated. Data are generated for the specified dates through the integration of historicised data. It can adapt to various input datasets and can be deployed in different geographical environments, ranging from sparsely described to feature-rich datasets. Various validation methods are applied to the numerous original methods of each module in order to evaluate the realism of simulated attributes, assess the benefits of more or less complex input datasets, and validate the overall approach. Ongoing work consist in deploying the model to other European country using harmonised Eurostat datasets, and subsequently to sparser-data contexts. Our approach represents a novel way to model building land use. Whereas much prior work has used a combination of image analysis and social data to infer broad classes of building land use, sometimes combining such classes, this model produces individual results with an unrestricted number of activity-type combinations. The results produced are highly descriptive and could be exploited through a database or via the semantic web in the near future. This model also brings significant contributions to the literature. The building object itself is questioned, whereas it is taken as given in other works. A new concept suitable for building land use definition is modelled, which does not rely on a building’s geometric shape. The conflation of activities is a novel systematic approach, flexible regarding the number of input datasets. Our findings suggest that the more datasets are merged, the more precise the results become. The conflation methods could be reused in the POI research field. While the activities’ attribute enrichment is tailored to generate diverse basic and useful information, the housing area attribution mechanism highlights a need for further research to address this issue more effectively. Finally, original validation methods have been designed and could be transposed to other similar works. Ground truth data collection has ultimately confronted the modelled data with the real world and provides a deep understanding of the weaknesses of our approach, whereas similar studies limited their validation processes to the use of online street-view images. Efforts have been made to ensure the transparency, availability and reproducibility of all resources used in this work. Annex B to D provide descriptions of the datasets used in this work, direct links to download them, and programs to import them into a PostgreSQL database. Annex E describes the pseudocode of the algorithms developed in this work. All implementations are available in a Java program hosted under a free and open source licence at this address: https://gitlab.inria.fr/ici/ici_buildingactivities. The large generated datasets will soon be made publicly available and are already in use by the ICI epidemiological simulator [14,15]. Finally, to give the reader a sense of the generated dataset, a navigable sample can be explored at this address: https://ici.saclay.inria.fr/SampleBLU.html.

## Appendix

### A Glossary

Specific notions used in this article are defined as follows.

- **Building land use**: An inventory of the distinct functional spaces hosted by a building. Several classes of attributes can enrich this description: type-related attributes characterise functional spaces, visitor access, and occupancy levels, while geographic attributes describe how functional spaces are distributed within a building (e.g., number of storeys, floor area).
- **Building block**: A primitive building element for the building model. Most of the time, it is based on a simple geometry and any rupture (different height, physical separation) would consist in the creation of a new *building block*.
- **Building group**: *Building* model that allows a functional continuity in the hosted activities or housing units. Consists in grouping one or more *building blocks* together. It is the main building object handled by this model.
- **Functional space**: A primitive element of the building’s floor, related to a use. Could be one or more connected rooms, but the model presented in this article doesn’t work at this level of detail. In this model, functional spaces can be of two types: *housing units* or *activity*.
- **Housing unit**: Set of rooms dedicated to accommodation for one or more persons. A household can settle in a housing unit, but it can also stay empty. A dedicated *premises* is automatically attributed to a housing unit.
- **Activity**: A formal or informal occupation which is linked to a particular building for spatial or administrative reasons. It does not necessarily occupy a *premises*. Its type can be composed of *typologies*.
- **Premises**: Space dedicated to a *functional space* inside a building. As a housing unit automatically own a dedicated premises, this term is not used to describe such element. In this model, it cannot be shared between two functional spaces.
- **Typology**: List of classes which partition different kinds of activities from a single dataset. Two kinds of typologies are considered: (i) **project typologies** which are attached to each dataset and (ii) the **common typology**, which is created specially for this model. Each class of *project typologies* must match a class from the *common typology*. Annex D.2.1 detail the creation of the *common typology*.
- **Historicised**: The ability to recreate the modellised object at the precise state it had at given date, independently from the date of release of the data.
- **Statistical aggregate**: Represent the lowest scale of spatial unit for which statistics are public. Typically groups a few thousand inhabitants. Corresponds to Census Tracts in USA or IRIS in France.

### B Reproducibility

All processes undertaken in this work are from open source libraries and use only open data. This work should therefore be fully reproducible. Links to corresponding executable code were provided in each validation process description. Every process has run on a Java Virtual Machine of reference *openjdk 23.0.1 2024-10-15*, hosted by a personal computer with LMDE 6 (faye) with Intel(R) Core(TM) i5-10310U CPU @ 1.70GHz and 16GB of RAM. Multiple datasets are stored on a PostGresSQL 15.10 database and PostGIS 3.3.2. Few Java classes are used to automatically load data gathered from the Internet to a locally stored database, as summarised in Table 5. Nevertheless, every classes could be run without any database, by providing paths to the corresponding GeoPackages.

**Table 5:** Data gathering for each process in this paper (links are in hypertext).

Data	Download	Java loading class
Statistical Aggregate (IRIS)	IGNIRIS-GE and INSEE infra-community census on housing)	Master ICI project
Building	IGN BD_Topo®	BuildingLandUse
Address	OpenData archive	BuildingLandUse
Parcel	OpenData archive	BuildingLandUse
Sirene	INSEE	BuildingLandUse
OSM	Geofabrik.de (see C).	BuildingLandUse

### C OSM import

A raw dump of the targeted zone of OpenStreetMap is stored inside a PostGIS database, using the program *osm2pgsql*. In order to import only the required features, the *flex* mode of osm2pgsql is used, which gives greater control over the import options and the way data are stored. The configuration file in *lua* language can be found here: https://s.42l.fr/confOsm2Pg. Nodes or ways that have one of the tags *amenity*, *shop*, *tourism* or *office* are kept and transformed into a point in the centre of the building if necessary. A large list of values is filtered in order to keep only the point representing building’s activities. Table 6 summarises attributes selection.

**Table 6:** Filtered types of OSM data.

Feature type	Amenity	Shop	Tourism	Office
Number of distinct types	644	1 106	104	365
Number of type occurrence	482 168	354 727	54 709	79 375
Approximate number of filtered types	148	1	29	0

There are overlaps between the feature types. Amenity and shop values are both tagged for 1.11% of the features, amenity and tourism 0.31%, amenity and office 0.09%, shop and tourism 0.03%, shop and office 0.05%, tourism and office 0.003%. Triple overlaps are insignificant.

### D Input data

French data are analysed in this section in order to give the reader the reasons which has lead to their choice.

#### D.1 Building generation.

Table 7 summarises the diversity of available data in France through different time periods and their characteristics.

**Table 7:** Data characteristics.

Building	Producer	Year	Periodicity	Geometry type	Use	Height	Storeys	Dwellings
BDTOPO v2.0	IGN	2008-2011	yearly	3D	◻	∎	◻	◻
BDTOPO v2.1	IGN	2012-2016	yearly	3D	◻	∎	◻	◻
BDTOPO v2.2	IGN	2017-2018	yearly	3D	◻	∎	◻	◻
BDTOPO v3.0	IGN	2019-2022/09	quarterly	3D	∎	∎	∎	∎
BDTOPO v3.3	IGN	2022/12-2024/03	quarterly	3D	∎	∎	∎	∎
BDTOPO v3.4	IGN	2024/06-present	quarterly	3D	∎	∎	∎	∎
**Address**	Producer	Year	Periodicity	Geometry type	Full name			
BAN	Consortium	2019/07-present	monthly	points for entrances and parcels	∎			
BAN Plus	Consortium-IGN	2022/12-present	quarterly	points and links from addresses to buildings and parcels	∎			
Adresse Premium	IGN	2014-2022	yearly	links from addresses to buildings and parcels	∎			
BDADRESSE	IGN	2008-2022	yearly	points	∎			
Cadastral parcel address	DGFIP	2018-present	yearly	points for entrances and parcels	∎			
**Parcel**	Producer	Year	Periodicity	Geometry type	Full name			
Cadastral parcel	DGFIP			2D polygon	◻			
BDPARCELLAIRE	IGN	2007-2019		2D polygon	◻			
Parcellaire Express (PCI)	IGN	2019-		2D polygon	◻			

**Building:** In the French context, four main sources coexist for building data: the *BD_Topo*®, produced by the French national mapping agency (IGN), buildings from the OSM project and local government databases managing geographical data. A fourth data source consist in an authoritative database for building, which is currently being developed by trans-governmental agencies on the BatID project. The BatID project is merging multiple datasets concerning buildings, such as their energy efficiency, their construction material, from 25 different data producers. It also aims to assign a unique ID to every building. It currently faces some difficulties due to the multiple definitions that the building notion can cover, the geometric entanglements with parcels and addresses, and the cardinality of each of those objects. It also misses public-owned buildings, which is of interest for many reasons.

The *BD_Topo*® is a stable building database, available countrywide and edited by IGN. This building database should be precise enough concerning geometries (1m precision) and its attributes (including height, number of storeys, and number of housing units) cover the general information produced by other authoritative data producers. OSM buildings have been judged to be less precise by other studies [31], and incompleteness has also been detected [58]. Local databases, maintained by cities or higher administrative actors, could provide manual or hand-corrected data, with high definition buildings. They could be used later to enrich this building model. *BD_Topo*® produces a new set of data each year from 2008 to 2018 then four releases per year, which approximately covers the project time-lapses needs.

**Addresses:** In France, multiple administrations had parallel databases of postal address. IGN had a component within *BD_Topo*® with addresses from 2014 to 2022, called *Adresse_Premium*® and providing links from addresses to cadastral parcels and buildings. The standardisation delegation task force created the BAN (*base d’adresse nationale*), which must merge every data from every administration dealing with postal addresses and allowing regular updates by local urban bureaux. On the first version of the BAN, entrances addresses, created by postal services, were linked with cadastral parcel addresses, attributed by tax services. This represents a suitable topological simplification for the integration of parcel data. On the new version of the BAN (since 2022), namely BAN-Plus, this distinction is not included but a link is automatically generated between addresses and buildings from the *BD_Topo*®, as a replacement for the discontinuation of *Adresse_Premium*®. For dates with no existing links, or to reapply this model to another country, a simple process to assign addresses to buildings was designed

**Cadastral parcels:** In France, two different institutes produce parcel data. The *Cadastre* is edited by the French Tax Service (DGFIP) and produces a closed-source dataset with address information but also with inconsistencies and topological errors. IGN derives this dataset to an open product which fixes topological issues, but does not contain address information. Address and parcels can be linked in an open dataset in other countries where parcels were defined with simpler relationships to other geographical elements. If it is not the case, a random address from an entrance contained by the parcel could be attributed.

#### D.2 Activity.

For the French case, two authoritative data sources are used, both from the French National Institute of Statistics and Economic Studies (INSEE):

- **SIRENE**: Administrative database for every declared companies or associations. Every legal unit is linked to one or more establishments, which are spatially localised. This database has many descriptive and historicised fields (names, bounds on the number of workers, legal unit) and receives monthly updates.
- **BPE**: Aggregates public equipment data. This dataset uses many sources, including SIRENE, but also from ministries (health, national education, etc.), public agencies (*La Poste*, National Company of the French Railways (SNCF), etc.)(see https://www.insee.fr/fr/metadonnees/source/operation/s1524/processus-statistique) Each year, a new dataset is produced. Its quality is validated by INSEE teams (see https://www.insee.fr/fr/metadonnees/source/operation/s1524/gestion-qualite), which cause a publication delay of 18 months.

The **OSM** project has been used in numerous research projects due to its global accessibility, large contributor base and rapid evolution. There are different ways of using OSM data, an API for accessing raw content (e.g., https://overpass-turbo.eu/), portals that sort and transform data (e.g., https://www.geofabrik.de/). As a free and open project, it is possible to host all necessary information in a PostGIS database using the *osm2pgsql* (https://osm2pgsql.org/) tool. Datasets managed with this tool are updatable, highly configurable. This tool also provides very fast access once the data are stored. All data that could represent indoor activities are kept, using a filter on the tags *amenity*, *shop*, *office* or *tourism*. Every value of those tags which represents outside activities or micro-mapping elements (such as watering point, garbage, etc.) are filtered out. Annex C describes those importing and sorting operations. Multiple attribute can be extracted from this data, such as opening hours or capacity of activities. This information is strongly related to the type of the activity (for a restaurant, it means the number of seating places). The historicisation of this data is possible through an API which tracks information about modifications applied to each object.

In the Paris case, a dataset from the **local** source *Atelier Parisien de l’URbanisme* (APUR) is added. This dataset has the particularity of manual surveys and reviews of shops and social facilities in the city of Paris. This does not concern other activities such as offices or other public services. New datasets are produced every three years. An attribute giving an indication about the size occupied by each activity is also provided. It also contains vacant premises, which are absent from other databases.

Table 8 describes a summary of each data source's activities used in this work, while Table 9 describes the feature availability for each of those sources.

**Table 8:** Summary of activities for each data sources.

Dataset (type)	SIRENE (authoritative)	OSM (VGI)	APUR (local)	BPE (authoritative)
Date of the dataset	2025−02	2025-02-01	2020	2019
Number of occurrences in Paris	3 057 172 (1 119 960 currently open)	55 966	83 720	135 306
Number of occurrences in France	35 779 547 (14 194 881 currently open)	936 412	/	2 471 192
Number of typology classes	four successive typologies with respectively 655, 744, 723 and 1 617	1 781	218	188

**Table 9:** Attribute enrichment from multiple sources to the merged activities, with the percentage of presence in France.

Datasource	Sirene (Authoritative)	OSM (VGI)	APUR (Local)	BPE (Authoritative)	Merging method
Name	from establishment or legal unit (60.8%)	given name (87.9%)	/	/	append
Type	(100%)	(100%)	(100%)	(100%)	append for multi-source conflation, replace for self conflation and update
Address	(100%)	(100%)	(100%)	(0%)	Replace, authoritative data is prioritised
Other attribute	workforce (6.7%, but 13.9% of the currently open establishments)	Opening hours (27.0%)	Approximate area (100%)	/	add workforce, random opening hours, higher area approximation
Opening during restriction	Right to open during lockdown (100%)	Effectively open during lockdown (1.4%)	/	/	Replace, effectively open is prioritised for multi-source conflation, opening = true is prioritised for self conflation

D.2.1 Activity typology: The *common typology* used in this article was developed in order to be used by the epidemiological spreading simulator ICI [14,15]. Classes are constructed to group activities that can be collectively restricted by epidemiological policies (Table 9). Default attributes are set if project typologies does not fulfil all criteria and are presented in Table 10. Four pieces of complementary information are added to describe activities:

- **hasPremises** is a probability estimating the chances that such activities occupies a premises or not.
- **isOpenToPublic** is a probability that the type of activity is freely open to any kind of public.
- **Profile of opening hours** that are attached to each activity of this type and which is open to the public. Those hours can be slightly adjusted randomly (the opening and closing time could be slightly translated, hours can be open at night and on weekends, lunch break may be added or omitted). If this information is present in a matched VGI activity, the real information will be used.
- **typical visit duration** represents a typical duration of an individual stay inside the activity.

**Table 10:** Common typology of the ICI project, used in this article.

typologyName	typicalHours	typicalStayDuration	isOpenToPublic	hasLocal
commerce.achatDivers	tu-sa 09:00–18:30	00:15	toujours	probable
commerce.alimentation	mo-sa 07:00–20:00 su 8:00–12:30	00:30	toujours	trèsProbable
commerce.serviceDeplacement Individuel	mo-sa 09:00–18:00	00:30	probable	aléatoire
commerce.servicePersonnel	tu-sa 09:00–18:00	01:00	toujours	probable
demarche.accompagnementSocial	mo-sa 09:00–18:00	02:00	aléatoire	trèsProbable
demarche.administratif	mo-fr 8:30–17:30	01:00	aléatoire	toujours
demarche.affairePersonnelle	mo-sa 09:00–18:00	01:00	trèsProbable	probable
demarche.hebergement	mo-su 09:00–11:30 14:00–19:30	01:30	trèsPeuProbable	toujours
demarche.hebergement.ephad	mo-su 09:00–11:30 14:00–16:30	01:30	peuProbable	toujours
education.particuliere	mo-sa 17:00–21:00	02:00	trèsProbable	probable
education.primary	/	/	jamais	toujours
education.elementary	/	/	jamais	toujours
education.midschool	/	/	jamais	toujours
education.preschool	/	/	jamais	toujours
education.highschool	/	/	jamais	toujours
education.highschoolpro	/	/	jamais	toujours
education.secondary	/	/	jamais	toujours
education.superieure	/	/	jamais	toujours
industrie.secteurPrimaire	/	/	peuProbable	aléatoire
industrie.secteurSecondaire	/	/	trèsPeuProbable	trèsProbable
industrie.secteursecondaire.artisan	/	/	probable	probable
industrie.secteurTertiaire	mo-sa 8:00–19:00	00:30	aléatoire	probable
industrie.transport	/	/	trèsPeuProbable	toujours
industrie	/	/	trèsPeuProbable	trèsProbable
loisir.activiteCulturo-sportif	mo-sa 16:00–21:00 su 8:00–12:30	01:30	toujours	aléatoire
loisir.activiteCulturo-sportif.conservatoire	mo-fri 17:00–20:00 su 09:00–18:00	02:00	toujours	toujours
loisir.activiteCulturo-sportif.religion	fr-su 11:00–18:00	01:00	toujours	aléatoire
loisir.hebergement	mo-su 14:00–21:00	12:00	toujours	toujours
loisir.hebergement.camping	mo-su 14:00–21:00	12:00	toujours	toujours
loisir.hebergement.hotel	mo-su 14:00–21:00	12:00	toujours	toujours
loisir.restauration	mo-sa 11:30–14:00 18:00–23:00; su 11:30–14:30	02:00	toujours	trèsProbable
loisir.sortie	mo-sa 09:00–20:00	1:30	toujours	trèsProbable
loisir.sortie.cinema	th-su 18:00–23:00	02:00	toujours	toujours
loisir.sortie.competitionSportive	th-su 18:00–23:00	02:00	toujours	trèsProbable
loisir.sortie.concert	we-sa 19:00–23:30	02:00	toujours	toujours
loisir.sortie.discotheque	th-su 22:00–05:00	04:00	toujours	toujours
loisir.sortie.musee	we-mo 09:30–19:30	01:30	toujours	toujours
loisir.sortie.salleJeu	mo-sa 09:00–23:00	02:00	toujours	toujours
loisir.sortie.zoo	we-mo 09:30–19:30	03:00	toujours	toujours
mobilierUrbain	24/7	00:10	toujours	peuProbable
sante.assistance	mo-su 08:00–19:00	00:30	trèsPeuProbable	peuProbable
sante.bien-etre	mo-fr 8:30–17:30	01:00	toujours	probable
sante.commerceMedical	mo-sa 09:00–18:00	00:15	toujours	toujours
sante.hopsitaliere	24/7	02:00	toujours	toujours
sante.medecineDeVille	mo-sa 8:00–19:00	00:30	toujours	toujours
sante.specialiste	mo-fr 8:30–17:30	00:30	toujours	toujours
transport.infrastructure	mo-su 06:00–23:30	00:10	toujours	toujours
transport.personnel	24/7	00:30	toujours	probable
vacant	/	/	jamais	toujours

Every typology from the processed dataset are stored in a *.csv* file at this address: https://s.42l.fr/typo

### E Algorithms pseudocode.

**Algorithm 1: Generation of the number of housing units in buildings, whether their this information is incomplete or unknown (see 3.1.2 page 11). A Java implementation available here: https://s.42l.fr/HUgen**



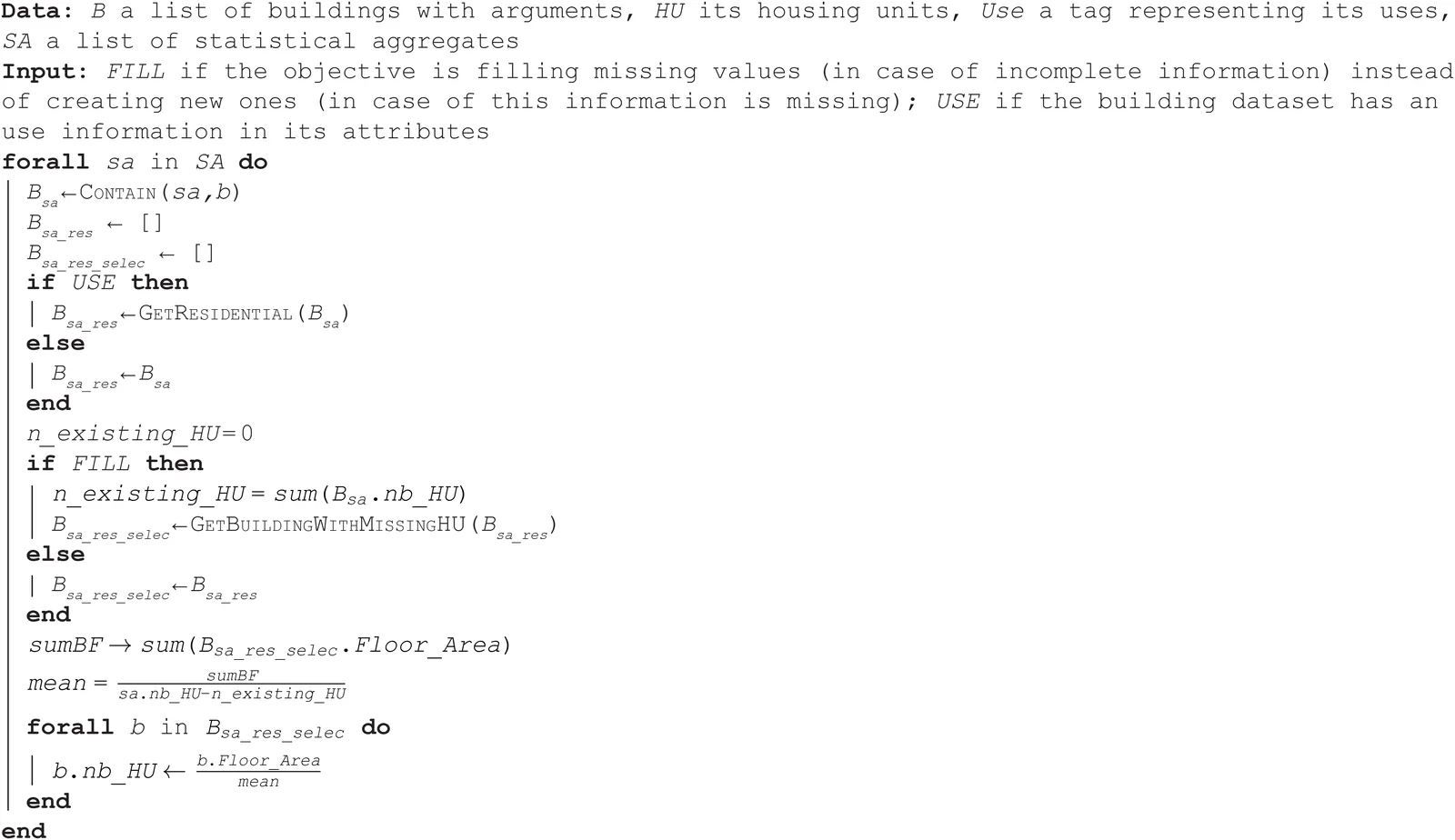



**Algorithm 2: Formation of *building group* (see 3.1.3 page 11) (Fig 36). A Java implementation available here: https://s.42l.fr/buildingGroup**



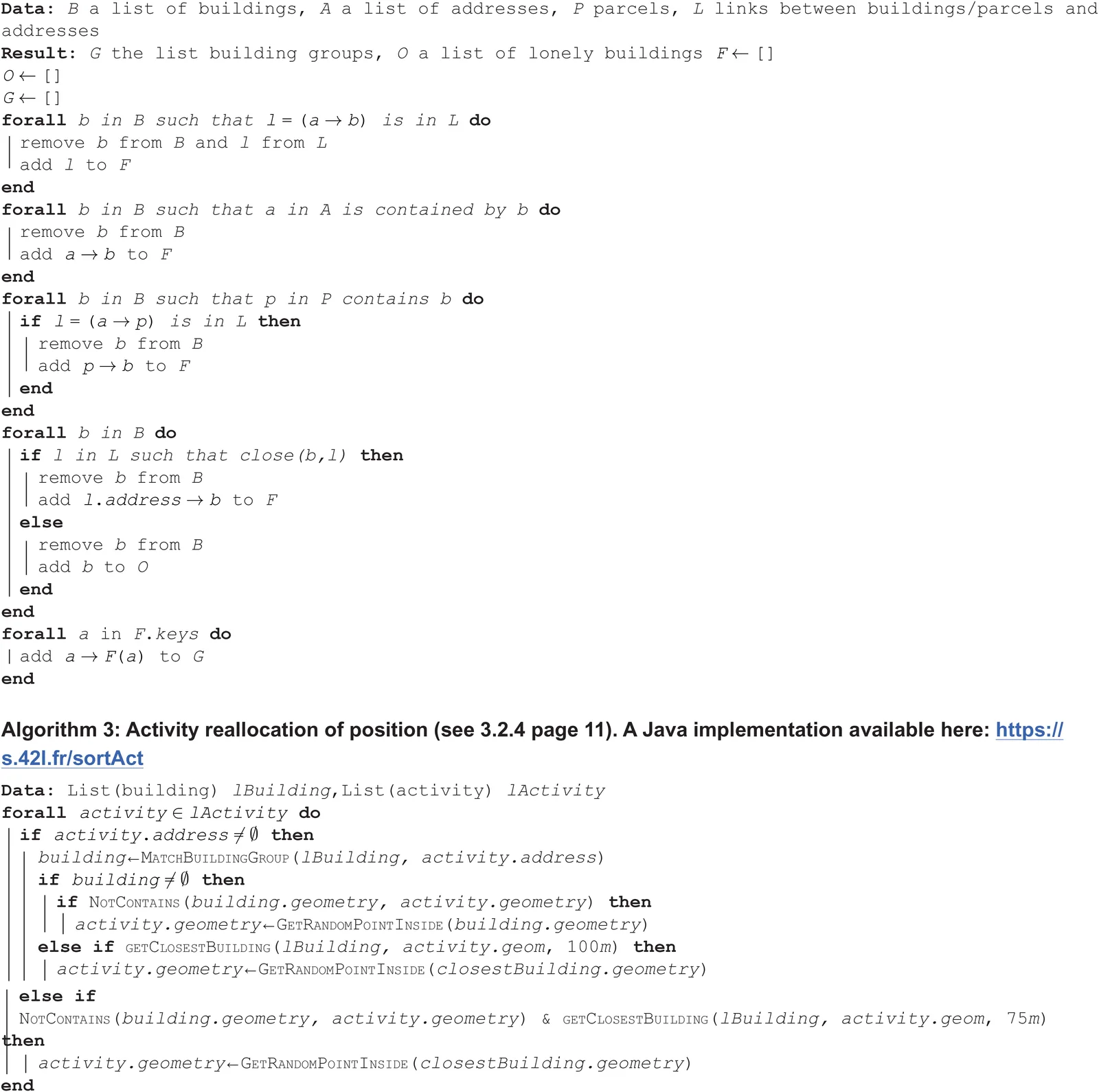



**Algorithm 3: Activity reallocation of position (see 3.2.4 page 11). A Java implementation available here: https://s.42l.fr/sortAct**



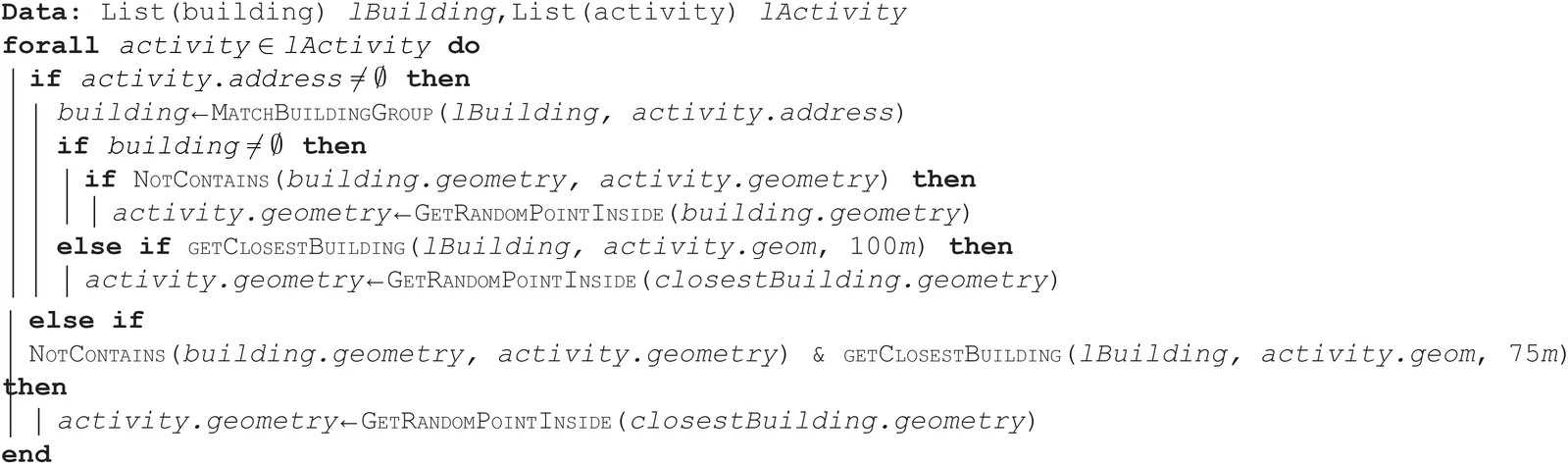



**Algorithm 4: Matching algorithm to evaluate the level of correspondence between two activities (see 3.2.4 page 14). A Java implementation available here: https://s.42l.fr/matchAct**



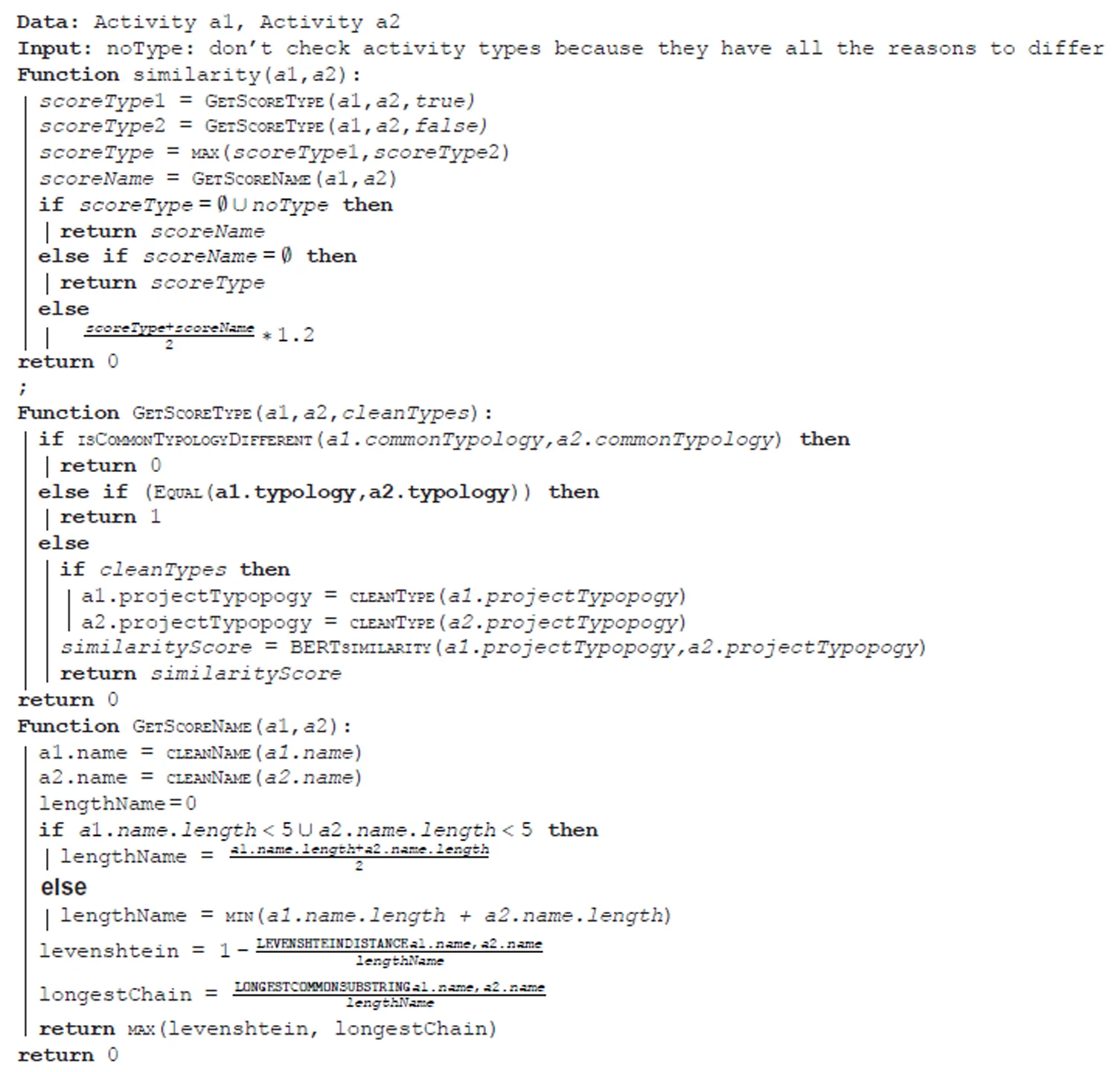



**Algorithm 5: Updating not-up-to-date dataset with an historicised dataset (see 3.2.4 page 15). A Java implementation available here: https://s.42l.fr/sortAct**



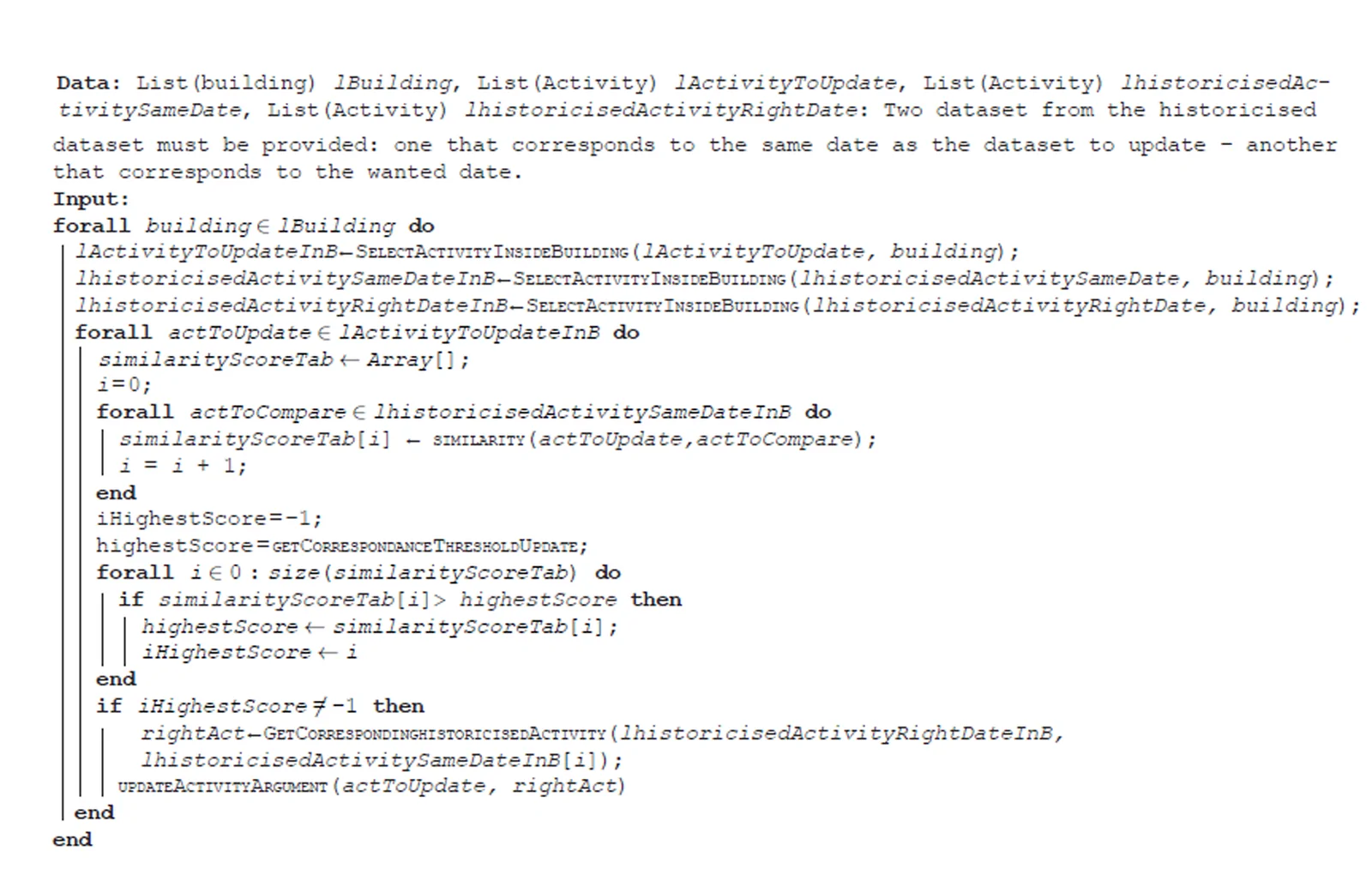



**Algorithm 6: Conflation of every activity from a single dataset (see 3.2.4 page 16). A Java implementation available here: https://s.42l.fr/sortAct**



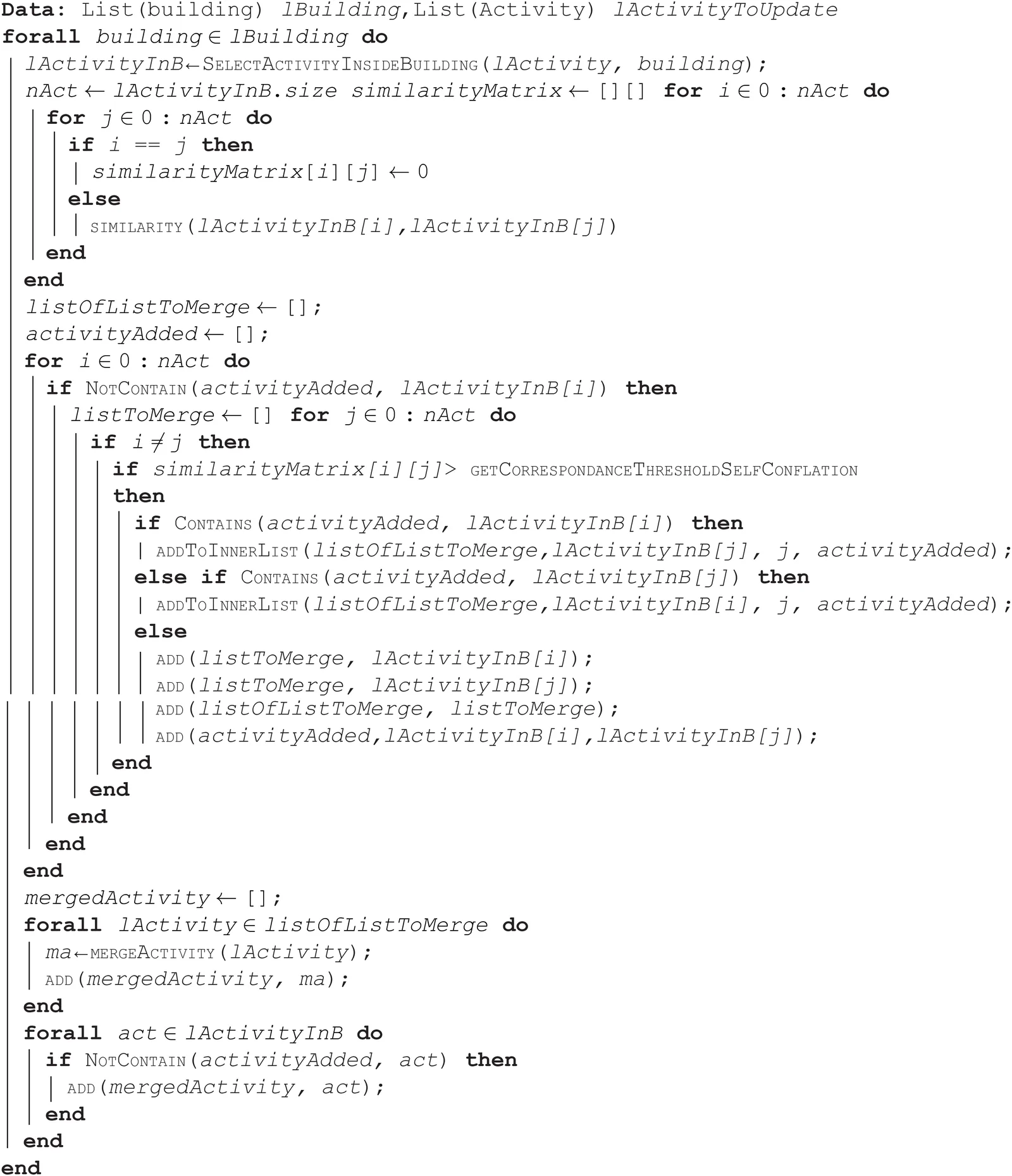



**Algorithm 7: Conflation of activities from multiple datasets (see 3.2.4 page 16). A Java implementation available here: https://s.42l.fr/sortAct**



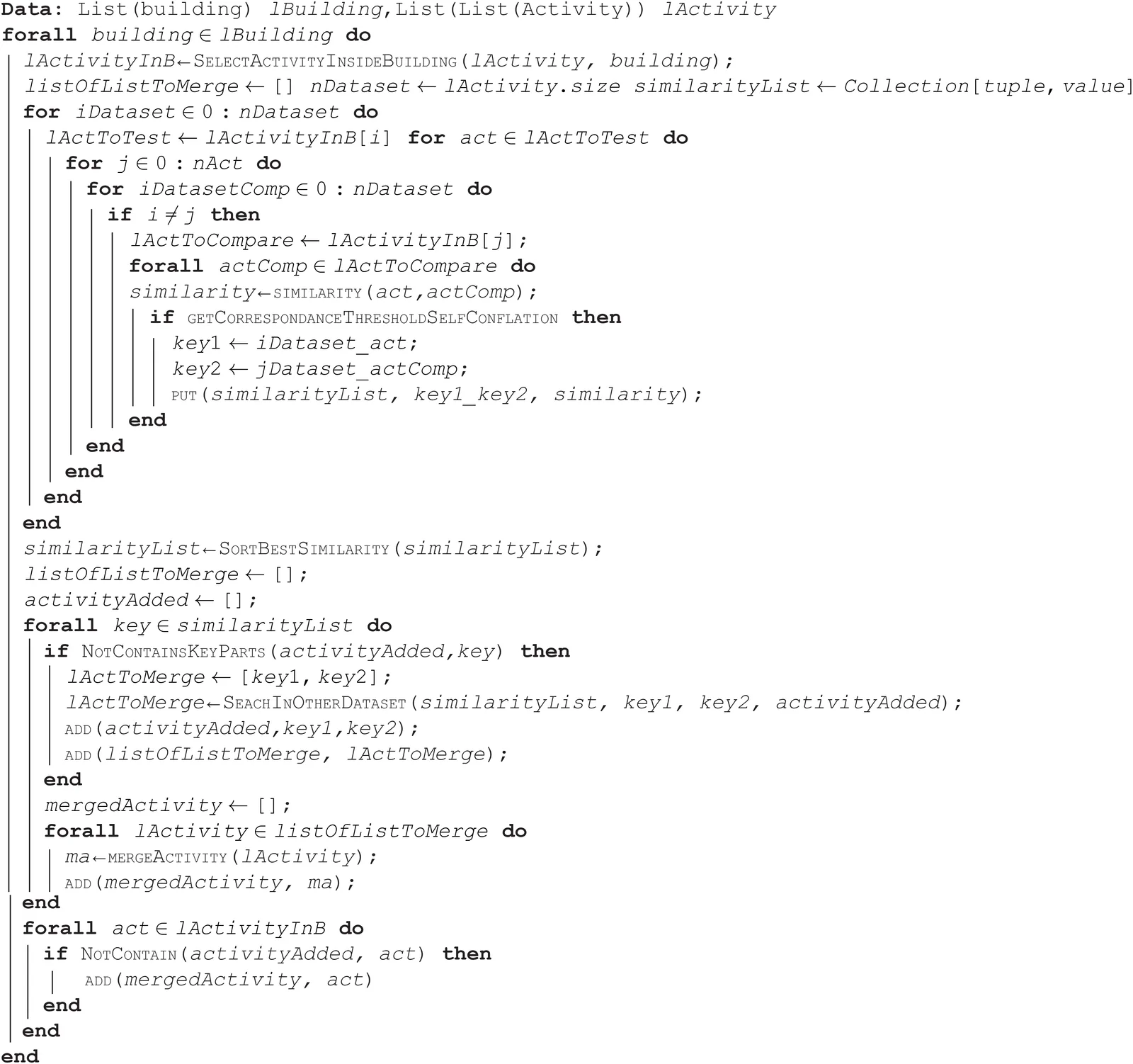



**Algorithm 8: Decide if an activity occupies a premises or not (see 3.2.4 page 16). A Java implementation available here: https://s.42l.fr/sortAct**



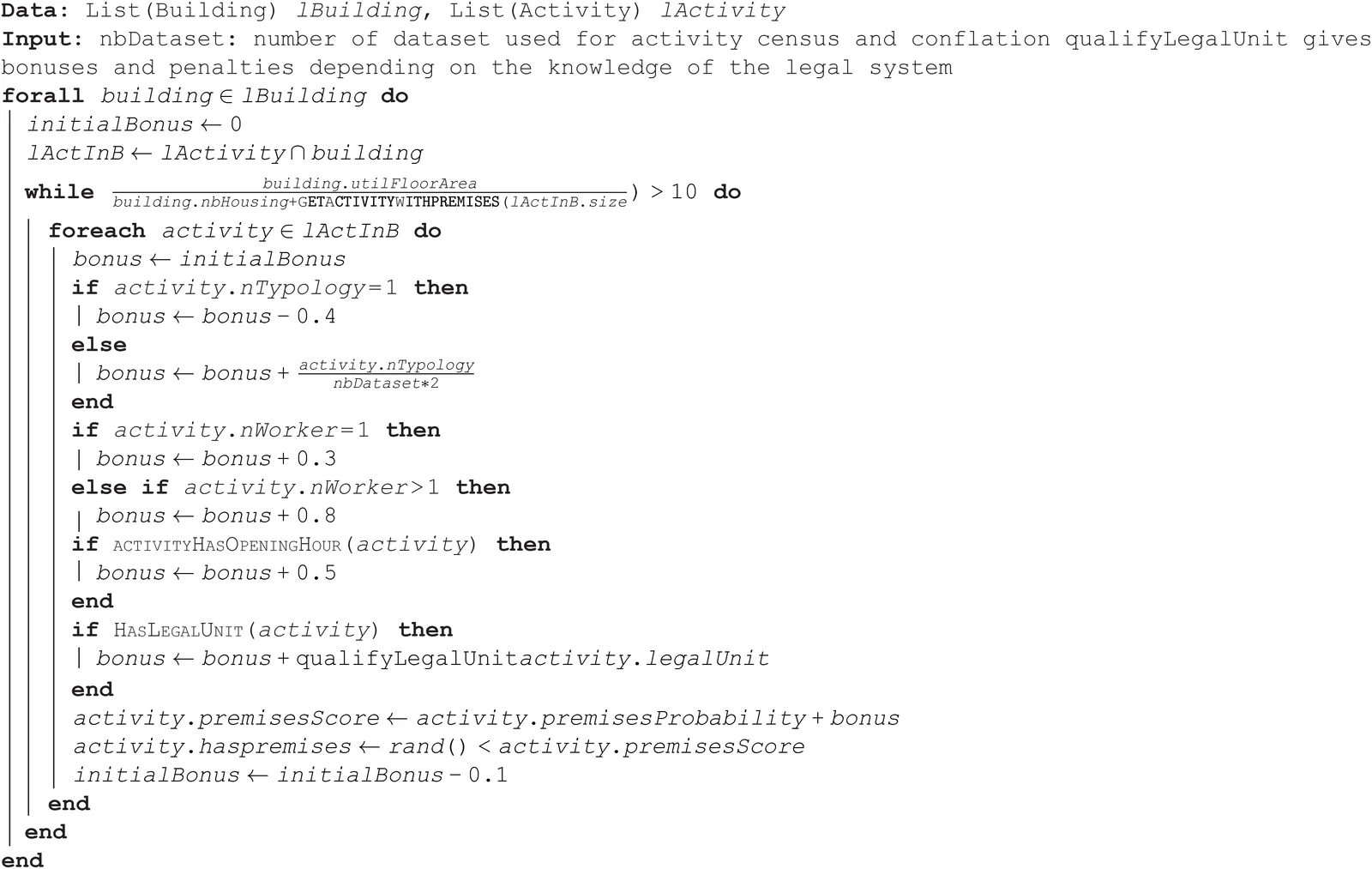



**Algorithm 9: Preset activity’s area (see 3.3.4). A Java implementation available here: https://s.42l.fr/attrSetting**



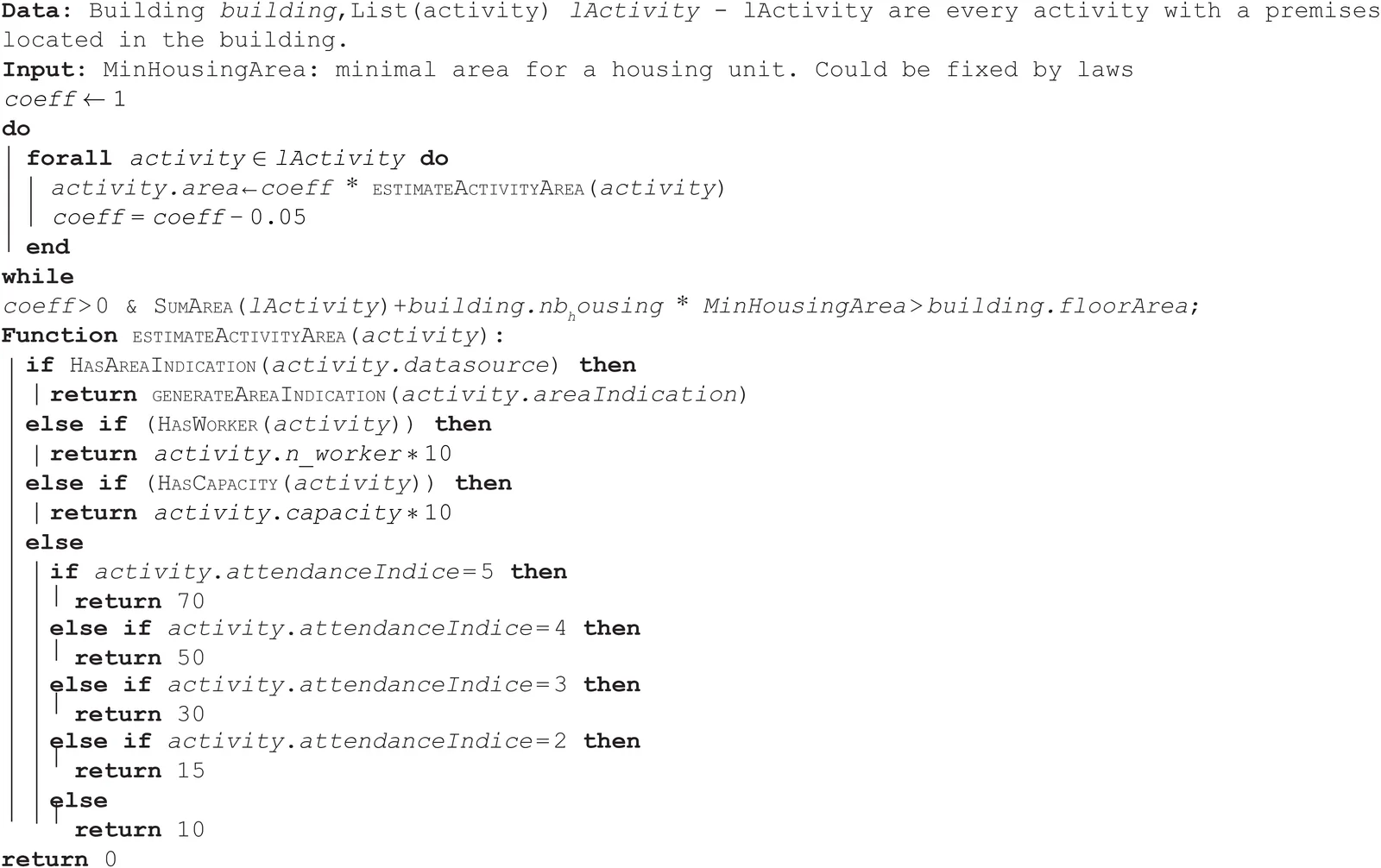



**Algorithm 10: Area attribution for housing unit (see 3.3.4) (Fig 37). A Java implementation available here: https://s.42l.fr/attrSetting**



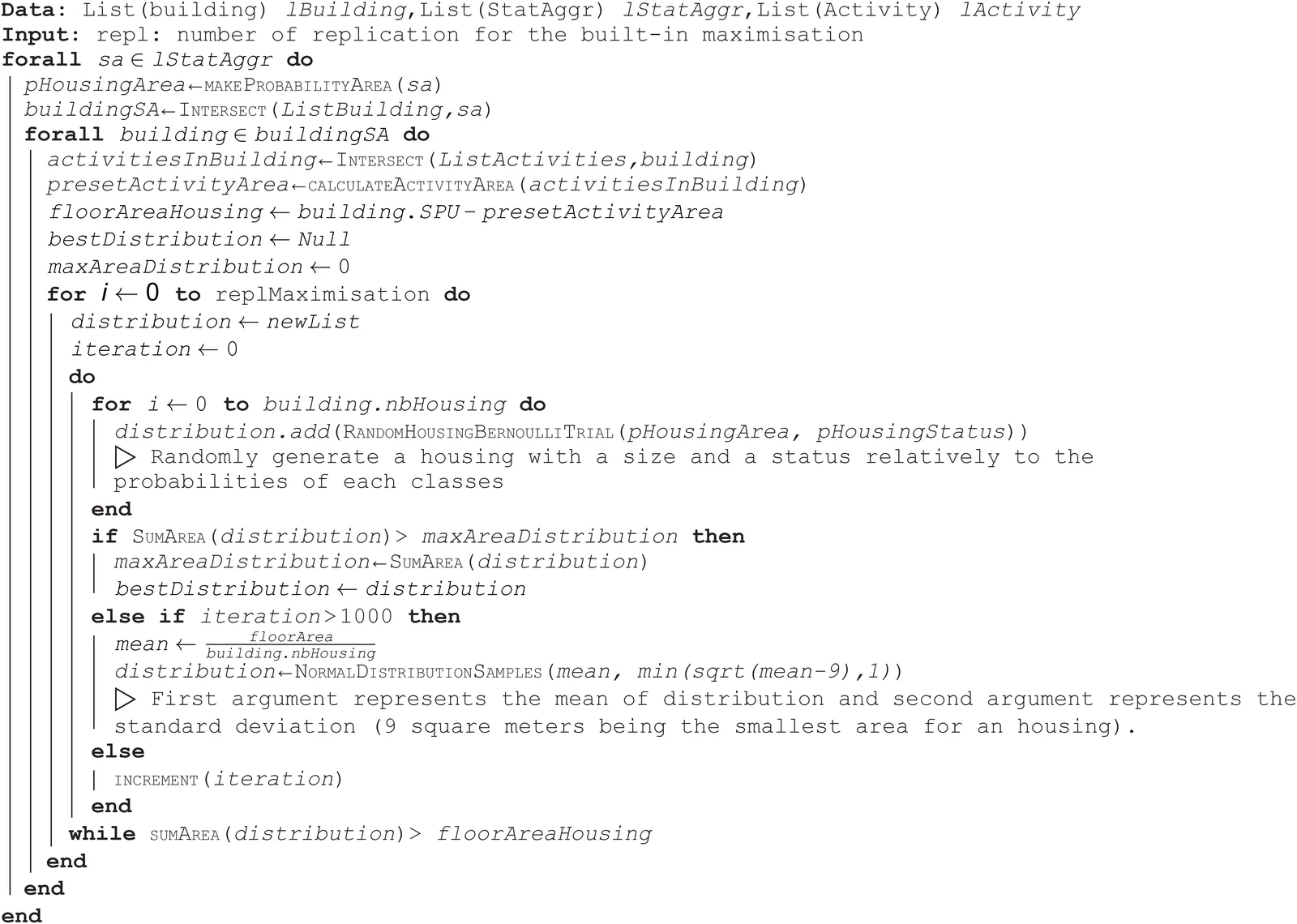



### F Additional graphs

Fig 36 shows additional graphs of the comparison of input data used to constuct building groups, presented it section 4.1.2.

Fig 37 dispays maps comparing each housing unit area class generation with statistics, as described in section 4.3.1.

**Fig 36:**
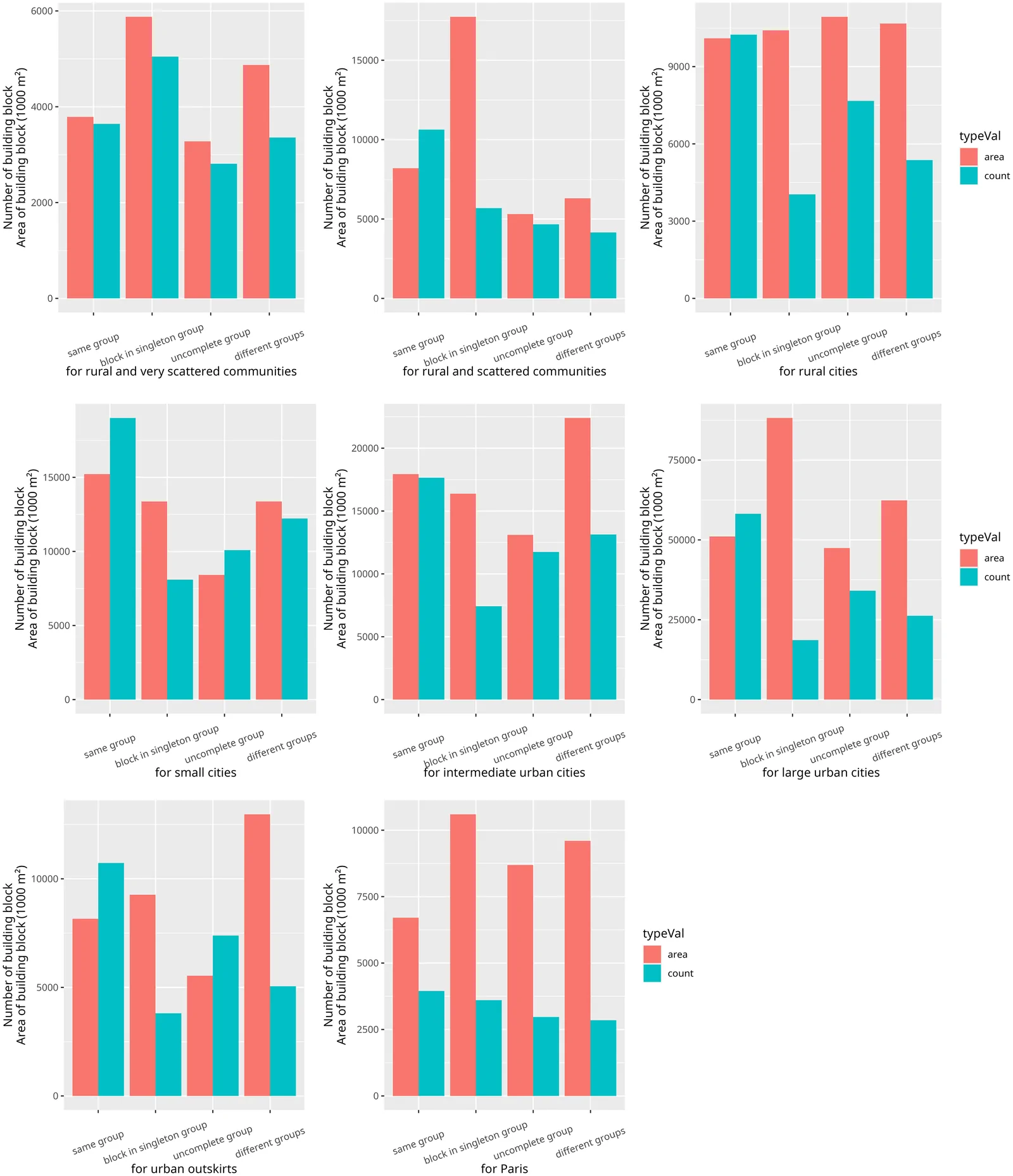
Comparison of *building group* with or without links on various typologies of French cities.

**Fig 37:**
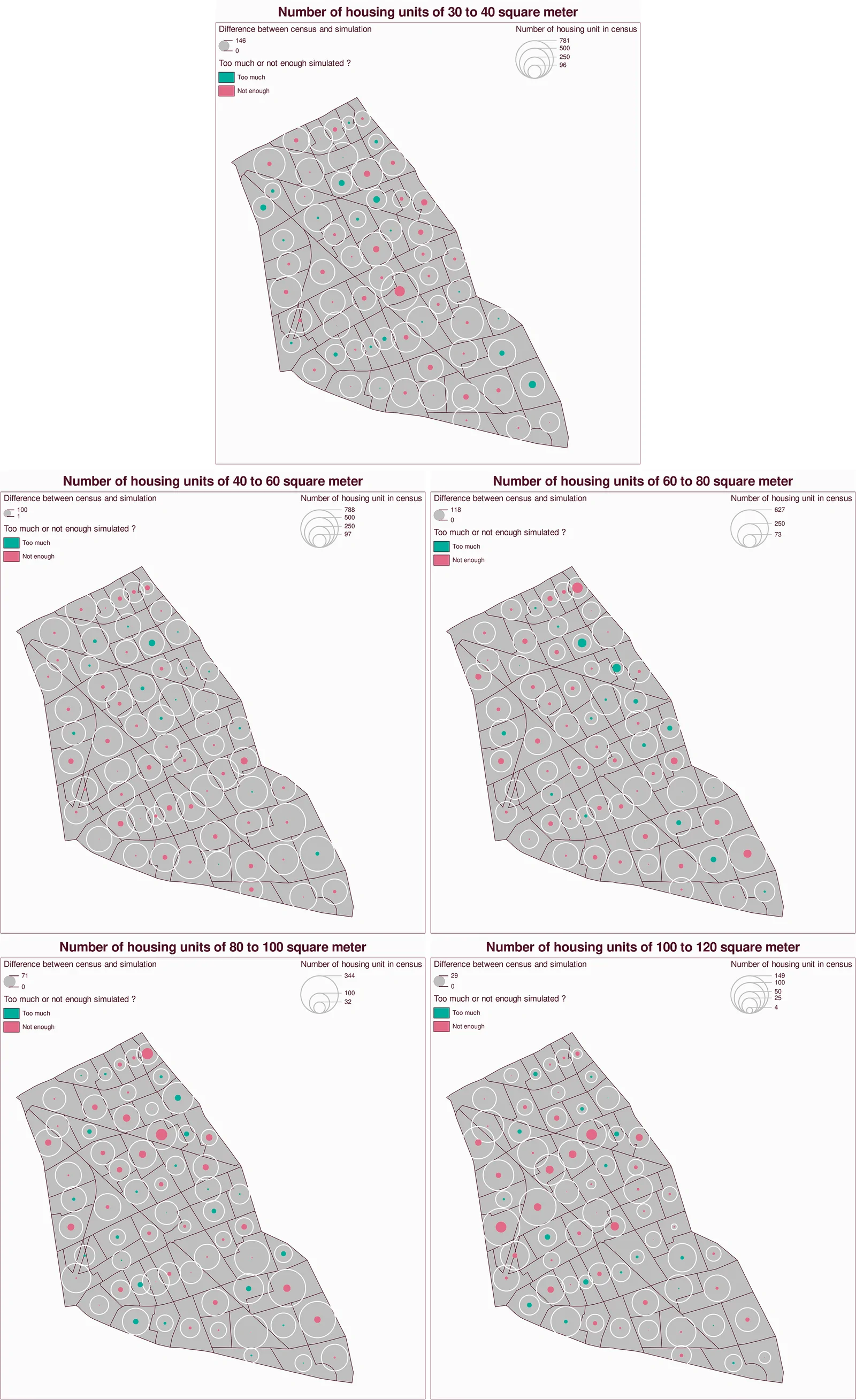
Discrepancy between generated and statistical housing units in the IRIS zones of the 11th arrondissement of Paris for multiple classes of area.
